# FLOWR.ROOT – A flow matching-based foundation model for joint multi-purpose structure-aware 3D ligand generation and affinity prediction

**DOI:** 10.1038/s41467-026-74130-9

**Published:** 2026-07-06

**Authors:** Julian Cremer, Tuan Le, Mohammad M. Ghahremanpour, Emilia Sługocka, Filipe Menezes, Djork-Arné Clevert

**Affiliations:** 1 Machine Learning & Computational Sciences, Pfizer Worldwide R&D, Berlin, Germany; 2 Computational Chemistry, Medicine Design, Pfizer Worldwide R&D, Cambridge, MA USA; 3 Doctoral School of Medical and Health Sciences, Jagiellonian University Medical College, Cracow, Poland; 4 Department of Physicochemical Drug Analysis, Faculty of Pharmacy, Jagiellonian University Medical College, Cracow, Poland; 5 Institute of Structural Biology, Molecular Targets and Therapeutics Center, Helmholtz Munich, Neuherberg, Germany; 6 TUM School of Natural Sciences, Department of Bioscience, Bayerisches NMR Zentrum, Technical University of Munich, Garching, Germany

**Keywords:** Cheminformatics, Computational chemistry, Structure-based drug design, Structure-based drug design, Lead optimization

## Abstract

We present FLOWR.ROOT, an *SE*(3)-equivariant flow-matching foundation model that unifies pocket-aware 3D ligand generation with multi-endpoint binding affinity prediction (pIC_50_, p*K*_i_, p*K*_d_, pEC_50_) and pLDDT-based confidence estimation in a single backbone. One trained model supports de novo pocket-conditional generation, interaction- and pharmacophore-conditional sampling, scaffold hopping and elaboration, and fragment growing or replacement, enabled by a mixed isotropic–anisotropic prior placement strategy. Training proceeds in three stages: large-scale pre-training on billions of ligand conformations and millions of mixed-fidelity protein–ligand complexes, refinement on curated co-crystal data, and project-specific adaptation via parameter-efficient LoRA finetuning. Joint structure–affinity modelling enables inference-time importance-sampling guidance for single- and multi-objective design without external scoring functions. Case studies on kinase selectivity (CK2*α*/CLK3) and scaffold elaboration on TYK2, ER*α*, and BACE1 illustrate utility from hit identification through lead optimization.

## Abstract (web summary)

Structure-based generative modeling is rapidly reshaping drug discovery by enabling pocket-aware ligand design alongside predictive evaluation of binding properties. This manuscript introduces FLOWR.root, an SE(3)-equivariant flow-matching framework that jointly generates high-quality 3D ligands and predicts multi-endpoint binding affinities, demonstrating state-of-the-art performance, efficient domain adaptation, and practical impact across de novo design, scaffold elaboration, and lead optimization workflows.

## Introduction

Diffusion- and flow-based generative models have become central tools for learning complex data distributions and have recently enabled rapid progress in generative chemistry and structure-based drug design (SBDD)^1–17^. One key subfield is pocket-conditional ligand generation, where *SE*(3)-equivariant models generate ligands directly in the protein binding site^11,12^. Subsequent work has improved chemical validity and pose quality and explored pre-training and inference-time guidance to steer sampling toward desired objectives^10,13,18^. Complementary to de novo design, fragment-based approaches support practical lead optimization; however, combining pocket conditioning with flexible fragment elaboration and editing (for example, scaffold replacement or fragment growing under interaction constraints) within a single generative backbone remains challenging^17,19–24^.

Beyond generation, the utility of proposed ligands depends on reliable potency and binding affinity prediction to prioritize candidates during iterative design. Classical scoring functions (for example, docking) are computationally efficient but can be insufficiently accurate or do not represent affinities, whereas more elaborate physics-based methods, such as free energy perturbation (FEP) and absolute binding free energies (ABFE), are typically more reliable but too costly to apply at the scale of modern generative campaigns^25–33^. Machine learning scoring functions improve throughput but may suffer from dataset bias and limited generalization^34–36^. Co-folding approaches such as Boltz-2 can reach high accuracy on selected targets, but require structure prediction per query and, due to decoupled training, may limit co-adaptation between pocket geometry and affinity^37^.

These considerations motivate models that jointly learn (conditional) structure generation and affinity prediction in a protein pocket context. Joint modeling supports in-distribution ranking of generated structures and enables inference-time steering (for example, via importance sampling) without relying on external scoring functions^13^. At the same time, drug discovery campaigns are characterized by assay- and series-specific structure–activity relationships (SARs) that often differ from public training data. Consequently, strong benchmark performance does not necessarily translate to a new project without adaptation; efficient finetuning and, where appropriate, preference-based alignment^38^, can help align the model to the relevant local SAR landscape.

Training such models is further constrained by the scarcity of high-quality protein–ligand complexes with reliable affinity annotations relative to ligand-only resources. This motivates multi-stage training strategies that exploit large-scale, lower-fidelity data to learn broad chemical and structural priors, followed by refinement on curated experimental datasets to improve structural accuracy and affinity prediction^39–46^.

In this work, we present FLOWR.ROOT, a foundation model for structure-based drug design that spans large-scale pre-training to project-specific adaptation. Within a single architecture, FLOWR.ROOT unifies de novo, interaction- and pharmacophore-conditional, and fragment-conditioned ligand generation with multi-endpoint affinity prediction and confidence estimation. The model employs a three-stage training strategy that leverages datasets of varying fidelity: (1) large-scale pre-training on billions of ligands and millions of protein-ligand complexes with diverse affinity labels, (2) refinement on curated, higher-quality structural data, and (3) project-specific adaptation via parameter-efficient Low-Rank Adaptation (LoRA)^47^ and multi-objective guidance through importance sampling^13^. This joint training paradigm allows affinity prediction to directly guide the generative process without external scoring functions, while supporting rapid adaptation to project-specific data. In addition, we introduce flexible fragment-conditional generation modes, including local fragment growing and replacement for spatially targeted modifications enabled by a mixed isotropic-anisotropic prior placement strategy. Combining joint structure and affinity prediction with fragment-based elaboration, affinity guidance enables effective sampling and ranking of local chemical spaces. On established benchmarks, FLOWR.ROOT achieves 0.97 PoseBusters-validity for pocket-conditional ligand generation and Pearson correlations of up to 0.86 for binding affinity prediction on the FEP+/OpenFE benchmark. Together, these capabilities position FLOWR.ROOT as a versatile tool for early-stage drug discovery, from hit identification through lead optimization.

## Results

### Overview of FLOWR.ROOT

**Fig. 1:**
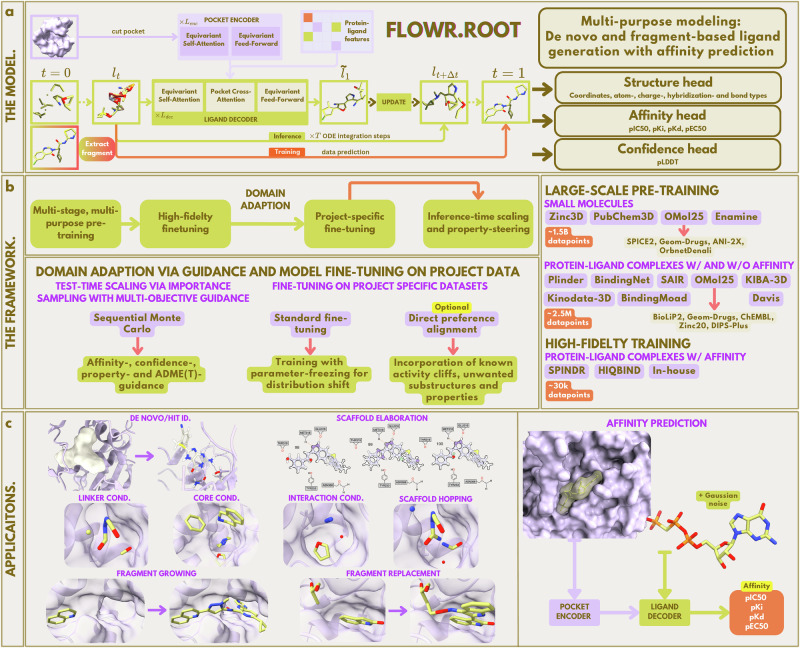
Overview of the FLOWR.ROOT framework for joint structure-aware ligand generation and affinity prediction. **a** The FLOWR.ROOT model is built on an *SE*(3)-equivariant flow matching backbone that learns a transport map transforming noise (and optionally fragment anchors) into ligand structures within protein pockets. The architecture comprises an equivariant pocket encoder processing protein pocket features, and a ligand decoder processing ligands, while ligand-pocket interactions are integrated via equivariant cross-attention. A structure head predicts atomic coordinates, atom types, bond orders, charges, and hybridization states, a multi-affinity head predicts affinity with separate predictors for pIC_50_, p*K*_i_, p*K*_d_, and pEC_50_, and a confidence head provides pLDDT-based uncertainty estimation. At inference samples are generated by numerically integrating the learned flow ODE with an Euler solver. **b** The FLOWR.ROOT framework consists of three stages combining multi-stage training with finetuning and domain adaptation. Training follows a three-stage paradigm exploiting data of varying fidelity. Large-scale pre-training on  ~ 1.5B small molecule conformations is followed by training on mixed-fidelity  ~ 2.5M protein-ligand complexes with and without affinity labels. Then, the model is finetuned on curated high-fidelity co-crystal datasets to sharpen structural accuracy and affinity prediction, followed by project-specific domain adaptation through parameter-efficient finetuning, or direct preference alignment, and inference-time scaling via importance sampling with multi-objective guidance. **c** FLOWR.ROOT supports multiple ligand generation modes within a single backbone: ligand-only and pocket-conditional de novo generation, interaction/pharmacophore-conditional generation for preserving specific protein-ligand contacts, scaffold hopping and elaboration, and local fragment growing/replacement. Given a protein pocket and a bound ligand, FLOWR.ROOT can also be used to predict potency and binding affinity.

FLOWR.ROOT is a unified framework for structure-based drug design that jointly trains pocket-aware 3D ligand generation with multi-endpoint affinity prediction and confidence estimation (Fig. 1). The framework addresses key challenges in computational drug discovery: generating chemically valid, geometrically realistic ligands within protein binding sites; accurately predicting potency and binding affinity across diverse assay types; supporting flexible generation modes from de novo design to targeted fragment modifications; and enabling efficient adaptation to project-specific structure-activity relationships. A comprehensive overview is given in Methods.

The model is built on an *SE*(3)-equivariant flow matching backbone that learns a mixed continuous/discrete transport map, transforming a prior distribution (noise or fragment anchors for coordinates, uniform for categorical features) to the target ligand distribution within a given protein pocket. The architecture comprises two main components: a pocket encoder that processes full-atom protein features through equivariant self-attention layers, generating invariant and equivariant representations; and a ligand decoder that employs equivariant self-attention for intra-ligand dependencies followed by cross-attention to integrate pocket context. Protein pockets are extracted using a 7 Å cutoff radius around binding sites, balancing computational tractability with interaction coverage. The ligand decoder features three output heads that serve complementary purposes. The structure head predicts atomic coordinates, atom types, bond orders, partial charges, and hybridization states. The multi-affinity head comprises four separate predictors for pIC_50_, p*K*_i_, p*K*_d_, and pEC_50_—explicitly avoiding treatment of heterogeneous affinity labels as interchangeable. The confidence head provides pLDDT-based uncertainty estimation for generated structures. Training the structure and affinity heads jointly on a shared backbone is what enables the downstream modes used throughout this work: importance-sampling-based multi-objective optimization, multi-target selectivity from a single model, coherent LoRA domain adaptation of structure and affinity, and affinity-guided fragment elaboration. A modular generator-plus-external-scorer pipeline cannot provide these capabilities without re-introducing the generator–scorer distribution mismatch that the joint formulation removes. A controlled structure-only vs. joint comparison on HIQBIND confirms that this comes at no cost to structural quality (Supplementary Information).

Training follows a three-stage paradigm that exploits data of varying fidelity to address the scarcity of high-quality experimental complexes: Stage 1 builds broad generative priors across chemical and structural distributions. The model trains on ~1.5B small molecule conformations (ZINC3D, PubChem3D, Enamine REAL, OMol25) for chemical coverage, and ~2.5M mixed-fidelity protein-ligand complexes with and without affinity labels from both computational sources (BindingNet, SAIR, KIBA-3D, Davis-3D, Kinodata-3D) and experimental sources (Plinder, BindingMOAD). Stage 2 adapts pre-trained weights to curated, drug-like datasets. We use SPINDR and HiQBind for multi-target fine-tuning, teaching the model fine details of protein-ligand interactions and binding affinity from high-quality co-crystal structures. Stage 3 bridges the gap between public data and project-specific structure-activity landscapes through multiple strategies: inference-time scaling via importance sampling guides generation toward desired properties (affinity, ADME/T, synthetic accessibility); parameter-efficient finetuning enables rapid adaptation while avoiding catastrophic forgetting. This multi-stage paradigm addresses a fundamental limitation: strong benchmark performance does not guarantee generalization to unseen structure-activity landscapes. Real-world drug discovery requires understanding nuanced SAR across related molecules within specific binding sites—richness uniquely captured by dense bioactivity data that differs substantially from sparse public datasets. Rather than expecting universal generalization, FLOWR.ROOT is designed as an adaptive companion that refines through sustained interaction with project-specific data.

The model is trained using mean squared error (MSE) loss for coordinates and categorical cross-entropy (CE) losses for atom types, charges, hybridization, and bond types. In addition, we introduce explicit geometric supervision through bond length and bond angle losses. The bond length loss penalizes deviations in predicted inter-atomic distances for bonded pairs. The bond angle loss uses Huber loss over valid angle triplets to preserve local molecular geometry while remaining robust to outliers. These auxiliary losses substantially reduce strain energies in generated structures. For training the affinity head, invariant and equivariant features from ligand, pocket, and their interactions via the ligand decoder’s latent representations are extracted, and gated equivariant blocks combine invariant and equivariant tensors, followed by multi-layer perceptrons (MLPs) that produce aggregated ligand, pocket, and interaction embeddings. Task-specific heads then predict each affinity type separately, with Huber loss for robust training. This design explicitly models the distinct experimental setups underlying IC_50_, *K*_i_, *K*_d_, and EC_50_ measurements rather than treating them as interchangeable.

FLOWR.ROOT supports multiple generation modes within a single backbone, enabling flexible application across hit identification and lead optimization: (1) de novo pocket-conditional generation for exploring diverse chemical matter; (2) interaction/pharmacophore-conditional generation for preserving or enforcing specific protein-ligand contacts; (3) scaffold hopping and elaboration for core and R-group replacement; and (4) fragment growing and local fragment replacement for targeted structural modifications. For the latter mode, we use a flexible prior placement strategy that shifts the generative prior from the zero center-of-mass to the local replacement site, enforcing locality in partial modifications and enabling precise control over which atoms to replace while preserving the remainder of the molecule. In addition, we propose a mixed isotropic and anisotropic Gaussian prior placement during training to enable a more targeted ligand generation strategy at inference. We provide more details in Methods. FLOWR.ROOT also supports inference-time steering via importance sampling to sample from conditional distributions, allowing for single- and multi-objective optimization without retraining, for example, steering toward higher predicted affinities while maintaining sample diversity.

To train FLOWR.ROOT, we leveraged a diverse collection of datasets. A detailed description of all datasets used in this work, including preprocessing and curation pipelines, dataset statistics, and chemical space analyses, is provided in Methods. In short, all protein-ligand complexes undergo rigorous preprocessing using Schrödinger’s LigPrep and PrepWizard, including protonation state determination at physiological pH, protein preparation with side chain completion, and constrained minimization. Protein pockets are extracted using a 7 Å cutoff radius around reference ligands. Crucially, if not stated otherwise, throughout all protein-ligand datasets, we kept a consistent dataset split following the provided Plinder^43^ train, validation, and test set splits to avoid data leakage. Plinder employs comprehensive similarity metrics (protein sequence, pocket-level Jaccard, interaction-level PLIP, and ligand-level Tanimoto) to ensure minimal leakage and high-quality, non-redundant testing. To our knowledge, this represents the most rigorous publicly available splitting strategy for structure-based modeling.

### Unconditional and pocket-conditional generation

We first establish the improvements conferred by the architectural changes incorporated into FLOWR.ROOT. To this end, we demonstrate the expressiveness of the ligand decoder backbone for unconditional 3D molecule generation on the well-established GEOM-DRUGS dataset, comparing against recent state-of-the-art models including EQGAT-DIFF^10^, SEMLAFLOW^16^, ADIT^48^, MEGALODON^49^, and FLOWMOL3^50^. Here, our non-pretrained base model, FLOWR.ROOT^base^, achieves 0.94 ± 0.02 PoseBusters-validity (mean), surpassing all baselines including the recently released FLOWMOL3 model (0.92 ± 0.07), with a median relaxation energy of 3.65 ± 0.2 kcal/mol and a median relaxation RMSD of only 0.07 ± 0.02 Å—demonstrating high geometric precision. Complete benchmark results and comparisons are provided in Supplementary Table 1.

For pocket-conditional ligand generation, we benchmark on CROSSDOCKED2020, where FLOWR.ROOT^base^ achieves a mean PoseBusters-validity of 0.97 ± 0.22 and a mean strain energy of 67.13 ± 53.05 kcal/mol, both of which are substantially lower than all competing models, including FLOWR (mean PoseBusters-validity of 0.92 ± 0.22 and mean strain energy of 87.83 ± 74.30 kcal/mol) as well as the next best model PILOT (mean PoseBusters-validity of 0.83 ± 0.33 and strain energy of 110.48 ± 87.47 kcal/mol), and the highest mean AutoDock-Vina score of  − 7.76 ± 0.55 kcal/mol (vs.  − 6.29 ± 1.56 and  − 5.73 ± 1.72 kcal/mol). Complete benchmark results and comparisons are provided in Supplementary Table 2.

### Pocket-conditional ligand generation: SPINDR

Next, we evaluate FLOWR.ROOT’s ability to generate valid, physically plausible ligands, but on the more challenging test set of the SPINDR dataset. We compare the non-pretrained FLOWR.ROOT^base^ model with FLOWR and the diffusion-based PILOT model. Table 1 reveals that FLOWR.ROOT^base^ substantially outperforms both FLOWR and PILOT across all metrics. FLOWR.ROOT^base^ achieves a mean PoseBusters-validity of 0.97 ± 0.10, nearing the test set reference (0.99 ± 0.04). Additionally, the strain energy statistics substantially decrease to 50.36 ± 34.59 kcal/mol, further demonstrating the model’s ability to generate chemically valid and energetically favorable ligands. FLOWR.ROOT^base^ also performs better in docking accuracy, with a mean Vina score of −7.52 ± 0.84 kcal/mol.

**Table 1:** Evaluation and comparison of FLOWR.ROOT with PILOT and FLOWR on SPINDR

Model	PB-valid *↑*	Strain energy *↓*	Vina score *↓*	Vina score^min^ *↓*	BondA.W1 *↓*	BondL.W1 [10^−2^] *↓*	DihedralW1 *↓*
PILOT	0.79 ± 0.21	120.10 ± 71.61	−6.30 ± .96	−6.68 ± 1.07	1.82	0.42	5.52
FLOWR	0.93 ± .22	90.05 ± 52.18	−6.93 ± 0.92	−7.22 ± 0.92	1.08	0.35	3.88
FLOWR.ROOT^base^	0.97 ± .10	50.36 ± 34.59	−7.52 ± 0.84	−7.71 ± 0.85	0.60	0.43	3.62
Test set	0.99 ± 0.04	43.27 ± 41.85	−7.69 ± 2.00	−7.88 ± 2.00	-	-	-

Benchmark comparison of the non-pretrained FLOWR.ROOT^base^ model against FLOWR and the diffusion-based PILOT model on the SPINDR test dataset. We sample *n* = 100 ligands per target across *n* = 225 SPINDR test-set targets. The evaluation includes PoseBusters-validity (PB-valid), strain energy calculated using GenBench3D, and AutoDock-Vina scores (kcal/mol). Additionally, we report the Wasserstein distance of the generated ligands’ bond angles (BondA.W1), bond lengths (BondL.W1) and dihedral angles (DihedralW1) distributions relative to those in the SPINDR test set. Ligand sizes for all models are sampled uniformly with a −10%/+10% margin around the respective reference ligand size. Values denote the mean across ligands and targets; subscripts denote the standard deviation across targets.

In addition, we analyzed a wide range of complementary metrics (Supplementary Tables 3–6). FLOWR.ROOT^base^ achieves 100% novelty (1.00 ± 0.00) with high diversity (0.83 ± 0.10) and uniqueness (0.89 ± 0.18) rates, comparable to FLOWR, while demonstrating consistent and substantial performance improvements across fragment-conditional generation modes, including scaffold hopping and pharmacophore-conditional generation.

### Affinity prediction: HIQBIND

**Fig. 2:**
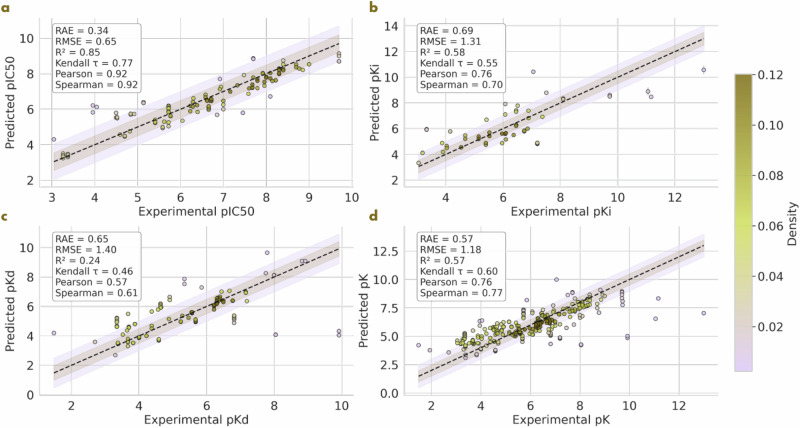
Affinity prediction performance of FLOWR.ROOT on the HIQBIND test dataset. Correlation plot of FLOWR.ROOT-predicted pIC_50_ vs. experimental pIC_50_ binding affinities across protein-ligand complexes on the HIQBIND test set (based on Plinder splits; *n* = 278). **a** Correlation with experimental pIC_50_ affinities (*n* = 116). **b** Correlation with experimental p*K*_i_ affinities (*n* = 54). **c** Correlation with experimental p*K*_d_ affinities (*n* = 108), and **d** shows the correlation results if the median of all predicted affinities is used (*n* = 278). The numeric metrics (RAE, RMSE, R^2^, Kendall *τ*, Pearson, Spearman) reported in each panel inset are means across *n* = 4 random-seed runs; standard deviations across seeds are depicted as vertical error bars per point. Shaded bands depict fixed reference envelopes at ±0.5 and  ± 1 kcal/mol from the diagonal (not statistical error bands); color encodes the local density of predictions (darker = denser). Each plotted point (mean value across seed runs for panels **a–c** and median value for (**d**) corresponds to a single protein–ligand complex. Source data are provided as a Source Data file.

A central part of FLOWR.ROOT is its joint structure-affinity modeling. In Fig. 2 we provide a detailed analysis of affinity prediction performance on the HIQBIND test set. The affinity head of FLOWR.ROOT provides accurate predictions across various affinity types, including pIC_50_, p*K*_i_, and p*K*_d_. For pIC_50_, the model achieves a Pearson correlation of 0.92 ± 0.03 and an R^2^ of 0.85 ± 0.06, while for p*K*_i_ and p*K*_d_ we get Pearson correlations of 0.76 ± 0.13 and 0.57 ± 0.20, respectively. When aggregating predictions across all affinity types (median), the model achieves RAE 0.58 ± 0.06, RMSE 1.18 ± 0.18, R^2^ 0.57 ± 0.10, Kendall *τ* 0.60 ± 0.06, and Pearson 0.76 ± 0.07. Note, reported values are mean values across four seed runs  ± the mean 95% confidence interval (CI) across seeds. These results confirm that the affinity head, trained jointly with structure generation, provides accurate and reliable potency estimates with robust performance across different experimental affinity labels. Probing the FLOWR.ROOT^base^ model trained solely on HIQBIND without pre-training, we observe substantially worse performance across all metrics with RAE 0.70 ± 0.13, RMSE 1.95 ± 0.24, R^2^ 0.39 ± 0.13, Kendall *τ* 0.49 ± 0.09, and Pearson 0.63 ± 0.10, underlining the importance of the proposed large-scale pre-training pipeline. Note, a structure-only ablation trained under identical conditions matches the joint model on HiQBind structural metrics within seed-level noise: mean PoseBusters-validity 0.94 vs. 0.93 with a seed-level standard deviation of each 0.02 across three seeds. We find no evidence that the joint structure-affinity objective significantly degrades structural quality. Note, in both cases, we strictly follow the Plinder dataset split, minimizing information leakage between train and test also when pre-training.

**Fig. 3:**
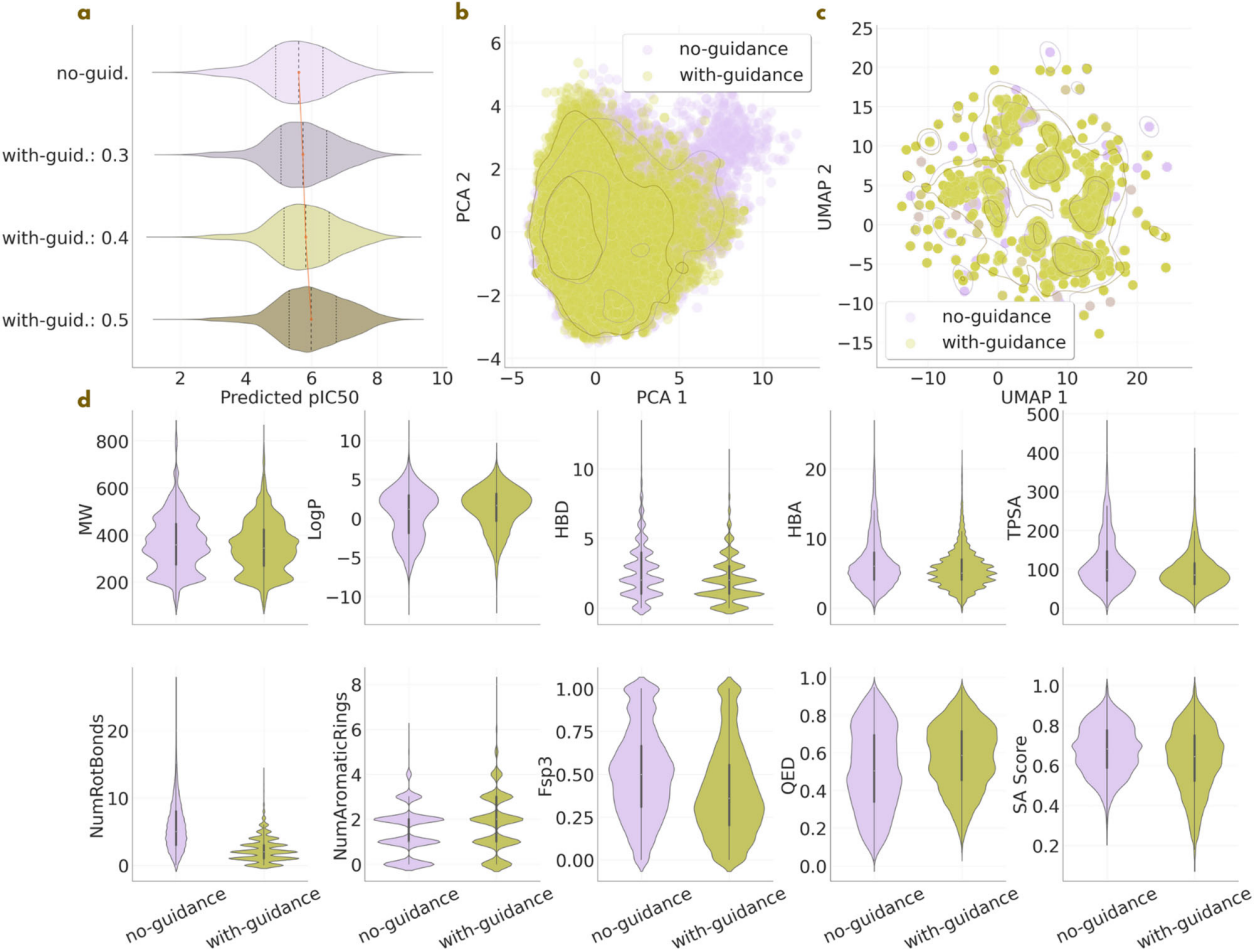
Evaluation of FLOWR.ROOT on inference-time steering performance using affinity-guided importance sampling on the HIQBIND test dataset. **a** Comparing the distribution of pIC_50_ predictions of generated ligands across test set targets between un-guided, and mild to strongly guided steering (0.3–0.5 guidance strength). **b** Depiction of chemical space comparison between un-guided and guided samples (0.5) via PCA analysis showing first two principal components. **c** Depiction of chemical space comparison between un-guided and guided samples (0.5) via 2D UMAP analysis. **d** Distribution comparison between un-guided and guided samples (0.5) regarding different chemical properties, namely molecular weight (MW), logP, number of hydrogen donors (HBD) and acceptors (HBA), topological surface area (TPSA), number of rotatable bonds (NumRotBonds) and aromatic rings (NumAromaticRings), fraction of *sp*^3^ carbons (Fsp3), druglikeness (QED) and synthesizability (SA Score). All reported distributions are descriptive. Violin plots in (**a**) and (**d**) aggregate *n* = 100 ligands sampled per target across *n* = 278 HIQBIND test-set targets. Within each violin, the white dot marks the median, the thick central bar the interquartile range (25th–75th percentile), the thin line extends to 1.5 × IQR (whiskers), and the outer envelope spans the full data range. Source data are provided as a Source Data file.

Beyond predictive accuracy, FLOWR.ROOT enables property-guided generation via inference-time steering. Figure 3 illustrates the effect of importance sampling-based steering on the predicted affinity distribution of generated ligands on the HIQBIND test set. Without guidance, the mean predicted pIC_50_ is 5.60 ± 1.11. As the steering duration increases (from 0.3 to 0.5 of the trajectory), the mean predicted pIC_50_ shifts progressively to higher values (5.75, 5.84, 6.02), while the standard deviation remains stable. This demonstrates that the model can be effectively biased toward predicted higher-affinity ligands during generation.

In Fig. 3, we show the distribution of important chemical properties comparing unguided with guided (0.5 steering duration) samples across targets on the HIQBIND test set. While the distribution of SA scores shows a slight decrease towards lower scores for guided samples, we find that the overall druglikeness increases substantially, with a mean Lipinski score of 4.30 ± 1.04 for the unguided to 4.68 ± 0.65 for the guided samples. Interestingly, we find that guidance consolidates the sample space, leading to mostly more compact distributions. This can also be seen in the chemical space comparison in Fig. 3**b**, **c**, where we overlay the chemical spaces of the unguided and guided samples using both PCA and UMAP on molecular fingerprints.

Lastly, we also analyze geometric validity and sample diversity as steering intensity increases. For unguided versus guided samples, we observe a mean PoseBusters-validity of 0.93 ± 0.20 versus 0.91 ± 0.22, mean strain energy of 48.46 ± 32.29 versus 54.42 ± 36.17 kcal/mol, mean Vina score of −7.41 ± 0.86 vs. −7.50 ± 0.85, and mean per-target diversity of 0.79 ± 0.09 versus 0.79 ± 0.10. While we observe a slight decrease in PoseBusters-validity and an increase in strain energies, the diversity of the samples remains the same, so we can rule out a potential mode collapse when using guidance. In addition, we see a slight decrease in Vina scores on guided samples, demonstrating that we can maintain physically realistic structures while optimizing for affinity.

### Affinity prediction: SCHRODINGER FEP+ AND OPENFE

Next, we evaluate the performance of the affinity prediction module of FLOWR.ROOT using the Schrodinger FEP+/OpenFE IndustryBenchmark datasets^51^, comparing FLOWR.ROOT to state-of-the-art methods. These benchmarks offer a robust test of the model’s prediction capabilities with experimentally determined binding affinities across various targets and affinity types for protein-ligand complexes.

**Fig. 4:**
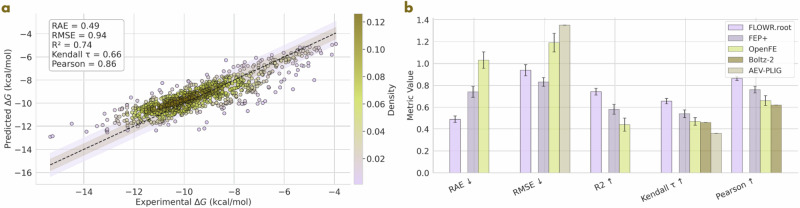
Affinity prediction performance of FLOWR.ROOT on the FEP+/OpenFE IndustryBenchmark dataset. **a** Correlation plot of FLOWR.ROOT-predicted binding affinities as median over p*K*_d_, p*K*_i_ and pIC_50_ in kcal/mol vs. experimental binding affinities across protein-ligand complexes (*n* = 881). The numeric metrics (RAE, RMSE, R^2^, Kendall *τ*, Pearson) reported as inset are means across *n* = 4 random-seed runs; standard deviations across seeds are depicted as vertical error bars per point. Shaded bands depict fixed reference envelopes at  ± 0.5 and  ± 1 kcal/mol from the diagonal (not statistical error bands); color encodes the local density of predictions (darker = denser). **b** Mean values of different correlation metrics with error bars indicating the 95% confidence interval comparing FLOWR.ROOT with FEP+, OpenFE, AEV-PLIG, and Boltz-2; FLOWR.ROOT values are means across *n* = 4 random-seed runs, and error bars denote the mean 95% confidence interval across seeds; FEP+ and OpenFE values and 95% confidence intervals are reproduced from ref. ^52^; Boltz-2 and AEV-PLIG provide single-point estimates without replicate runs; for FEP+, OpenFE, AEV-PLIG, and Boltz-2, the bars represent single-point estimates as reported in the cited literature. Source data are provided as a Source Data file.

In Fig. 4**a**, we present the scatter plot for FLOWR.ROOT-predicted combined affinity predictions (median over p*K*_d_, p*K*_i_, and pIC_50_) versus experimental binding affinities. The plot indicates a strong correlation with minimal deviation, suggesting that FLOWR.ROOT can successfully predict binding affinities across a broad range of protein-ligand complexes. In Fig. 4**b**, we show the performance of FLOWR.ROOT using various error and correlation metrics in comparison to OpenFE, FEP+, AEV-PLIG, and Boltz-2. FLOWR.ROOT achieves an RMSE of 0.93 ± 0.05 kcal/mol, with a Pearson correlation of 0.86 ± 0.02 and a Kendall *τ* of 0.65 ± 0.03. These results surpass those of FEP+ (RMSE =0.83 ± 0.04, Pearson =0.76 ± 0.03) and OpenFE (RMSE =1.19 ± 0.08, Pearson =0.66 ± 0.04), as well as those of Boltz-2 (Kendall *τ*  = 0.46, Pearson =0.62) and AEV-PLIG (RMSE =1.35, Kendall *τ*  = 0.36). Note, reported values for FLOWR.ROOT are mean values across four seed runs  ± the mean 95% CI. For FEP+ and OpenFE, the mean  ± 95% CI is shown^52^, while Boltz-2 and AEV-PLIG do not provide confidence intervals.

FLOWR.ROOT outperforms physics-based models, namely OpenFE and FEP+, on almost all error and correlation metrics and substantially outperforms recent state-of-the-art ML-based models AEV-PLIG and Boltz-2 across all metrics. Notably, FLOWR.ROOT achieves this while being 3x faster than AEV-PLIG, 200x faster than Boltz-2, and over 10000x faster than OpenFE and FEP+. However, we also want to emphasize that the Schrodinger FEP+/OpenFE dataset is likely substantially overlapping with the training data in terms of ligand and target space. While this applies to AEV-PLIG and Boltz-2 as well, allowing for a somewhat fair comparison, we do not expect these results to indicate substantial generalization capabilities.

### Domain adaptation via finetuning: in-house data

Next, we investigate the performance of FLOWR.ROOT on proprietary project-specific data. We evaluate four diverse pharmaceutical projects spanning inflammatory and neurodegenerative disease, to oncology, each encompassing distinct protein targets and ligand chemical spaces ranging from conventional small molecules to molecular degraders. Dataset sizes vary across projects: Project 1 comprises 1,073 compounds, Project 2 contains 730 compounds, Project 3 includes 229 compounds, and Project 4 contains 618 compounds. Projects 1–3 are annotated with pIC_50_ values, while Project 4 uses pEC_50_ measurements.

Pharmaceutical projects typically generate substantially more bioactivity data than co-crystal structures. To leverage existing assay data, we developed a structure generation workflow using OpenEye’s docking tools^53^. Measured compounds were first matched to existing co-crystal ligands via maximum common substructure (MCS) alignment and assigned to protein structures with the highest MCS similarity. Depending on the TanimotoCombo similarity between the query molecule and reference ligand (threshold TC ≥1.5, computed via ROCS overlays^54^ on up to 800 Omega-generated conformers^55^), we employed either Hybrid or ShapeFit docking. Docked poses were subsequently minimized using OpenEye’s Szybki with the ff14sb force field^56,57^, and poses with positive interaction energies were discarded. The top pose was selected based on RMSD to the reference ligand.

**Fig. 5:**
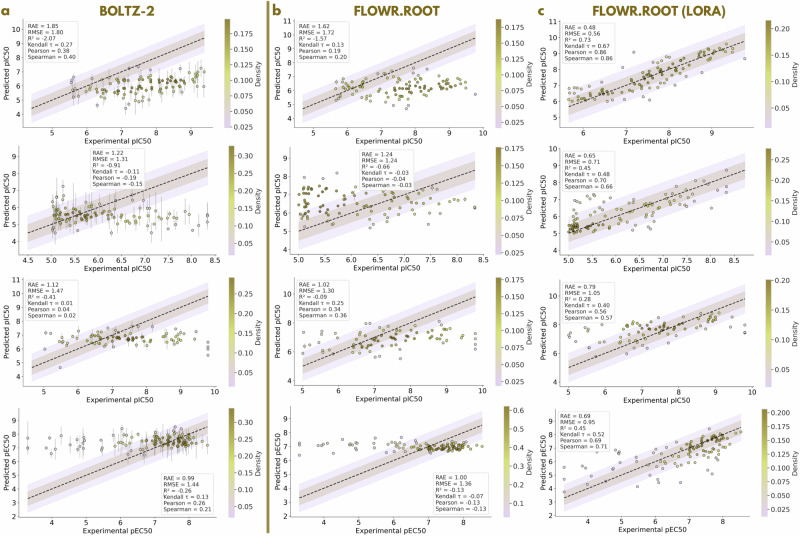
Evaluation of the affinity prediction performance of FLOWR.ROOT on a diverse set of unseen in-house structure—activity datasets with and without LoRA-finetuning. across protein-ligand complexes per project. Correlation plots of BOLTZ-2- and FLOWR.ROOT-predicted affinities vs. experimental measurements across protein-ligand complexes on four different projects. Each dot denotes the mean across *n* = 2 (Boltz-2) or *n* = 4 (FLOWR.ROOT) random-seed runs; error bars denote the standard deviation across seeds. We evaluate and compare Boltz-2 and FLOWR.ROOT and LoRA-finetuned FLOWR.ROOT on root absolute error (RAE), root mean squared error (RMSE), R-squared (R^2^), Kendall *τ*, Pearson, and Spearman correlation. Each row corresponds to a project (Projects 1–4), spanning distinct protein targets, therapeutic areas, ligand spaces (from small molecules to degraders), and structure-activity relationship (SAR) landscapes. Evaluation performed on held-out test sets: *n* = 100 complexes for Projects 1, 2, 4 and *n* = 80 complexes for Project 3. Shaded bands depict fixed reference envelopes at  ± 0.5 and  ± 1 kcal/mol from the diagonal (not statistical error bands); color encodes the local density of predictions (darker = denser). **a** Boltz-2 predictions. **b** Zero-shot FLOWR.ROOT predictions. **c** LoRA-finetuned FLOWR.ROOT predictions. Source data are provided as a Source Data file.

We evaluate both Boltz-2 and FLOWR.ROOT in zero-shot settings on held-out test sets comprising 100 randomly selected complexes per project (80 for Project 3 due to its smaller size), with the remaining samples used for training and validation. As shown in Fig. 5**a, b**, both models fail to generalize to these unseen bioactivity spaces, yielding negligible or negative correlations between predicted and experimental potencies. Specifically, Boltz-2 yields strongly negative *R*^2^ across all projects; for FLOWR.ROOT, *R*^2^ values are −1.58 ± 0.64 (Project 1), −0.67 ± 0.45 (Project 2), −0.10 ± 0.26 (Project 3), and −0.13 ± 0.12 (Project 4). These results highlight a fundamental limitation: zero-shot generalization across arbitrary structure–activity landscapes is unrealistic given the inherent complexity of structure–activity relationships.

However, FLOWR.ROOT distinguishes itself through its capacity for straightforward yet effective finetuning, enabling adaptation to the underlying data distribution and improved modeling of the structure–activity landscape. We finetuned the model using Low-Rank Adaptation (LoRA)^47^, where weight updates are parameterized as Δ*W* = *BA* with $B\in {{\mathbb{R}}}^{d\times r}$ and $A\in {{\mathbb{R}}}^{r\times k}$, using rank *r* = 16 and scaling factor *α* = 32. This configuration yields approximately 3.3M trainable parameters, representing approximately 9% of the full model weights. Models were trained for 200 epochs with validation on 50 held-out samples. Since our proprietary datasets comprise pIC_50_- and pEC_50_-annotated data points, only the respective affinity head and the backbone were finetuned.

As shown in Fig. 5**c**, LoRA-finetuned FLOWR.ROOT achieves strong performance across all four projects: Project 1 (R^2^ = 0.73 ± 0.11, Pearson *r* = 0.86 ± 0.05), Project 2 (R^2^ = 0.45 ± 0.21, Pearson *r* = 0.70 ± 0.12), Project 3 (R^2^ = 0.28 ± 0.16, Pearson *r* = 0.56 ± 0.13), and Project 4 (R^2^ = 0.45 ± 0.20, Pearson *r* = 0.69 ± 0.11). All reported values denote mean values across four seed runs  ± the mean 95% CI.

These findings underscore a critical observation: while achieving broad coverage of chemical space through ligand generation presents manageable challenges (see Supplementary Fig. 7), generalizing across structure–activity landscapes constitutes a fundamentally more complex problem. The consistent success of LoRA-finetuned FLOWR.ROOT across four therapeutically and chemically distinct projects, and both IC_50_ and EC_50_ endpoints, combined with the failure of both Boltz-2 and FLOWR.ROOT to generalize despite strong public benchmark performance supports our position that SBDD models should function as adaptive tools requiring calibration to project-specific SAR patterns. Thus, rather than expecting models to generalize universally from a single training phase, continuous refinement should be regarded as an integral component of the discovery process. Model utility grows through sustained interaction with project-specific data, positioning generative models as adaptive companions rather than standalone tools.

### Domain adaptation via finetuning: PDE10A Benchmark

Next, to complement our in-house validation, we evaluate on the publicly available PDE10A benchmark dataset introduced by Tosstorff et al.^58^. This high-quality dataset comprises 1,162 Phosphodiesterase-10A (PDE10A) inhibitors with experimentally determined IC_50_ values, curated from a former Roche PDE10A project with consistent assay conditions. The benchmark includes multiple evaluation strategies: a random split and three temporal splits (2011, 2012, 2013) that partition compounds by date, thereby simulating realistic prospective prediction scenarios encountered in drug discovery campaigns.

Table 2 presents results comparing the 2D3D hybrid QSAR method from Tosstorff et al.^58^, Boltz-2, zero-shot FLOWR.ROOT, and LoRA-finetuned FLOWR.ROOT. LoRA-finetuned FLOWR.ROOT consistently achieves the best performance across all splits and metrics. On the random split, LoRA finetuning yields RMSE = 0.31 ± 0.06 and Spearman = 0.96 ± 0.01, substantially outperforming Boltz-2 and the 2D3D hybrid method. Importantly, this strong performance is maintained on the more challenging temporal splits that assess prospective prediction capability: for temporal 2013, LoRA-finetuned FLOWR.ROOT achieves RMSE = 0.44 ± 0.17 and Spearman = 0.95 ± 0.04.

**Table 2:** Evaluation of the affinity prediction performance of FLOWR.ROOT with and without LoRA-finetuning on PDE10A

	Random		Temp. 2011		Temp. 2012		Temp. 2013	
Model	RMSE	Spear.	RMSE	Spear.	RMSE	Spear.	RMSE	Spear.
2D3D hybrid	0.85 ± 0.11	0.72 ± 0.07	0.81 ± 0.07	0.57 ± 0.09	1.25 ± 0.14	0.56 ± 0.12	0.95 ± 0.15	0.61 ± 0.18
Boltz-2	0.85 ± 0.09	0.75 ± 0.07	0.83 ± 0.07	0.59 ± 0.08	0.73 ± 0.09	0.83 ± 0.06	0.95 ± 0.22	0.74 ± 0.15
FLOWR.ROOT	1.14 ± 0.11	0.67 ± 0.08	0.94 ± 0.10	0.64 ± 0.09	1.03 ± 0.12	0.68 ± 0.11	1.03 ± 0.22	0.70 ± 0.16
FLOWR.ROOT^LoRA^	**0.32** ± 0.06	**0.97** ± 0.01	**0.33** ± 0.06	**0.93** ± 0.03	**0.43** ± 0.10	**0.93** ± 0.04	**0.44** ± 0.16	**0.95** ± 0.04

Benchmark comparison on the PDE10A dataset^58^ comprising 1,162 inhibitors with experimentally determined pIC_50_ values. We adapt the four split strategies: random split and three temporal splits (2011, 2012, 2013) that assess prospective prediction capability. Results compare the 2D3D hybrid method from Tosstorff et al.^58^, Boltz-2, zero-shot FLOWR.ROOT, and LoRA-finetuned FLOWR.ROOT. We report root mean squared error (RMSE) as well as Spearman correlation (Spear.) metrics following^58^. Subscripts denote 95% bootstrap confidence intervals. Bold values denote the best result per metric column.

Note, the 2D3D HYBRID approach proposed in Tosstorff et al.^58^ is a trained model on the respective split. Further, PDE10A is a well-studied target, thus is present in public databases and likely overlaps with Boltz-2’s comprehensive training data, making this an unreliable evaluation of true generalization. Nevertheless, these results further demonstrate and underline the effectiveness of parameter-efficient domain adaptation via LoRA finetuning within the FLOWR.ROOT framework, consistently transforming unreliable zero-shot predictions into accurate affinity estimates. In Supplementary Figs. 8 and 9, we demonstrate the fragment growing mode of LoRA-finetuned FLOWR.ROOT applied to a co-crystal structure (PDB 5SF4) from the PDE10A dataset. The generated ligands are annotated with affinity predictions to enable subsequent ranking and selection, illustrating the potential utility of our proposed framework in structure-based drug design workflows.

### Case studies

To demonstrate the practical utility and versatility of FLOWR.ROOT for structure-based drug design, we present three case studies that systematically evaluate different aspects of the framework’s capabilities. First, we investigate multi-objective optimization through a kinase selectivity study, where we simultaneously maximize binding affinity for the on-target kinase CK2*α* while minimizing off-target activity against CLK3. This case study demonstrates how FLOWR.ROOT can address one of the most challenging aspects of kinase drug discovery-achieving selectivity among structurally homologous ATP-binding sites.

Second, we benchmark the framework’s conditional ligand generation performance against quantum mechanical binding energy calculations using TYK2 kinase, ER*α* and BACE1 as model systems. These validation studies assess the correlation between FLOWR.ROOT-predicted binding affinities and computationally demanding QM binding energies, while providing mechanistic insights into the structural features that govern binding affinity in the generated ligands.

### Ligand selectivity: CK2*α* and CLK3

The human kinome consists of over 500 kinases^59^ that share conserved catalytic domain structures, particularly in their ATP-binding sites, where most competitive inhibitors bind. This structural homology, while functionally important for cellular signaling pathways, presents a major challenge for kinase selectivity-the ability of small molecule inhibitors to achieve potent inhibition of specific kinase targets while avoiding off-target interactions with structurally similar kinases. To demonstrate the capability of our framework for multi-objective optimization, we perform a selectivity study targeting selectivity between CK2*α* (on-target, PDB 3PE1) and CLK3 (off-target, PDB 6KHF).

We compare two optimization strategies using importance sampling: joint optimization that simultaneously maximizes the predicted binding affinity on CK2*α* while minimizing CLK3 affinity formulated as a dual-objective, versus the single optimization, which only maximizes CK2*α*. We generated 1, 000 ligands for each set using the protein pockets from PDB 3PE1^60^ and 6KHF^61^ after rotational alignment.

**Fig. 6:**
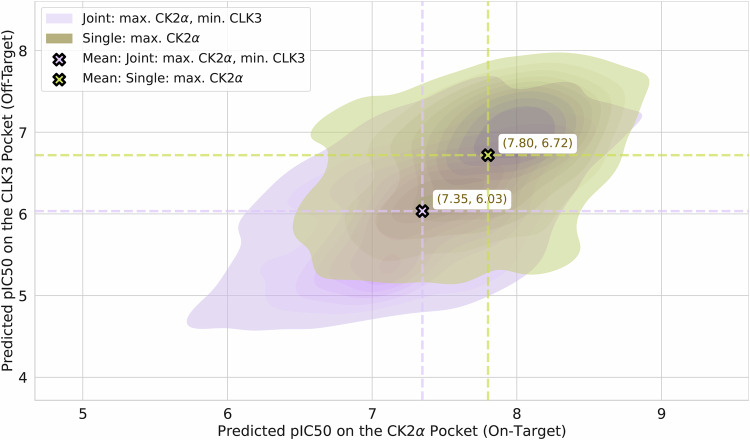
Evaluation of FLOWR.ROOT on affinity-guided selectivity optimization of CK2*α* and CLK3. Kernel density estimation plot comparing joint optimization (purple, maximizing CK2*α* while minimizing CLK3) versus single optimization (green, maximizing CK2*α* only) on predicted pIC_50_ values. Black X markers show mean values with dashed reference lines; the kernel density estimate visualises the per-strategy distribution. *n* = 1,000 ligands per optimization strategy per pocket (PDB 3PE1 for CK2*α*; PDB 6KHF for CLK3). Joint optimization achieves improved selectivity with lower off-target CLK3 activity (mean pIC_50_ = 6.03 vs. 6.72) while maintaining comparable on-target CK2*α* potency. Source data are provided as a Source Data file.

Our results show that joint optimization successfully achieves improved selectivity profiles compared to single-target optimization. The joint approach generated ligands with substantially lower off-target CLK3 activity (mean pIC_50_ = 6.03) compared to single optimization (mean pIC_50_ = 6.72), while maintaining comparable on-target CK2*α* potency as shown in the distribution plots in Fig. 6 with a mean pIC_50_ of 7.35 for the joint set on CK2*α*.

**Fig. 7:**
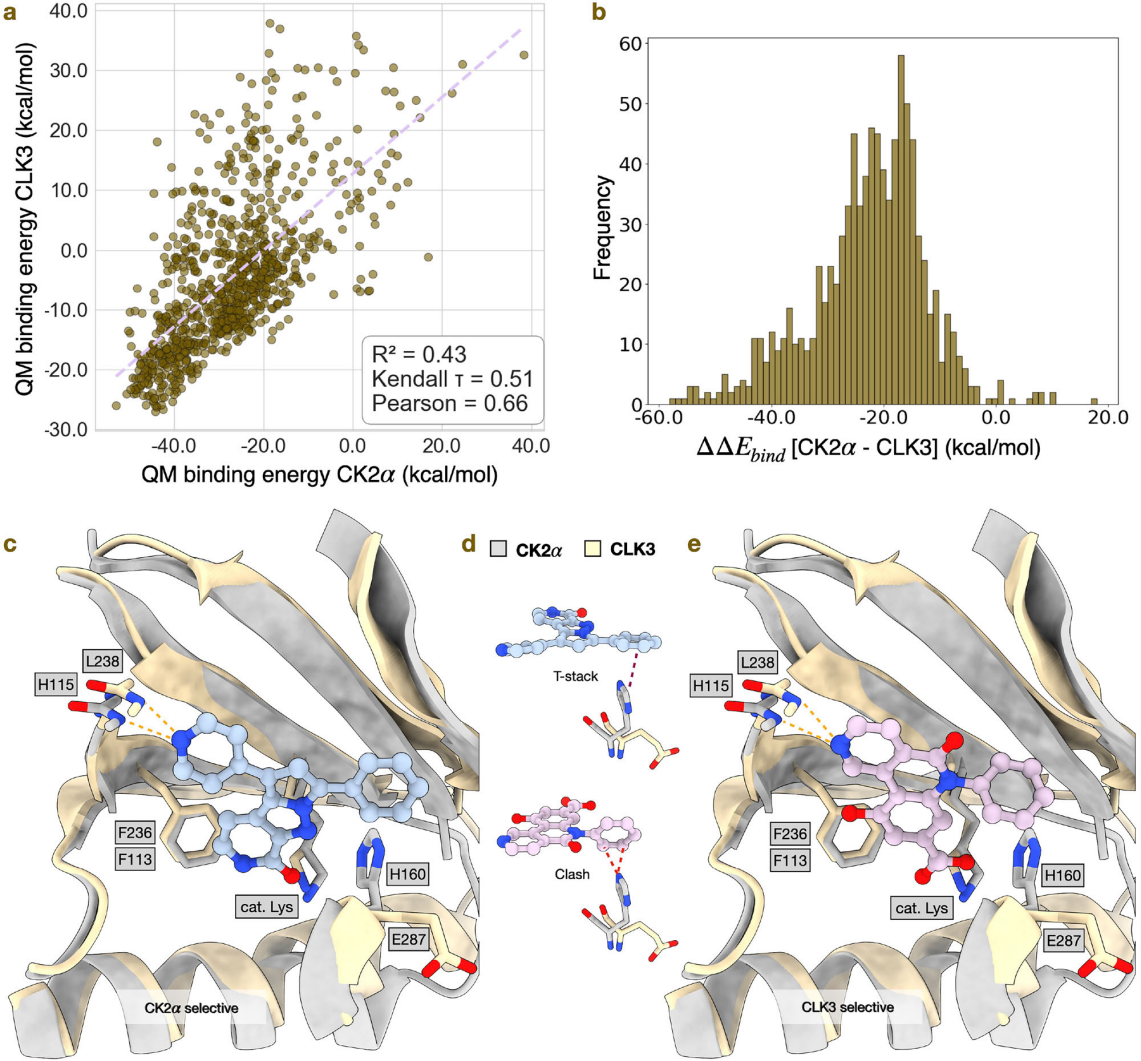
Quantum mechanical analysis of FLOWR.ROOT on selective ligand design on CK2*α* and CLK3. **a** Quantum-mechanical-calculated binding energy for CLK3 vs. CK2*α*. Ligands for which the CLK3 binding energy was above 40.0 kcal/mol were excluded ( ≈ 5.6%, structural clashes). The plot shows systematically lower binding energies for CK2*α*, indicating selectivity. **b** Distribution of the difference in binding energies for all retained ligands; the skewed distribution peaks at about −17.0 kcal/mol. **c** Example of a CK2*α*-selective ligand and its interaction patterns with the kinases' pockets. **d** Interactions of the two ligands with the residue H160. **e** Example of a CLK3-selective ligand and its interaction patterns with the kinases' pockets. Starting from *n* = 1000 jointly-optimized FLOWR.ROOT-generated ligands, *n* = 944 ligands enter the panel **a**/**b** statistics after the 40.0 kcal/mol exclusion. Each ligand contributes a single GFN2-xTB binding-energy evaluation per pocket. Source data are provided as a Source Data file.

We further benchmark our approach using an alternative protocol. To this end, we took the 1, 000 FLOWR.ROOT-generated ligands from the joint optimization and performed QM binding energy calculations. We excluded all complexes for which CLK3 binding energies were above 40.0 kcal/mol, as these resulted from structural clashes (*ca*. 5.6% of all complexes). Figure 7**a** shows a scatter plot of CK2*α* against CLK3 binding energies. The scatter shows a linearized correlation shifted towards lower CK2*α* binding energies, corroborating the success of the approach. Though other statistical metrics were calculated, we stress the calculated RMSE of 25.06 kcal/mol for this correlation. As RMSE measures deviation from the identity line, a large value implies selectivity towards one kinase, which, from the data, we conclude is CK2*α*. Further, we record several outliers from linearity, which result from the fact that FLOWR.ROOT tries to further penalize ligands in the CLK3 pocket. In this task, however, the most relevant metric is the relative binding energy of a given ligand towards the pockets of each kinase. Figure 7**b** shows the distribution of relative binding energies in these two kinases. The distribution, peaked at about  − 17.0 kcal/mol, is skewed, with a quick decay towards higher relative binding energies (less selective) and a slower decay on lower relative binding energies (more selective), further validating our approach.

It is particularly interesting to analyze ligands that minimize and maximize the relative binding energies between the two kinases. Figure 7**c**–**e** compare a CK2*α*-selective (ΔΔ*E*_*bind*_ = − 58.18 kcal/mol) against a CLK3 binder (ΔΔ*E*_*bind*_ = + 18.00 kcal/mol), indicating the mechanisms, with which FLOWR.ROOT tried to accomplish selectivity. Two major protein-ligand interactions were explored. On one hand, the interaction with the kinases’ hinge. In both cases, the hydrogen bond between the generated ligand and L238 of CLK3 shows a suboptimal angular orientation. Conversely, the CK2*α*-selective binder shows a quasi-optimal hydrogen bond orientation with CK2*α*’s H115, with the angle $H-{\widehat{N}}_{ar}-{C}_{ar}$ reaching a value of 114. 2^∘^. In the case of the CLK3-selective binder, the equivalent angle with CK2*α*’s main chain H of H115 goes up to 137. 9^∘^. Additional weakening of the hydrogen bond to CK2*α*’s hinge is achieved by increasing the $H-\widehat{N}$ distance, which goes from 2.77 Å in the CK2*α*-selective binder to 2.97 Å in the CLK3-selective one.

The second mechanism is related to interactions with H160. In previous work^62^, H160 was identified as a critical residue conferring sub-nM affinity of the inhibitor silmitasertib to CK2*α*. Here too, we observe that FLOWR.ROOT explores interactions with this residue to achieve selectivity. On the CK2*α*-selective binder, FLOWR.ROOT orients the ligand’s phenyl substituent to optimize a T-stack interaction. On the CLK3-selective binder, a similar substituent is rotated to yield a short, repulsive contact. This is possible because the side chain of E287, the residue in a similar position in CLK3, points in another direction.

We conclude that, in order to achieve selectivity between two similar kinases, FLOWR.ROOT exploited two previously identified elements of selectivity. This concerns the hinge region of kinases, where better binders show shorter hydrogen bond distances and more adequate angles. Simultaneously, FLOWR.ROOT also explored lipophilic interactions with the two kinases, optimizing a T-stack with a residue critical for the high potency of drugs currently in clinical trials. We stress that none of these interactions could have been passed to the model during training or in guiding selectivity.

### TYK2, ER*α*, and BACE1

As shown in previous sections, FLOWR.ROOT generates diverse chemical matter conditioned on a protein pocket. The generated molecules are chemically reasonable and are not heavily strained, unlike previous generative models. Additionally, the predicted affinity data reliably reproduces experimental distributions. In this section, we benchmark how the whole FLOWR.ROOT workflow behaves on the task of conditional ligand generation. To this end, we chose the TYK2 kinase, ER*α*, and BACE1 as targets from the Schrodinger FEP+ dataset.

Testing whether the FLOWR.ROOT-generated ligands are good binders that require effective metrics. In this case, we chose to benchmark the FLOWR.ROOT poses using QM binding energy analysis. QM calculations on protein-ligand complexes offer scoring functions; therefore, we aimed at finding a correlation between QM binding energies and the median of predicted binding affinities.

**Fig. 8:**
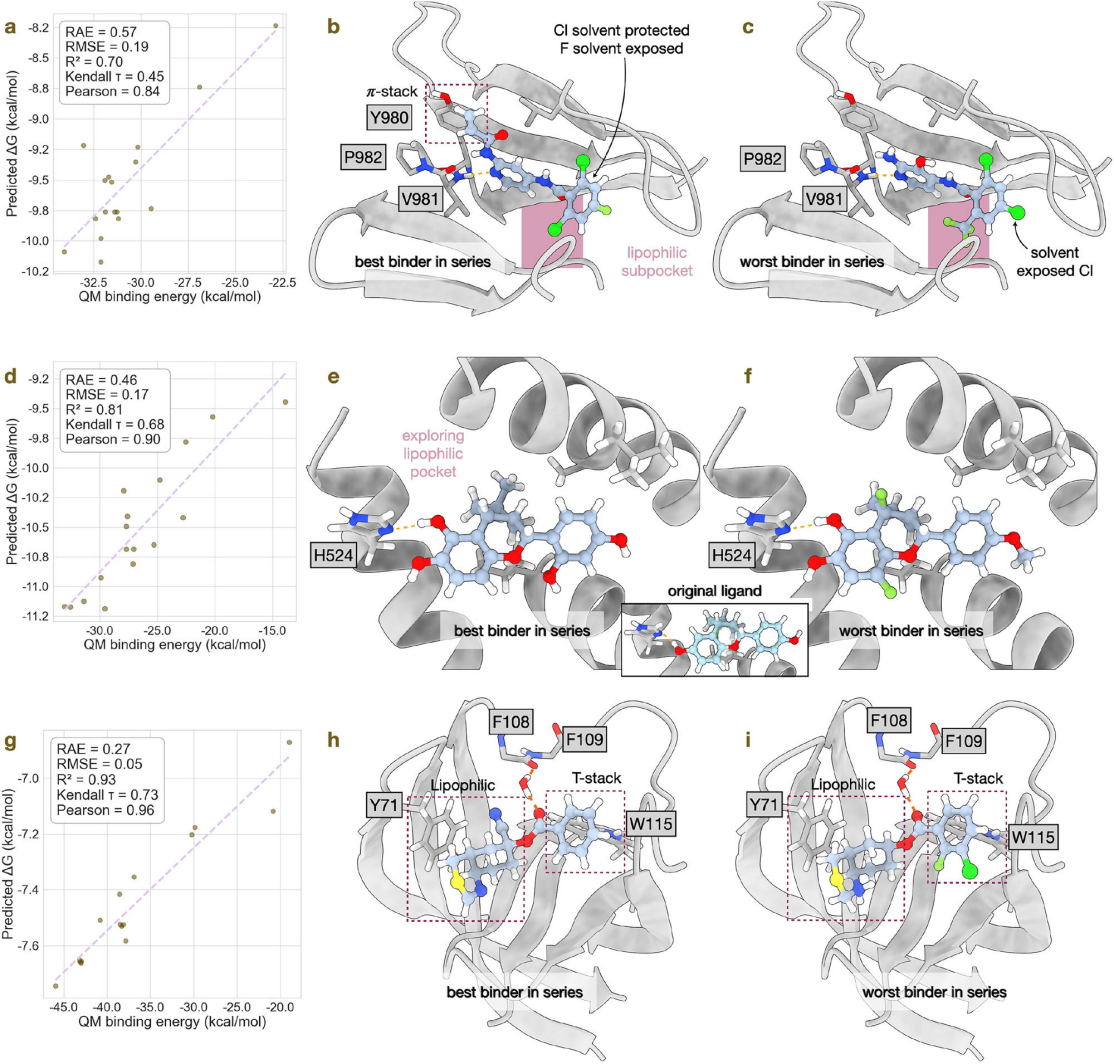
FLOWR.ROOT binding affinity validation using quantum mechanical calculations on TYK2, ER*α*, and BACE1. Benchmark cases of the TYK2 kinase (**a**–**c**), ER*α* (**d**–**f**), and BACE1 (**g**–**i**). **a** correlation plot between FLOWR.ROOT predicted affinities and the quantum mechanical binding energies for TYK2. **b** example of the best binder in the series and its interactions with the pocket. **c** example of the worst binder in the series and its interactions with the pocket. **d** correlation plot between FLOWR.ROOT predicted affinities and the quantum mechanical binding energies for ER*α*. **e** example of the best binder in the series and its interactions with the pocket. **f** example, the worst binder in the series and its interactions with the pocket. **g** correlation plot between FLOWR.ROOT predicted affinities and the respective quantum mechanical binding energies. **h** example of the best binder in the series and its interactions with the pocket. **i** example of the worst binder in the series and its interactions with the pocket. Each correlation panel (**a**, **d**, **g**) uses *n* = 17 FLOWR.ROOT-generated ligands per target. Each ligand contributes one GFN2-xTB binding-energy evaluation and one FLOWR.ROOT affinity prediction; reported R^2^, Pearson, Kendall *τ*, RMSE, and RAE are point estimates over the full per-target ligand set. Reference complexes: BACE1 PDB 4ZSP, TYK2 PDB 4GIH, ER*α* PDB 2Q70. Source data are provided as a Source Data file.

We first look at the example of the TYK2 kinase (Fig. 8**a-c**). We find a good correlation between the QM-calculated binding energies and the FLOWR.ROOT-predicted median affinities, with an R^2^ of 0.70, a Pearson correlation coefficient of 0.84, a Kendall *τ* of 0.45, RMSE of 0.19, and RAE of 0.57. From a chemical viewpoint, it is more interesting to compare the structures of the best and worst FLOWR.ROOT-predicted ligands. We see that, despite some common features, the model generalizes effectively the hinge-binding motif and the solvent-exposed residue of the ligand. Importantly, all ligands retained the hydrogen bond acceptor to V981, a critical hinge residue already in the original ligand structure. Binding to a kinase’s hinge is very important in kinase inhibition, kept by FLOWR.ROOT-generated chemical matter. Interestingly, no ligand improved the hydrogen-bond geometry with P982, despite some variations in the chemotypes exhibited by the hinge-binding heads. The major differences take place in how specific interactions are exploited. Particularly critical for the best binder in this series is the *π*-stack interaction established with Y980. But also the fact that this ligand protects the least lipophilic halogens from solvent exposure. We also calculated ligand deformation energies, which for this series lie between 19.32 kcal/mol and 44.29 kcal/mol.

A similar correlation was obtained for ER*α* (Fig. 8**d**–**f**), however, in this case, all the statistical metrics improved: The R^2^ is 0.81, the Pearson correlation coefficient is 0.90, the Kendall *τ* is 0.68, the RMSE is 0.17, and the RAE is 0.46. Some variability is expected between systems, and in the case of ER*α*, we observe fewer outliers than in the TYK2 example. In the case of ER*α*, we observed that FLOWR.ROOT retained most of the ligand’s structure, improving mostly the interactions with the residue H524. In the structure of the original ligand, there is a poorly-oriented hydrogen bond with H524, which is effectively corrected even in the case of the worst binder: the FLOWR.ROOT-generated ligands exhibited better geometries for this hydrogen bond. Similar to the case of the TYK2 kinase, we calculated ligand deformation energies, which were between 24.36 kcal/mol and 38.30 kcal/mol.

Finally, we also analyzed a benchmark on BACE1 (Fig. 8**g-i**), where we obtained the best correlation of this series between FLOWR.ROOT-predicted affinities and the QM calculated ones. This is seen in the lowest RAE and RMSE coupled with the highest R^2^, Pearson correlation coefficient, and Kendall *τ* of this series of benchmarks. We see that in all cases the ligands retain a water-mediated hydrogen bond with F108, which results in high affinities overall. The major differences are in the nature of the nitrogen-containing group near Y71, which seems to favor more lipophilic groups, but also the substituents in the aromatic ring, T-stacking the residue W115. We note that the T-stack interaction is favored by electron-rich phenyl rings, which is not the case for the worst binder in the series. This seems then to be one of the major effects controlling affinity to BACE1.

## Discussion

The integration of pocket-aware generation, multi-endpoint affinity prediction, and efficient domain adaptation within a single architecture addresses a central challenge in computational drug design: bridging the gap between generating geometrically realistic ligands and reliably estimating their binding properties, while remaining adaptable to project-specific structure–activity landscapes. FLOWR.ROOT realizes this integration through an SE(3)-equivariant flow matching framework that couples structural prediction with dedicated affinity and confidence heads, enabling property-guided molecule design through inference-time steering. By supporting de novo design, interaction-guided generation, and fragment-based elaboration within a unified backbone, the framework spans applications from early-stage hit identification to late-stage lead optimization.

Across established benchmarks, the model achieves leading performance in both pocket-conditional ligand generation and affinity prediction, consistently outperforming recent diffusion- and flow-based approaches while producing geometrically realistic, low-strain structures. The joint affinity head matches or exceeds the accuracy of computationally expensive physics-based methods and clearly surpasses ML-based scoring functions, at orders of magnitude greater throughput. Importantly, targeted parameter-efficient finetuning enhances prediction reliability on project-specific datasets substantially, demonstrating practical utility in real-world medicinal chemistry workflows.

Quantum mechanical validation studies on TYK2 kinase, ER*α*, and BACE1 provide evidence that FLOWR.ROOT generates chemically meaningful ligands that systematically exploit key structural motifs—hinge-binding interactions, hydrogen bond geometries, and aromatic stacking—indicating that the model has internalized fundamental principles of protein-ligand recognition. The kinase selectivity study between CK2*α* and CLK3 further illustrates that affinity-guided generation can surface selectivity mechanisms. Together, these results support the broader vision of continuously evolving frameworks that refine their understanding through sustained interaction with project-specific data, offering a foundational yet adaptable architecture for structure-based drug design.

Nevertheless, important limitations remain. The reliance on public datasets, despite extensive curation, introduces biases from noisy affinity measurements, overrepresented protein families, and uneven chemical diversity. While higher-fidelity datasets partially mitigate these issues, their limited scale constrains model calibration, particularly for underexplored targets. Project-specific adaptation, though powerful, requires carefully chosen objectives and sufficient assay data; otherwise, models risk overfitting to narrow distributions. Additionally, FLOWR.ROOT requires the binding pocket to be known and preferably in a holo conformation, leaving the challenge of modeling protein pocket flexibility to future work. Finally, the predictions of FLOWR.ROOT remains in silico approximations that cannot substitute for experimental validation.

Looking forward, several directions appear promising. Expanding project-level adaptation to include reinforcement learning or active learning frameworks with both physics-based and experimental feedback may enable continuous refinement during discovery campaigns. Further, enhancing synthetic accessibility estimation and coupling to reaction-based generative models would improve downstream feasibility, ensuring that generated molecules are not only potent but also synthetically accessible.

## Methods

This section outlines the methodology employed in our framework, FLOWR.ROOT, for generative structure-aware ligand modeling and binding affinity prediction. We provide a detailed description of the architecture and key components, including framework details about the three-stage training process, the generative modes available for ligand design, and the affinity prediction capabilities. We also discuss the strategies for confidence estimation, inference-time scaling via importance sampling, and domain adaptation as the main application in practical drug discovery workflows.

### FLOWR.ROOT

FLOWR.ROOT is built on the recently proposed FLOWR architecture, an *SE*(3)-equivariant flow matching model that learns a mixed continuous/discrete transport map. This map transforms a prior distribution (for example, random noise or fragment anchors for coordinates) to the target ligand distribution within a given protein pocket^17^. The model consists of two main components: a pocket encoder and a ligand decoder.

Protein pockets are extracted from holo structures by cutting residues within a defined radius around the binding site. We use a 7 Å cutoff radius, which represents a practical compromise between computational tractability and interaction coverage. This cutoff fully encompasses hydrogen bonds directly relevant to ligand binding (which decay rapidly beyond 5.5–6 Å), captures vibrational entropy contributions from direct protein-ligand interactions, and includes conformational entropy changes in the binding pocket. While long-range electrostatic effects are system-dependent (primarily affecting charged ligands), the 7 Å cutoff provides sufficient coverage for neutral ligands, and partial cancellation occurs for charged species. Water-mediated interactions are treated implicitly, consistent with many previous ML approaches. The cutoff is a configurable parameter that can be adjusted at both training and inference time.

These pockets are then encoded using an equivariant self-attention module followed by equivariant feed-forward layers, generating a set of invariant and equivariant protein features^17^. The ligand decoder processes noisy ligand coordinates, partial charges, atom types, bond orders, and hybridization states through equivariant self-attention modules, which capture intra-ligand dependencies. A cross-attention layer then integrates contextual information from the pocket features to the hidden ligand features. The pocket encoder processes features on a full-atom basis through *SE*(3)-equivariant self-attention layers using invariant residue embeddings, equivariant coordinate features, and pairwise fully-connected distance information between all pocket atoms. Via fully-connected, equivariant cross-attention, the ligand decoder interacts with the invariant and equivariant pocket encodings for ligand generation. For additional details, we refer to Cremer et al.^17^.

In FLOWR.ROOT, the ligand decoder has three output heads: (1) a structure head that predicts atomic coordinates, atom types, bond orders, charges, and hybridization states; (2) an affinity head comprising four separate potency and binding affinity heads—specifically, for IC_50_, p*K*_i_, p*K*_d_, and pEC_50_ prediction—and (3) a confidence head that provides uncertainty estimation based on pLDDT^14^. Importantly, these heads serve distinct yet complementary purposes: the structure head generates geometrically valid ligand conformations within the protein pocket, while the affinity head provides potency and binding affinity estimates that enable (1) in-distribution evaluation of generated structures, (2) inference-time steering toward higher-affinity compounds via importance sampling, and (3) domain adaptation of both structure and affinity prediction through joint finetuning. This joint training paradigm thus facilitates coherent, in-distribution property-guided generation without requiring external scoring functions-avoiding distribution mismatch and additional computational overhead-while supporting effective domain adaptation.

Importantly, FLOWR.ROOT builds upon the multi-task training framework of FLOWR^17^, yet extends it substantially to support four generative modes within a single backbone: (1) de novo and (2) interaction-conditional generation for preserving or enforcing pharmacophoric patterns, (3) scaffold hopping and elaboration, and (4) fragment growing and replacement. Notably, with the latter two FLOWR.ROOT supports specifying which atoms to replace while preserving the remainder of the molecule. To enforce locality in these partial modifications, we introduce a flexible prior placement strategy that shifts the generative prior from the zero center-of-mass to the local replacement site. This multi-mode capability enables flexible application across hit identification and hit-to-lead and lead optimization with targeted structural modifications.

The core challenge in protein-ligand modeling, however, lies not just in generation, but in accurately predicting protein-ligand interactions and potency. The sparsity of experimental binding data-where most ligand-protein combinations remain unexplored-poses a substantial obstacle to improving these predictions. While broad-coverage datasets encompass a wide spectrum of protein targets, they are intrinsically sparse, with few ligands tested systematically across different biological contexts. This imbalance leads to biased datasets, which explains why 3D machine and deep learning models-often relying only on protein or ligand descriptors and ignoring interaction patterns-can perform surprisingly well on affinity prediction benchmarks like PDBbind^35^. Real-world drug discovery requires understanding nuanced SAR across hundreds or even thousands of related molecules within the same binding site-a richness uniquely captured by dense bioactivity landscapes. These landscapes encode redundancy and critical phenomena like activity cliffs, where small structural changes can lead to substantial shifts in bioactivity due to interaction changes. This motivates the multi-stage training approach of FLOWR.ROOT: starting with sparse, lower-fidelity data to learn broad 3D chemistry and binding principles, and progressively refining the model with denser, higher-fidelity, and project-specific datasets that capture subtle SAR patterns essential for effective hit expansion to lead optimization. This multi-stage pipeline is intended to closely mirror the practical needs of early drug discovery campaigns, addressing the limitations imposed by data sparsity in current 3D affinity prediction models. The FLOWR.ROOT framework consists of three stages:

We position FLOWR.ROOT as a foundation model in the sense formalized by Bommasani et al.^63^, who define a foundation model as a model that is trained on broad data at scale, meant to be adapted (for example, LoRA-fine-tuned) to a range of downstream tasks. Critically, foundation models are described as unfinished, intermediary assets requiring adaptation^63–65^. In this work, the foundation model property of FLOWR.ROOT refers specifically to its broad multi-stage pre-training on diverse chemical and structural data at scale, combined with its adaptability to specific downstream drug discovery tasks via parameter-efficient fine-tuning (LoRA;^47^). We note that zero-shot generalization, especially across arbitrary bioactivity landscapes, is not implied; rather, we expect the model to provide transferable representations that enable rapid, data-efficient domain adaptation.

#### Stage 1: Large-scale pre-training

This stage builds a general-purpose generative prior across chemical and structural distributions, with a broad bioactivity landscape, albeit with varying quality and predictive relevance. The model is trained on a mixture of:

- **Small molecules in vacuum** (~1.5B datapoints from ZINC3D, PubChem3D, Enamine, OMol25) to ensure broad chemical coverage.
- **Protein-ligand complexes with and without affinity labels** (~2.5M datapoints from Plinder, BindingMoad, BindingNet, SAIR, KIBA-3D, Kinodata-3D, OMol25), which capture protein-ligand structural diversity, in parts annotated with affinity data. This dataset is split into lower- and higher-fidelity categories. We classify complexes with computational origin (that is, BindingNet, SAIR, KIBA-3D, Kinodata-3D) as lower-fidelity and those stemming from co-crystal data (that is, Plinder, BindingMoad) as higher-fidelity, with the exception of the high-confidence subset of BindingNet, which is treated as higher-fidelity.

#### Stage 2: High-fidelity finetuning

Pre-trained weights are adapted to thoroughly curated, drug-like, high-quality affinity datasets. In this work, we use SPINDR and HIQBIND (~30k complexes) as datasets for multi-target finetuning. However, this stage can be used to finetune FLOWR.ROOT on diverse, high-quality in-house data, if available, which teaches the model the fine details of protein-ligand interactions and binding affinity.

#### Stage 3: Project-specific domain adaptation

This ensures that FLOWR.ROOT can bridge the gap between large-scale, noisy public data and small-scale, high-quality, dense in-assay data, producing distributions that align with specific discovery campaigns. Different domain adaptation strategies are used to fine-tune and steer the pre-trained FLOWR.ROOT model:

- **Inference-time scaling and property steering:** Importance sampling and path reweighting guide the model toward desired properties such as affinity, ADME/T, or synthetic accessibility.
- **Direct preference alignment:** The model is finetuned with project-specific preferences, such as avoiding known liability motifs or penalizing activity cliffs (future work).
- **Finetuning:** Parameter-efficient finetuning, namely Low-Rank Adapation (LoRA)^47^, helps avoid catastrophic forgetting while aligning the model to project-specific structure-activity modes.

### Training and Inference

Following the methodology of Cremer et al.^17^, we adopted the training and parameterization scheme from FLOWR.MULTI. Specifically, we employed a 4-layer pocket encoder with ${d}_{\,{{\rm{inv}}}}^{{{\rm{enc}}}}=256$ and a 12-layer ligand decoder with ${d}_{\,{{\rm{inv}}}}^{{{\rm{dec}}}}=384$. The equivariant feature dimension was set to *d*_equi_ = 128 for both the pocket encoder and ligand decoder. For latent attention, we utilized a latent size of 64 with 32 attention heads. Overall, FLOWR.ROOT has approximately 33 million trainable parameters. Throughout this work, the model has been trained on one NVIDIA H100 node comprising eight H100 GPUs.

To further enhance model stability and generalization, we incorporated residual coordinate and subsequent edge updates using Gaussian radial basis expansions^10^, applied to the layer-wise latent message-passing outputs of the FLOWR model backbone^17^. Additionally, we included distance and cross-product computations between latent equivariant node features within the message-passing layers, which we found to improve model performance. Specifically, in the pairwise interactions between nodes, given the equivariant queries ${q}_{{{\rm{equi}}}}\in {{\mathbb{R}}}^{B\times {N}_{q}\times 3\times {d}_{{{\rm{equi}}}}}$ and keys ${k}_{{{\rm{equi}}}}\in {{\mathbb{R}}}^{B\times {N}_{kv}\times 3\times {d}_{{{\rm{equi}}}}}$, we define the pairwise difference tensor as

1 $${{\bf{D}}}={{\bf{Q}}}[: \!,: \!,{{\bf{1}}},:]-{{\bf{K}}}[: \!,{{\bf{1}}},: \!,:],$$

and compute the distance features as

2 $${{\bf{S}}}={\left|{{\bf{D}}}\right|}_{2}\in {{\mathbb{R}}}^{B\times {N}_{q}\times {N}_{k}\times d}.$$

The pairwise cross product tensor is defined as

3 $${{\bf{C}}}={{\bf{Q}}}[: \!,: \!,{{\bf{1}}},:]\times {{\bf{K}}}[: \!,{{\bf{1}}},: \!,:],$$

where  × denotes the vector cross product over the 3-vector dimension, resulting in

4 $${{\bf{C}}}\in {{\mathbb{R}}}^{B\times {N}_{q}\times {N}_{k}\times 3\times d}.$$

Both **S** and **C** are stacked to the existing pairwise message tensor as additional features.

The model structure output comprises predictions for atomic coordinates, atom types, charge, hybridization, bond types, bond lengths and bond angles. The overall loss function reads:

5 $${{{\mathcal{L}}}}_{{{\rm{total}}}}={\lambda }_{{{\rm{c}}}}\underbrace{{{\rm{MSE}}}({{{\bf{X}}}}_{{{\rm{pred}}}},{{{\bf{X}}}}_{{{\rm{true}}}})}_{{{\rm{Coordinate}}}\,{{\rm{loss}}}}\,+{\lambda }_{{{\rm{t}}}}\underbrace{{{\rm{CE}}}({{{\bf{T}}}}_{{{\rm{pred}}}},{{{\bf{T}}}}_{{{\rm{true}}}})}_{{{\rm{Atom}}}\,{{\rm{type}}}\,{{\rm{loss}}}}\,+{\lambda }_{{{\rm{ch}}}}\underbrace{{{\rm{CE}}}({{{\bf{Q}}}}_{{{\rm{pred}}}},{{{\bf{Q}}}}_{{{\rm{true}}}})}_{{{\rm{Charge}}}\,{{\rm{loss}}}}\,+$$

6 $${\lambda }_{{{\rm{h}}}}\underbrace{{{\rm{CE}}}({{{\bf{H}}}}_{{{\rm{pred}}}},{{{\bf{H}}}}_{{{\rm{true}}}})}_{{{\rm{Hybridization}}}\,{{\rm{loss}}}}\,+{\lambda }_{{{\rm{b}}}}\underbrace{{{\rm{CE}}}({{{\bf{B}}}}_{{{\rm{pred}}}},{{{\bf{B}}}}_{{{\rm{true}}}})}_{{{\rm{Bond}}}\,{{\rm{type}}}\,{{\rm{loss}}}}+{\lambda }_{{{\rm{bl}}}}\underbrace{{{{\mathcal{L}}}}_{{{\rm{bondlength}}}}}_{{{\rm{Bond}}}\,{{\rm{length}}}\,{{\rm{loss}}}}+{\lambda }_{{{\rm{ba}}}}\underbrace{{{{\mathcal{L}}}}_{{{\rm{bondangle}}}}}_{{{\rm{Bond}}}\,{{\rm{angle}}}\,{{\rm{loss}}}}$$

with *λ*_*i*_ denoting the respective loss weighting. To encourage geometrically accurate bond lengths, we compute the mean squared error between predicted and ground truth distances for all bonded atom pairs:

7 $${{{\mathcal{L}}}}_{{{\rm{bond}}}\; {{\rm{length}}}}=\frac{1}{| {{\mathcal{B}}}| }\mathop{\sum }\limits_{(i,j)\in {{\mathcal{B}}}}{\left(\parallel \! {{{\bf{x}}}}_{i}^{{{\rm{pred}}}}-{{{\bf{x}}}}_{j}^{{{\rm{pred}}}}{\parallel }_{2}-\parallel \! {{{\bf{x}}}}_{i}^{{{\rm{true}}}}-{{{\bf{x}}}}_{j}^{{{\rm{true}}}}{\parallel }_{2}\right)}^{2}$$

where ${{\mathcal{B}}}$ denotes the set of bonded atom pairs extracted from the bond adjacency matrix, considering only the upper triangle to avoid redundancy. To preserve local molecular geometry, we penalize deviations in bond angles for all valid triplets (*i*, *j*, *k*) where atom *j* is bonded to both atoms *i* and *k*. For each triplet, the bond angle *θ*_*ijk*_ is computed as:

8 $${\theta }_{ijk}=\arccos \left(\frac{({{{\bf{x}}}}_{i}-{{{\bf{x}}}}_{j})\cdot ({{{\bf{x}}}}_{k}-{{{\bf{x}}}}_{j})}{\parallel \! {{{\bf{x}}}}_{i}-{{{\bf{x}}}}_{j}{\parallel }_{2}\cdot \parallel \! {{{\bf{x}}}}_{k}-{{{\bf{x}}}}_{j}{\parallel }_{2}}\right)$$

The bond angle loss is then defined using the Huber loss for robustness:

9 $${{{\mathcal{L}}}}_{{{\rm{bond}}}\; {{\rm{angle}}}}=\frac{1}{| {{\mathcal{A}}}| }\mathop{\sum }\limits_{(i,j,k)\in {{\mathcal{A}}}}{{{\rm{Huber}}}}_{\delta }\left({\theta }_{ijk}^{{{\rm{pred}}}}-{\theta }_{ijk}^{{{\rm{true}}}}\right)$$

where ${{\mathcal{A}}}$ is the set of valid angle triplets, and the Huber loss with threshold *δ* is defined as:

10 $${{{\rm{Huber}}}}_{\delta }(x)=\left\{\begin{array}{ll}\frac{1}{2}{x}^{2} \hfill & \,{{\rm{if}}}\,| x| \le \delta \\ \delta \left(| x| -\frac{1}{2}\delta \right) & \,{{\rm{otherwise}}}\end{array}\right.$$

We use *δ* = 0.5 radians to balance sensitivity to small deviations while remaining robust to outliers.

FLOWR.ROOT jointly models both continuous (coordinates) and discrete (atom types, bond orders, charges, hybridizations) molecular features. For coordinates, we employ continuous flow matching^66^, while discrete flow models^15^ are used for categorical variables. The model is trained to recover the original ligand *l*_1_ from a noisy ligand *l*_*t*_ by learning the conditional distribution ${p}_{1| t}^{\theta }({l}_{1}| {l}_{t},t;{{\mathcal{P}}})$, minimizing mean squared error for coordinates and cross-entropy loss for categorical features. Given a pocket structure ${{\mathcal{P}}}$, new ligands are generated by iteratively refining an initial noisy ligand *l*_0_ ~ *p*_noise_. The generative process follows a learned vector field ${v}_{t}^{\theta }$ for continuous features and a discrete integration scheme for categorical attributes^15,17^.

#### Anisotropic and reference-ligand-based prior strategies

Standard flow matching employs an isotropic Gaussian ${{\mathcal{N}}}({{\bf{0}}},{{\bf{I}}})$ as the source distribution *p*_0_. In partial generation modes—scaffold hopping, scaffold elaboration, linker inpainting, core growing, and fragment growing—a fixed molecular substructure provides geometric context that constrains where new atoms should be placed. An isotropic prior distributes probability mass without directional bias, forcing the learned velocity field ${v}_{t}^{\theta }$ to perform long-range, direction-dependent transport to reach the target region. A distinguishing property of flow matching, in contrast to diffusion models where the terminal distribution is determined by the noise schedule, is that *p*_0_ need not be a standard Gaussian: any distribution from which efficient sampling is possible suffices^66^. The velocity field is trained to transport whatever *p*_0_ is supplied toward *p*_1_, enabling per-sample prior selection and even mixed isotropic and anisotropic batches within a single training step. FLOWR.ROOT exploits this flexibility by constructing task-specific anisotropic Gaussian priors whose covariance encodes the spatial extent and directionality of the generation context.

Two complementary covariance constructions are employed. The shape-based covariance captures the spatial extent of a set of reference atom coordinates. Given a set of *N* reference atom coordinates ${\{{{{\bf{r}}}}_{i}\}}_{i=1}^{N}$ (variable atoms or the full molecule, depending on the generation mode), the centered coordinate matrix is

11 $$\bar{{{\bf{X}}}}={{\bf{X}}}-\frac{1}{N}{{\bf{1}}}{{{\bf{1}}}}^{\top }{{\bf{X}}},\,{{\bf{X}}}\in {{\mathbb{R}}}^{N\times 3},$$

and the sample covariance is

12 $${{\bf{C}}}=\frac{1}{N-1}\,{\bar{{{\bf{X}}}}}^{\top }\bar{{{\bf{X}}}}.$$

Eigendecomposing **C** = **V** diag(*λ*_1_, *λ*_2_, *λ*_3_) **V**^⊤^ with *λ*_1_≥*λ*_2_≥*λ*_3_≥0, the eigenvalues are first normalized to satisfy ${\sum }_{k}{\widetilde{\lambda }}_{k}=3$:

13 $${\widetilde{\lambda }}_{k}={\lambda }_{k}\cdot \frac{3}{\mathop{\sum }\limits_{j}{\lambda }_{j}},$$

then clamped to $[{\lambda }_{\min },{\lambda }_{\max }]$ and re-normalized to restore the trace constraint. The shape-based covariance is

14 $${{{\mathbf{\Sigma }}}}_{{{\rm{shape}}}}={{\bf{V}}}\,{{\rm{diag}}}({\lambda }_{1}^{*},{\lambda }_{2}^{*},{\lambda }_{3}^{*})\,{{{\bf{V}}}}^{\top }.$$

This construction is applied in scaffold hopping and core growing (using the variable-atom coordinates as reference), as well as in scaffold elaboration and linker inpainting (using the full-molecule coordinates to capture the spatial extent of the entire ligand).

The directional covariance encodes a preferred growth direction from a fixed fragment toward a target region. Given the center of mass of the fixed fragment **c**_src_ and a target center **c**_tgt_ (the prior center or the center of mass of the variable atoms), the unit direction is

15 $${{\bf{d}}}=\frac{{{{\bf{c}}}}_{{{\rm{tgt}}}}-{{{\bf{c}}}}_{{{\rm{src}}}}}{\parallel \!{{{\bf{c}}}}_{{{\rm{tgt}}}}-{{{\bf{c}}}}_{{{\rm{src}}}}{\parallel }_{2}}.$$

An orthonormal basis {**v**_2_, **v**_3_, **d**} is obtained via Gram–Schmidt orthogonalization, yielding a rotation matrix **R**_dir_ = [**v**_2_∣**v**_3_∣**d**] ∈ *SO*(3). With an elongation factor *α* > 1 (default *α* = 2) and the trace normalization constraint ${{\rm{tr}}}({{\mathbf{\Sigma }}})=3$, the eigenvalues become

16 $${\lambda }_{\perp }=\frac{3}{2+\alpha },\,{\lambda }_{\parallel }=\frac{3\alpha }{2+\alpha },$$

and the directional covariance reads

17 $${{{\mathbf{\Sigma }}}}_{{{\rm{dir}}}}={{{\bf{R}}}}_{{{\rm{dir}}}}\,{{\rm{diag}}}({\lambda }_{\perp },{\lambda }_{\perp },{\lambda }_{\parallel })\,{{{\bf{R}}}}_{{{\rm{dir}}}}^{\top }.$$

This construction is used for fragment growing, where the direction points from the fixed fragment toward the prior center, and composes with the reference-ligand center-of-mass placement described below.

Both covariance types satisfy the trace invariant ${{\rm{tr}}}({{\mathbf{\Sigma }}})=3$, ensuring ${\mathbb{E}}\left[\parallel \!{{{\bf{x}}}}_{0}{\parallel }^{2}\right]=3$ for ${{{\bf{x}}}}_{0} \sim {{\mathcal{N}}}({{\bf{0}}},{{\mathbf{\Sigma }}})$, the same second moment as the isotropic prior. This guarantees consistent velocity field magnitudes across prior types and enables stable mixed-batch training in which isotropic and anisotropic samples coexist. Since both **Σ**_shape_ and **Σ**_dir_ are derived from reference atom coordinates, they transform equivariantly under rigid-body transformations of the protein–ligand complex, preserving the *SE*(3)-equivariance of the model. Sampling from ${{\mathcal{N}}}({{\bf{0}}},{{\mathbf{\Sigma }}})$ is performed via the Cholesky decomposition **Σ** = **LL**^⊤^, yielding **x**_0_ = **z** **L**^⊤^ with ${{\bf{z}}} \sim {{\mathcal{N}}}({{\bf{0}}},{{\bf{I}}})$.

Orthogonal to the covariance construction, the prior point cloud can be spatially relocated to improve locality. For global inpainting modes, the prior cloud is shifted to the center of mass of the to-be-generated atoms in the reference ligand. During training, Gaussian noise with standard deviation *σ*_noise_ is added to this placement for robustness. Covariance controls the shape of the prior while placement controls its location; the two compose independently. For substructure and fragment inpainting, the prior cloud is shifted to the center of mass of the to-be-replaced fragment in the reference ligand, and an isotropic ${{\mathcal{N}}}({{\bf{0}}},{{\bf{I}}})$ prior suffices since the spatial placement itself provides sufficient locality.

#### Potency and binding affinity prediction

In drug discovery, a variety of parameters, including IC_50_, *K*_i_, *K*_d_, and EC_50_, are reported as potency and binding affinity measures. These parameters differ in their definitions and experimental setups, which complicates their direct comparison. For example, *K*_d_ is an equilibrium constant that directly measures the strength with which a ligand binds to its target protein. On the other hand, a *K*_i_ is an equilibrium constant indicating how well a given inhibitor inhibits the binding of a natural substrate. In other words, while *K*_d_ measures the stickiness of a molecule to a target, *K*_i_ measures how well the inhibitor blocks the enzyme from its natural substrate. Though these values often correlate, they are not always interchangeable. Likewise, EC_50_ and IC_50_ are used for biological assays. Though they correlate with *K*_d_ and *K*_i_, their interpretation depends on assay conditions. For better readability, for the remainder of this work we refer to potency and binding affinity prediction as affinity prediction.

Therefore, in contrast to prior work^36,37^, we predict each potency measure separately, aligning better with the complexity of drug-target data. Specifically, FLOWR.ROOT employs separate prediction heads for each affinity type (pIC_50_, p*K*_i_, p*K*_d_, pEC_50_), with each head trained separately on its corresponding data type. This design explicitly avoids treating heterogeneous affinity labels as interchangeable. Given invariant and equivariant ligand features ${{{\bf{h}}}}_{i}^{{{\rm{inv}}}},{{{\bf{h}}}}_{i}^{{{\rm{equi}}}}$, pocket features ${{{\bf{p}}}}_{j}^{{{\rm{inv}}}},{{{\bf{p}}}}_{j}^{{{\rm{equi}}}}$, and ligand-pocket interaction features **e**_*ij*_ extracted from the ligand decoder’s latent message-passing module, the affinity head computes:

18 $${{{\bf{f}}}}_{i}^{{{\rm{lig}}}}={{\rm{Gate}}}({{{\bf{h}}}}_{i}^{{{\rm{inv}}}},{{{\bf{h}}}}_{i}^{{{\rm{equi}}}})$$

19 $${{{\bf{z}}}}^{{{\rm{lig}}}}={{{\rm{MLP}}}}_{{{\rm{lig}}}}\left(\left[\frac{1}{C}\mathop{\sum }\limits_{i}{{{\bf{f}}}}_{i}^{{{\rm{lig}}}},\,\frac{1}{| {{\mathcal{L}}}| }\mathop{\sum }\limits_{i}{{{\bf{f}}}}_{i}^{{{\rm{lig}}}}\right]\right)$$

20 $${{{\bf{f}}}}_{j}^{{{\rm{pocket}}}}={{\rm{Gate}}}({{{\bf{p}}}}_{j}^{{{\rm{inv}}}},{{{\bf{p}}}}_{j}^{{{\rm{equi}}}})$$

21 $${{{\bf{z}}}}^{{{\rm{pocket}}}}={{{\rm{MLP}}}}_{{{\rm{pocket}}}}\left(\frac{1}{| {{\mathcal{P}}}| }\mathop{\sum }\limits_{j}{{{\bf{f}}}}_{j}^{{{\rm{pocket}}}}\right)$$

22 $${{{\bf{z}}}}^{{{\rm{int}}}}={{{\rm{MLP}}}}_{{{\rm{int}}}}\left(\frac{{\sum }_{i,j}{{{\bf{e}}}}_{ij}{m}_{i}{m}_{j}}{{\sum }_{i,j}{m}_{i}{m}_{j}}\right)$$

where Gate is a Gated equivariant block^67^ to combine equivariant and invariant feature tensors, and MLP is a multi-layer perceptron with two linear layers and SiLU activation. The input to MLP_lig_ is the concatenation of normalized invariant features, where *C* = 100, and $| {{\mathcal{L}}}|$ denotes the number of atoms in the ligand. The concatenated feature tensor **z** = [**z**^lig^, **z**^pocket^, **z**^int^] is passed to task-specific heads defined as

23 $${y}_{{{\rm{affinity}}}}={{\rm{ReLU}}}({{{\rm{MLP}}}}_{{{\rm{affinity}}}}({{\bf{z}}})).$$

The affinity prediction loss is computed using the mean Huber loss between predicted and true affinity values in log units (pIC_50_, p*K*_d_, p*K*_i_, pEC_50_) through

24 $${{{\mathcal{L}}}}_{{{\rm{affinity}}}}=\frac{1}{N}\mathop{\sum }\limits_{i=1}^{N}{{\rm{Huber}}}\left({\widehat{y}}_{i},{y}_{i}\right),$$

where ${\widehat{y}}_{i}$ and *y*_*i*_ are the predicted and true affinity values for valid samples *i*, and *N* is the number of valid samples.

To convert affinity values to experimental free energy Δ*G* values for comparison with physics-based models, we use the following formula:

25 $$\Delta G=-RT \ln 10\cdot pK,$$

where *R* = 1.987 × 10^−3^ kcal K^−1^ mol^−1^ is the universal gas constant and *T* = 298 K is the absolute temperature and pK being the respective affinity type.

#### Role of joint structure–affinity training

We note that the affinity head is architecturally lightweight, comprising four small MLPs that operate on pooled ligand, pocket, and interaction features, totaling ~743K parameters ( ~ 2.3% of the 33M total). The affinity Huber loss constitutes one of eight loss terms; the remaining seven—coordinate MSE, atom-type CE, bond-type CE, charge CE, hybridization CE, bond-length MSE, and bond-angle Huber—collectively dominate the gradient signal to the shared backbone by a substantial margin. While the affinity loss gradients do propagate through the shared representations during training, their relative contribution is minor, consistent with the well-established finding that multi-task learning from related auxiliary objectives provides implicit regularization rather than degradation of the primary task^68,69^. Multiple lines of empirical evidence underline this: (i) the comparison between FLOWR (no affinity head) and FLOWR.ROOT^base^ (with affinity head), trained from scratch on the same data and protocol, shows that all structural quality improvements (for example, PB-validity, strain energy) are attributable to explicit architectural enhancements (improved equivariant message-passing, residual coordinate updates, bond-length and bond-angle losses) rather than multi-task training; (ii) the unconditional model ${{{\rm{FLOWR.ROOT}}}}_{\,{{\rm{uncond.}}}}^{{{\rm{base}}}}$, which contains no pocket encoder or affinity head, independently achieves leading structural quality on GEOM-Drugs; and (iii) under affinity-guided importance sampling (see Methods), where the affinity head actively steers generation, structural quality metrics remain largely preserved (PB-validity: 0.93 → 0.91; strain energy: 48.46 → 54.42 kcal/mol), demonstrating robustness even under maximal affinity-head influence. The purpose of joint training is therefore not to improve structural generation, but to enable a unified workflow supporting in-distribution property-guided generation, joint structure–affinity domain adaptation via LoRA finetuning, multi-target selectivity campaigns, and affinity-annotated fragment elaboration—capabilities that would be lost entirely without the affinity head.

#### Confidence prediction: Predicted local distance difference test (pLDDT)

We also predict pLDDT confidence scores to assess the reliability of generated ligand structures. Given predicted ligand samples ${\widehat{l}}_{1}=(\widehat{X},\widehat{T},\widehat{Q},\widehat{H},\widehat{B})$ and ground-truth ligand-protein coordinates ${X}_{l}\in {{\mathbb{R}}}^{{N}_{l}\times 3}$ and ${X}_{p}\in {{\mathbb{R}}}^{{N}_{p}\times 3}$, we compute distance matrices $D,\widehat{D}\in {{\mathbb{R}}}_{+}^{{N}_{l}\times {N}_{p}}$ for true and predicted ligand-protein distances, respectively. We consider distances below 12.0 Å with ${b}_{i}=\,{{\rm{clamp}}}\,({\sum }_{j=1}^{{N}_{p}}({D}_{i,j} < 12.0),\min=1)$ neighbors for each ligand atom.

Using distance thresholds $\tau=\{\frac{1}{2},1,2,4\}$ and L1 distance errors $L=| D-\widehat{D}|$, the LDDT score for each ligand atom is obtained through

26 $${{{\rm{LDDT}}}}_{i}=\frac{1}{{b}_{i}}\mathop{\sum }\limits_{j=1}^{{N}_{p}}\left[\frac{1}{| \tau | }\mathop{\sum }\limits_{c\in \tau }({L}_{ij} < c)\right]\in (0,1).$$

Each atom’s LDDT score is binned into *k* = 50 categories for multi-class classification. The confidence head, *f*_*ϕ*_, shares the ligand decoder backbone with reduced depth (*l* = 8 layers), taking the generated structure ${\widehat{l}}_{1}$ as input and outputting invariant logits ${{{\rm{pLDDT}}}}_{i}\in {{\mathbb{R}}}^{50}$ for cross-entropy loss minimization.

#### Training and sampling overview

We summarize the training and sampling procedures in Algorithms 1 and 2. The complete ligand representation comprises coordinates **X**, atom types **A**, formal charges **Q**, hybridization states **H**, and bond types **B**. For brevity, Algorithms 1–2 detail only coordinates (continuous) and atom types (discrete, *F* categories); all other categorical features follow the identical discrete flow procedure.

##### Algorithm 1

**Training (one step)**

**Require**: Dataset ${{\mathcal{D}}}=\{({{{\mathcal{P}}}}_{i},{l}_{1,i})\}$, model *f*_*θ*_

1: Sample $({{\mathcal{P}}},{l}_{1})$ from ${{\mathcal{D}}}$ with *l*_1_ = (**X**_1_, **A**_1_, … )

2: Sample generation mode *m* (de novo, scaffold hopping, scaffold elaboration, linker inpainting, core/fragment growing, interaction-conditional)

3: Extract fragment mask **m** ∈ {0, 1}^*N*^ (*m*_*i*_ = 1: fixed; *m*_*i*_ = 0: variable; **m** = **0** for de novo)

4: Sample priors: ${{{\bf{X}}}}_{0} \sim {{\mathcal{N}}}({{\bf{0}}},{{\mathbf{\Sigma }}})$, **A**_0_ ~ Uniform({1, …, *F*})^*N*^ (one-hot)                 ⊳ **Σ**: anisotropic or **I**_3_

5: Overwrite fixed atoms: **X**_0,*i*_ ← **X**_1,*i*_,  **A**_0,*i*_ ← **A**_1,*i*_  ∀  *i* where *m*_*i*_ = 1

6: Optionally shift variable-atom prior to reference-ligand center of mass

7: Sample $t \sim {{\mathcal{U}}}(0,1)$; set *t*_*i*_ ← 1 for all fixed atoms (*m*_*i*_ = 1)

8: Interpolate: **X**_*t*_ = (1 − *t*)**X**_0_ + *t* **X**_1_ + *σ*(*t*) ***ϵ***, $${{\boldsymbol{\epsilon }}} \sim {{\mathcal{N}}}({{\bf{0}}},{{\bf{I}}})$$

9: Unmask atom types: *A*_*t*,*i*_ = *A*_1,*i*_ w.p. *t*, *A*_*t*,*i*_ = *A*_0,*i*_ w.p. 1 − *t* ⊳ Independently per atom

10: Forward: $({\widehat{{{\bf{X}}}}}_{1},{\widehat{{{\bf{A}}}}}_{1})={f}_{\theta }({l}_{t},t;\,{{\mathcal{P}}})$    ⊳ Model predicts target *l*_1_

11: $${{\mathcal{L}}}={\lambda }_{{{\rm{c}}}}\,{{\rm{MSE}}}({\widehat{{{\bf{X}}}}}_{1},{{{\bf{X}}}}_{1})+{\lambda }_{{{\rm{t}}}}\,{{\rm{CE}}}({\widehat{{{\bf{A}}}}}_{1},{{{\bf{A}}}}_{1})+{{{\mathcal{L}}}}_{{{\rm{aux}}}}$$    ⊳ See $${{{\mathcal{L}}}}_{{{\rm{total}}}}$$ above

12: Update $$\theta \leftarrow \theta -\eta \,{\nabla }_{\theta }{{\mathcal{L}}}$$

##### Algorithm 2

**Sampling / Inference**

**Require**: Pocket ${{\mathcal{P}}}$, optional fragment mask **m**, num. atoms *N*, steps *K*, Δ*t* = 1/*K*, model *f*_*θ*_, coord. noise *σ*_*c*_, inpainting frequency *N*_inp_≤*K* (default *K*)

1: Construct *l*^(0)^: ${{{\bf{X}}}}^{(0)} \sim {{\mathcal{N}}}({{\bf{0}}},{{\mathbf{\Sigma }}})$, **A**^(0)^ ~ Uniform({1,…,*F*})^*N*^ (one-hot); initialize *t* ← 0

2: If **m** ≠ **0**: fix coordinates/types for atoms with *m*_*i*_ = 1; set *t*_*i*_ = 1

3: **for***k* = 0,…,*K* − 1 **do**

4:  $$({\widehat{{{\bf{X}}}}}_{1},{\widehat{{{\bf{A}}}}}_{1})={f}_{\theta }({l}^{(k)},t;\,{{\mathcal{P}}})$$         ⊳ $${\widehat{{{\bf{A}}}}}_{1}={{\rm{softmax}}}(\cdot )$$

5:  ${v}_{t}^{\theta }=({\widehat{{{\bf{X}}}}}_{1}-{{{\bf{X}}}}^{(k)})\,/\,(1-t)$; *g*_*t*_ = (*t*+*ϵ*)^−1^ **1**[*t* < *t*_*c*_] ⊳ Velocity; inv. noise sched.

6:  ${{{\bf{X}}}}^{(k+1)}={{{\bf{X}}}}^{(k)}+\Delta t\cdot {v}_{t}^{\theta }$        ⊳ Euler step (ODE, default)

7:   or stochastic: ${s}_{t}=\frac{t\,{v}_{t}^{\theta }-{{{\bf{X}}}}^{(k)}}{1-t}$; ${{{\bf{X}}}}^{(k+1)}={{{\bf{X}}}}^{(k)}+\Delta t\,({v}_{t}^{\theta }+{g}_{t}\,{s}_{t})+\Delta t\sqrt{2{g}_{t}{\sigma }_{c}}\,{{\boldsymbol{\epsilon }}}$, ${{\boldsymbol{\epsilon }}} \sim {{\mathcal{N}}}({{\bf{0}}},{{\bf{I}}})$      ⊳ SDE

8:  $$P({{{\bf{A}}}}^{(k+1)}=s| {{{\bf{A}}}}^{(k)}=j)=\left\{\begin{array}{ll}\frac{\Delta t}{1-t}\,{\widehat{{{\bf{A}}}}}_{1,s} & s\ne j\\ 1-{\sum }_{s{\prime} \ne j}\frac{\Delta t}{1-t}\,{\widehat{{{\bf{A}}}}}_{1,s{\prime} } & s=j\end{array}\right.$$

9:  **A**^(*k*+1)^ ~ Cat(*P*)           ⊳ Discrete flow step

10:   If **m** ≠ **0and**$k\,{{\rm{mod}}}\,\,\lfloor K/{N}_{{{\rm{inp}}}}\rfloor=0$: restore fixed atoms from fragment               ⊳ Periodic inpainting

11:     *t* ← *t* + Δ*t*

12: **end for**

13: Final pass: $({\widehat{{{\bf{X}}}}}_{1},{\widehat{{{\bf{A}}}}}_{1})={f}_{\theta }({l}^{(K)},t\approx 1;\,{{\mathcal{P}}})$ ⊳ Corrector step

14: **return**$${\widehat{l}}_{1}=({\widehat{{{\bf{X}}}}}_{1},\,\arg \max {\widehat{{{\bf{A}}}}}_{1})$$

### Inference-time scaling

Inference-time scaling is a powerful approach that allocates additional computation during generation, rather than solely relying on larger architectures^70,71^. Originally developed for large language models, this approach has been extended to diffusion and flow matching models. Diffusion models leverage stochasticity through particle sampling^13,72^ and Feynman-Kac steering^73^, where multiple trajectories are generated and resampled based on reward functions. Flow matching models, despite being deterministic, achieve inference-time scaling through SDE-based generation, interpolant conversion, and Rollover Budget Forcing (RBF) for adaptive resource allocation^74^, improving upon diffusion-based advances^75^.

In our work, we steer generative trajectories to sample from the conditional (target) distribution

27 $${p}_{\phi }(l| y,P)\propto r(y| l,{{\mathcal{P}}}){p}_{\theta }(l| {{\mathcal{P}}}),$$

where *y* is a vector of properties such as binding affinity, and *r* represents a reward function that encodes user preferences.

#### Steering via Importance Sampling

The target distribution is usually intractable, especially when applying neural generative models that transport samples over time, such as flow or diffusion models. One way to sample from the target distribution is to generate *B* particles and resample them based on importance weights derived from scores $\exp (\lambda r(y,l,{{\mathcal{P}}}))$, following the principle of importance sampling.

The sequential Monte Carlo (SMC) method^76^ extends this concept to a time-sequential setting by maintaining *B* particles and updating their importance weights over time^72^. A straightforward weighting scheme involves evaluating the current batch of particles ${\{{l}_{i,\tau }\}}_{i=1}^{B}$ at time *τ*, computing their scores ${\{{\widehat{y}}_{i}=r({l}_{i,\tau },{{\mathcal{P}}})\}}_{i=1}^{B}$, and obtaining resampling weights through softmax normalization: ${w}_{i}=\frac{\exp ({\widehat{y}}_{i})}{{\sum }_{j}\exp ({\widehat{y}}_{j})}$. Particles are then resampled from the discrete distribution ${\{{l}_{i}\}}_{i=1}^{B} \sim \,{{\rm{Multinomial}}}\,(B,w=({w}_{1},{w}_{2},\ldots,{w}_{B}))$ with probability proportional to their weights, as done in PILOT^13^.

### Quantum mechanical calculations on protein-ligand complexes

To benchmark FLOWR.ROOT’s affinity prediction head, we perform quantum mechanical (QM) calculations on protein-ligand complexes and correlate the results with predicted affinities. Calculations were conducted using ULYSSES^77^, a general-purpose semi-empirical library offering multiple molecular Hamiltonians. We employ GFN2-xTB^78^ with ALPB aqueous solvation^79^. GFN2-xTB is a simplified density functional method that parametrizes interparticle interaction integrals against reference data, substantially accelerating calculations^78^-a critical advantage for large atomic systems such as protein-ligand complexes. Additionally, GFN2-xTB provides a balanced treatment of electrostatic interactions and incorporates state-of-the-art dispersion corrections, enabling accurate descriptions of non-bonded complexes. In this work, the QM validation of FLOWR.ROOT used full protein domains, containing over 5000 atoms. Such a system size, required for meaningful physics-based evaluations of the granular details distinguishing different ligands, necessitates using semi-empirical Hamiltonians. Further, we only consider non-covalent binders, and, for such cases, intermolecular interactions are treated at a level similar to full Density Functional Theory (DFT). Finally, we found in previous work that GFN2-xTB provides accurate evaluations of protein-ligand complexes, in agreement with experimental SAR data^62,80–82^.

Regarding the choice of GFN2-xTB for non-covalent protein-ligand binding energetics: (1) The SQM2.20 method demonstrated strong correlations between semi-empirical QM-derived binding energies and experimental data for multiple targets, including BACE1, which we also investigate—notably, such correlations were not observed with force-field approaches or contemporary ML scoring functions^83^. (2) GFN2-xTB was explicitly parameterized using non-covalent interaction datasets, employing the D4 dispersion correction and an updated electrostatics model^78^, making it particularly suited for non-bonded interactions that dominate protein-ligand binding. (3) Large QM regions are essential for reliably capturing binding energetics and selectivity effects; for example, DYRK1A and DYRK1B are highly homologous kinases (over 85% sequence identity), and treating only small binding-site regions would be insufficient to distinguish relative affinities. We emphasize that the QM calculations serve as an independent validation of predicted affinity trends rather than absolute binding energies.

Benchmark calculations on protein complexes extracted from the Schrödinger FEP dataset already contained protonated protein structures. For these cases, FLOWR.ROOT-generated ligands had to be protonated using open babel^84^. Calculations were run on the full complexes, water molecules were excluded, and solvation was treated only implicitly, except when otherwise stated. In the case of the kinase selectivity test, structures were extracted directly from the PDB and processed with Open Babel. This includes protonation of the protein pockets and of all ligands, along with their predicted total charges.

### Reporting summary

Further information on research design is available in the Nature Portfolio Reporting Summary linked to this article.

## Supplementary information

Supplementary Information

Transparent Peer Review file

Reporting Summary

## Source data

Source Data

## Data Availability

The following public datasets were used in this study: • Plinder [https://www.plinder.sh]^43^. • Binding MOAD. • SPINDR [10.5281/zenodo.15991056]^17^. • HiQBind [10.6084/m9.figshare.27430305]^42^. • SAIR^46^. • BindingNet [http://bindingnet.huanglab.org.cn]. • BindingNetv2 [http://bindingnetv2.huanglab.org.cn]^44^. • CrossDocked2020 [http://bits.csb.pitt.edu/files/crossdock2020/]^85^. • Kinodata-3D [10.5281/zenodo.10852507]^45^. • KIBA^86^. • Davis [https://tdcommons.ai/multi_pred_tasks/dti/#davis]^87,88^. • ZINC20^39^. • PubChem3D [https://ftp.ncbi.nlm.nih.gov/pubchem/Compound_3D/]^40^. • Enamine REAL. • OMol25 [https://huggingface.co/facebook/OMol25]^89^. • GEOM-Drugs [10.7910/DVN/JNGTDF]^90^. • OpenFE IndustryBenchmarks2024 [10.5281/zenodo.17245549]^51,52^. • PDE10A benchmark [10.1007/s10822-022-00478-x]^58^. The following co-crystal structures from the RCSB Protein Data Bank were used: 3PE1 [10.2210/pdb3PE1/pdb], 6KHF [10.2210/pdb6KHF/pdb], 4GIH [10.2210/pdb4GIH/pdb], 2Q70 [10.2210/pdb2Q70/pdb], 4ZSP [10.2210/pdb4ZSP/pdb], 3FT8 [10.2210/pdb3FT8/pdb], and 5SF4 [10.2210/pdb5SF4/pdb]. Curated training data, model checkpoints, and generated ligand sets are deposited on Zenodo [10.5281/zenodo.20069588] and on Google Drive [https://drive.google.com/drive/folders/1NWpzTY-BG_9C4zXZndWlKwdu7UJNCYj8?usp=sharing]. The four proprietary in-house structure–activity datasets used for the LoRA finetuning experiments in Fig. 5 are not publicly available, as they comprise commercially sensitive medicinal-chemistry data of Pfizer Inc. covering active drug-discovery projects. Additional data are provided in the Supplementary Information and the accompanying Source Data file. Source data are provided with this paper.

## Code availability

The source code for FLOWR.ROOT is publicly available on GitHub at https://github.com/jule-c/flowr_rootunder the MIT licence. The exact version of the code used to produce the results in this paper is archived on Zenodo at 10.5281/zenodo.20068203^91^. Pretrained model checkpoints are mirrored on Zenodo (see Data Availability).
