# Supplementary material for: FLOWR.ROOT – A flow matching-based foundation model for joint multi-purpose structure-aware 3D ligand generation and affinity prediction
Source: Nat Commun. 2026 Jul 6;17:5883. doi: 10.1038/s41467-026-74130-9 (PMC13338463; doi:10.1038/s41467-026-74130-9)
Supplement: Supplementary file 1 — Supplementary Information [file 41467_2026_74130_MOESM1_ESM.pdf]

FLOWR.ROOT – A flow matching based foundation model for joint multi-purpose structure-aware 3D ligand generation and affinity prediction: Supplementary Information

Julian Cremer[1,*], **Tuan Le**[1], **Mohammad M. Ghahremanpour**[2], **Emilia Sługocka**[3,4], **Filipe Menezes**[5,6,*], **Djork-Arné Clevert**[1]

[1]Machine Learning & Computational Sciences, Pfizer Worldwide R&D, Berlin, Germany

[2]Computational Chemistry, Medicine Design, Pfizer Worldwide R&D, Cambridge, USA

[3]Doctoral School of Medical and Health Sciences, Jagiellonian University Medical College, Cracow, Poland

[4]Department of Physicochemical Drug Analysis, Faculty of Pharmacy, Jagiellonian University Medical College, Cracow, Poland

[5]Institute of Structural Biology, Molecular Targets and Therapeutics Center, Helmholtz Munich, Neuherberg, Germany

[6]TUM School of Natural Sciences, Department of Bioscience, Bayerisches NMR Zentrum, Technical University of Munich, Garching, Germany

[*]Corresponding authors. Email: julian.cremer@pfizer.com, filipe.menezes@helmholtz-munich.de

May 13, 2026

Supplementary Information

Methods

FLOWR.UI: A graphical user interface for interactive ligand design

FLOWR.UI is a browser-based graphical user interface for interactive, structure-aware ligand design using the FLOWR.ROOT generative model (Supplementary Fig. 1). The application implements a two-tier client-server architecture: a CPU-bound FastAPI [117] frontend server handles molecular file management, input validation, property computation, interaction detection, and chemical space analysis, while a separate GPU-bound FastAPI worker service loads the FLOWR.ROOT model checkpoint and executes ligand generation. This separation enables deployment on HPC clusters where the frontend operates on login or CPU nodes and GPU resources are allocated on demand via SLURM, with automatic release after a configurable idle timeout.

FLOWR.UI supports both structure-based (SBDD, conditioned on a protein pocket) and ligand-based (LBDD, molecule-only) design workflows. Seven generation modes are available: (1) *de novo* generation, optionally conditioned on a reference ligand; (2) substructure inpainting, replacing user-selected atoms while retaining fixed substructures; (3) scaffold hopping, replacing the Murcko scaffold while preserving R-groups; (4) scaffold elaboration, decorating a retained ring scaffold with new functional groups; (5) linker inpainting, regenerating atoms connecting ring systems; (6) core growing, retaining a ring system core and regenerating the remainder; and (7) fragment growing, extending from a placed fragment with a configurable anisotropic Gaussian prior center. Users select atoms for retention or replacement directly in the 3D viewer; inpainting masks are computed in real time using pure RDKit-based [119] algorithms without GPU access.

The frontend renders protein structures as interactive 3D cartoon representations with optional molecular surfaces and binding-site residue highlighting (3Dmol.js [118]), 2D molecular depictions (RDKit.js [119]), and analytical plots (Plotly.js [120]). Gaussian prior clouds representing the spatial sampling distribution are visualized as 3D point clouds with interactive repositioning. Post-generation analysis includes chemical space projections via PCA, t-SNE [121], or UMAP [122] of Morgan fingerprints [123] (radius 2, 2048 bits), property distribution plots for 20 molecular descriptors (including molecular weight, LogP, TPSA, hydrogen bond donors and acceptors, rotatable bonds, ring counts, fraction of

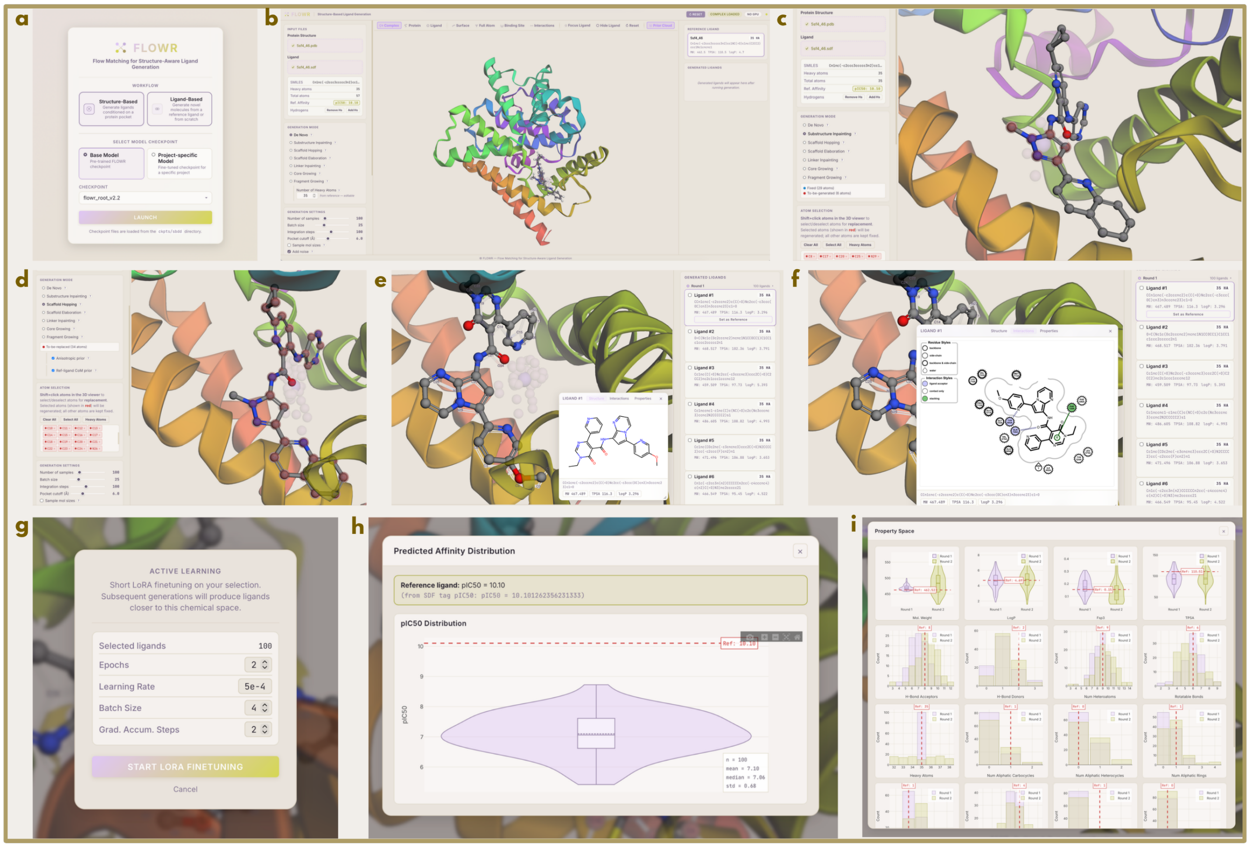

Supplementary Fig. 1. Overview of the FLOWR.UI graphical user interface for interactive ligand design. a Login and model selection page, where users choose between structure-based (SBDD) and ligand-based (LBDD) workflows and select a base model checkpoint or a project-specific finetuned model. **b** Main interface layout showing the interactive 3D protein-ligand viewer alongside generation mode selection, atom selection controls, and sampling parameter configuration. **c** Substructure inpainting example: user-selected atoms (highlighted) define the replacement region, with the corresponding anisotropic Gaussian prior cloud shifted to the selected substructure. **d** Scaffold hopping mode with optional user-guided atom selection for Murcko scaffold replacement while preserving R-groups. **e** Results panel displaying generated ligands with 2D structure depictions, interaction diagrams, and computed molecular properties (TPSA, LogP, molecular weight, among others). **f** Protein-ligand interaction diagram for a selected generated ligand, showing hydrogen bonds and salt bridges with distance annotations. **g** Active learning interface for LoRA finetuning of the loaded FLOWR.ROOT checkpoint, with configurable training parameters (number of epochs, batch size, learning rate). **h** Predicted potency distribution of generated ligands across generation rounds. **i** Molecular property distributions of generated ligands compared to the reference ligand.

sp^3 carbons, and structural alerts), affinity distribution tracking, and protein-ligand interaction visualization (hydrogen bonds and salt bridges) rendered as 3D distance-labeled overlays and 2D interaction diagrams.

Generated ligands undergo configurable post-processing: validity and uniqueness filtering, Tanimoto-based diversity filtering across generation rounds, RDKit [119] or GFN2-xTB [124] geometry optimization, property-based filtering, and optional ADMET model filtering. Results are accumulated over multiple rounds with cross-round diversity enforcement. Binding affinities (IC_{50}, K_i, K_d, EC_{50}) are automatically parsed from uploaded SDF property tags with unit detection and p-value conversion, enabling direct comparison between reference and generated compounds. FLOWR.UI further implements an active learning loop: users select promising ligands, trigger LoRA [125] finetuning of the loaded FLOWR.ROOT checkpoint on the GPU worker, and regenerate with the adapted model, enabling iterative structure-activity relationship refinement within a single session. All generated ligands can be ranked, selected, and exported as SDF files.

For HPC deployment, the server manages the full SLURM lifecycle—job submission with configurable resource

parameters, compute node discovery via `squeue`, worker health probing, and automatic GPU deallocation. Users access the interface via SSH port forwarding. File transfers between frontend and worker are authenticated using HMAC-based tokens, and the application enforces content security policies, rate limiting, and input sanitization.

Datasets

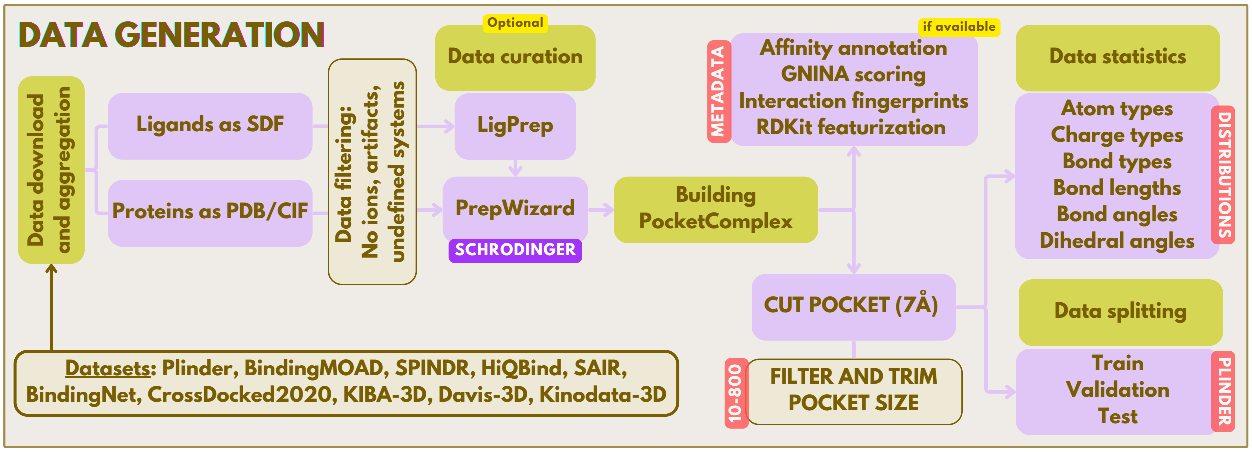

Supplementary Fig. 2. Overview of the dataset generation pipeline used in this work. Dataset generation workflow comprising data filtering, curation via Schrodinger's LigPrep and PrepWizard, building of metadata-annotated internal representation, and calculation of molecule statistics.

To comprehensively train and evaluate FLOWR.ROOT for structure-aware ligand design, we leverage a diverse collection of public datasets spanning both small molecules and biomolecular complexes. Our dataset selection encompasses three primary categories: (1) large-scale small molecule databases for training conformational and chemical diversity, (2) large-scale computationally generated datasets that bridge the gap between available experimental data and the scale and augmentation required for deep learning applications, and (3) small-scale experimental protein-ligand complex datasets for higher-fidelity structure-based modeling. This multi-faceted approach ensures robust model training across diverse chemical, conformational, and biological/bio-activity spaces while maintaining high structural quality standards.

For our small molecule database, we utilized Zinc3D, PubChem3D, Enamine REAL, and OMol25. For our protein-ligand database, we assembled a comprehensive collection of protein–ligand complex datasets by aggregating and standardizing data from multiple sources, including Plinder, BindingMOAD, SPINDR, HiQBind, SAIR, BindingNet, CrossDocked2020, KIBA-3D, Davis-3D, and Kinodata-3D. As visualized in Supplementary Fig. 2, after rigorous preprocessing, filtering, and preparation of both ligands and proteins, each complex was converted into a unified internal representation and annotated with extensive metadata, such as affinity values, if available, and molecular descriptors. This harmonized dataset enables systematic analysis of chemical composition, structural diversity, and affinity distributions across all included sources, providing a robust foundation for downstream modeling and benchmarking.

While SPINDR and HiQBind provide preprocessed and well-curated co-crystal data resources, we applied additional comprehensive curation to selected datasets, namely Plinder, BindingMoad, SAIR, KIBA-3D and Davis-3D using Schrödinger's LigPrep and PrepWizard tools. We used LigPrep to generate multiple protonated molecular conformations considering among other things different tautomeric states at physiological pH (7.4 $\pm$ 2.0), utilizing the OPLS4 force field and Epik for accurate pK_a prediction aligned with the reference ligand via the maximum common substructure (MCS). PrepWizard handled protein preparation through side chain completion, protonation state determination using Epik and PROPKA, termini capping, water molecule sampling within 10.0 Å, constrained hydrogen and overall restrained minimization (0.3 Å RMSD tolerance) using the S-OPLS force field. Unless otherwise stated, protein pockets were extracted using a 7Å cutoff radius around the respective reference ligands, with constraints of a minimum of 10 and maximum of 800 pocket atoms per complex to ensure computational tractability while preserving essential binding site information.

Importantly, throughout all protein-ligand datasets we kept a consistent dataset split following the provided

Plinder [1] train, validation and test set splits to avoid data leakage as best as possible enabling a stringent downstream evaluation. For more details about the datasets we refer to Supplementary Section , where we provide an overview of the aforementioned dataset generation, curation, and preprocessing pipeline, and a set of different dataset statistics.

Zinc3D Zinc3D [2] is a subset of the ZINC20 database containing pre-computed low-energy conformations for commercially available compounds. By providing ready-to-use 3D structures, ZINC3D eliminates the computational overhead of on-the-fly conformer generation, thereby accelerating virtual screening campaigns and structure-based drug discovery workflows. We utilized 646,663,126 molecules with their provided conformations.

PubChem3D PubChem3D [3] extends the widely-used PubChem database by providing up to 500 computed 3D conformations for millions of bioactive compounds. This resource offers extensive conformational diversity, crucial for training robust generative models. We employed 10 conformations per molecule from approximately 93 million compounds, resulting in 928,649,525 total conformations.

Enamine REAL The Enamine REAL database comprises commercially available, synthetically accessible compounds widely employed in virtual screening and drug design. Its well-curated chemical space supports generative design strategies focused on drug-likeness and synthetic feasibility. Following Cremer et al. [4], we used a diversity subset of the Enamine REAL database. We employed OpenEye's Omega software with default parameters to generate up to five conformers per molecule, yielding 111,389,149 conformations.

OMol25 OMol25 [5] is a comprehensive dataset containing over 100 million density functional theory (DFT) calculations at the ωB97M-V/def2-TZVPD level of theory. The dataset covers systems up to 350 atoms with exceptional chemical and structural diversity across 83 elements. We utilized two subsets: the small molecules collection (21,352,259 structures) comprising recomputed versions of widely-used datasets (ANI-2X, Orbnet Denali, SPICE2, Solvated Protein Fragments, and 30% of GEOM) upgraded to consistent high-level DFT theory, and the biomolecules subset (5,180,233 structures), encompassing protein-ligand interactions derived from BioLiP2 and additional complexes generated through docking drug-like molecules from GEOM, ChEMBL, and ZINC20. The biomolecule dataset features protein pocket environments processed through molecular dynamics simulation with appropriate capping and protonation state sampling.

KIBA-3D We introduce KIBA-3D, a kinase-focused dataset derived from the KIBA bioactivity dataset [6]. Using Schrödinger's Glide, we performed exhaustive cross-docking of the KIBA ligand space across 172 kinase targets, generating 333,670 protein–ligand complexes spanning 2,038 unique ligands. This dataset provides a dense bioactivity landscape ideally suited for affinity guided, pocket-conditioned generative modeling and kinase-specific benchmarking applications.

Davis-3D DAVIS-3D is a structurally augmented version of the original DAVIS kinase bioactivity dataset [7], created as part of the Folding-Docking-Affinity (FDA) framework [8]. The dataset was generated by computationally folding protein structures using ColabFold and determining protein-ligand binding conformations through DiffDock, a deep learning-based docking model. This process transforms the original sequence-based DAVIS dataset into a collection of three-dimensional protein-ligand binding structures, of which we utilized 12,982 complexes with binding affinities.

Kinodata-3D Kinodata-3D [9] is a curated collection of kinase complexes processed using cross-docking methodologies. We utilized 94,211 complexes from the combined mid- to higher-confidence subsets, which include binding affinity annotations and are preprocessed with a 5Å protein-ligand pocket cutoff.

BindingNet BindingNet [10, 11] comprises 689,796 modeled protein-ligand binding complexes across 1,794 protein targets. The dataset was constructed using an enhanced template-based modeling workflow that incorporates pharmacophore and molecular shape similarities alongside chemical similarity. Structures are categorized by template matching quality into high-confidence (232,030), moderate-confidence (164,912), and low-confidence (292,813) subsets, all of which we employed with their respective binding affinity annotations.

Plinder Plinder [1] represents the largest and most comprehensively annotated protein-ligand interaction (PLI) dataset, containing 449,383 PLI systems with over 500 annotations per complex. The dataset encompasses diverse interaction types including multi-ligand systems, oligonucleotides, peptides, and saccharides. Plinder introduced an approach for generating training and evaluation splits that minimizes task-specific leakage while maximizing test set quality. After removing complexes containing ligand artifacts, ions, or undefined ligands, and processing using Schrödinger's LigPrep and PrepWizard, we utilized 250,633 protein-ligand systems.

SAIR The Structurally Augmented IC50 Repository (SAIR) [12] is the largest publicly available dataset of protein-ligand 3D structures with binding affinity annotations, addressing the scarcity of high-quality experimental structures for deep learning applications. The original dataset contains 5,244,285 computationally generated structures across 1,048,857 unique protein-ligand systems from ChEMBL and BindingDB, with structures folded using the Boltz-1x model [13]. We applied stringent filtering criteria, retaining only PoseBusters-valid [14] complexes with negative AutoDock-Vina scores [15], confidence scores $\geq$0.8, interaction PTM $\geq$0.6, and IPTM $\geq$0.8, yielding 1,781,634 complexes. Further processing using Schrödinger's LigPrep and PrepWizard resulted in 1,564,677 curated complexes with IC50 annotations.

BindingMOAD BindingMOAD [16] is a comprehensive database developed over two decades (2001-2025), containing 41,409 protein-ligand complexes with affinity coverage for 15,223 complexes (37%) and 20,387 unique ligands. After preprocessing with Schrödinger's LigPrep and PrepWizard, we obtained 33,286 complexes with diverse binding affinity annotations.

SPINDR SPINDR [17] is a higher-fidelity dataset of protein–ligand complexes curated for interaction-aware modeling. The dataset emphasizes accurate binding site geometries and ligand poses, supporting physically consistent generative modeling. Complexes are thoroughly cleaned to focus on drug-like, non-covalent interactions using Schrödinger's PrepWizard, with protein-ligand interactions annotated using ProLIF [18]. We employed all 35,627 provided complexes with partial binding affinity annotations.

HiQBind HiQBind [19] is a curated dataset addressing structural artifacts in widely-used datasets, like PDBbind. Containing over 18,000 unique PDB entries and 30,000 protein-ligand complex structures, it matches binding free energies from BioLiP, Binding MOAD, and BindingDB with co-crystallized PDB complexes. The dataset employs strictly open-source curation tools with multiple quality control modules for steric clash detection, ligand structure fixing, protein completion, and hydrogen addition protocols. We utilized all 31,571 complexes with binding affinity annotations.

Schrodinger FEP+ dataset The Schrodinger FEP+ dataset [20] comprises a large and diverse collection of protein-ligand complexes, each featuring congeneric series of small molecules with experimentally measured binding affinities (K_d, K_i, or IC_{50}). Designed as a benchmark for assessing the accuracy and reproducibility of free energy perturbation (FEP) methods, the dataset emphasizes high-quality structural data, including X-ray structures and carefully curated protein and ligand preparations. It covers a broad chemical space, including challenging cases such as macrocycles, charge-changing transformations, and buried water displacement. This dataset is intended to support the development, validation, and comparison of computational methods for predicting relative binding affinities, providing a robust foundation for downstream applications in drug discovery and molecular design.

Data heterogeneity and quality control. The use of heterogeneous, multi-fidelity data—including computationally generated protein–ligand complexes—is a deliberate and principled design choice supported by both foundational machine learning theory and recent domain-specific evidence. Transfer learning research has consistently shown that pretraining on diverse, even noisy, data improves downstream task performance: Yosinski et al. [21] demonstrated that transferred features outperform random initialization even across distant tasks, while Mahajan et al. [22] showed that pretraining on 3.5 billion weakly labeled images transfers remarkably well to clean benchmarks. In physics, McCabe et al. [23] showed that a single model pretrained on heterogeneous physics simulations outperforms task-specific baselines when finetuned on unseen systems.

Within drug discovery, the value of computationally generated structural data has been independently validated. Valsson et al. [24] demonstrated that augmenting training data with BindingNet complexes improved FEP benchmark

performance (PCC $0.41 \rightarrow 0.59$, Kendall τ $0.26 \rightarrow 0.42$). Most directly relevant, Hsu et al. [25] systematically showed that augmentation benefits depend critically on structural quality: high-confidence BindingNet structures substantially improve binding affinity prediction ($\tau = 0.80$ correlation between PCC and training set size), while moderate- and low-confidence structures provide negligible or negative returns. Co-folded structures (Boltz-1x) were shown to substitute effectively for experimental structures when filtered by confidence.

Our pipeline implements quality control measures that align with these findings: (1) SAIR complexes are filtered from $\sim$ 5M to $\sim$ 1.5M using stringent multi-criteria thresholds (PoseBusters-validity, Vina scores, Boltz-1x confidence); (2) BindingNet complexes are stratified by confidence, with only the high-confidence subset treated as higher-fidelity data; and (3) the progressive training paradigm ensures that Stage 2 finetuning on curated experimental data (SPINDR, HiQBind) overrides residual noise from lower-fidelity Stage 1 sources.

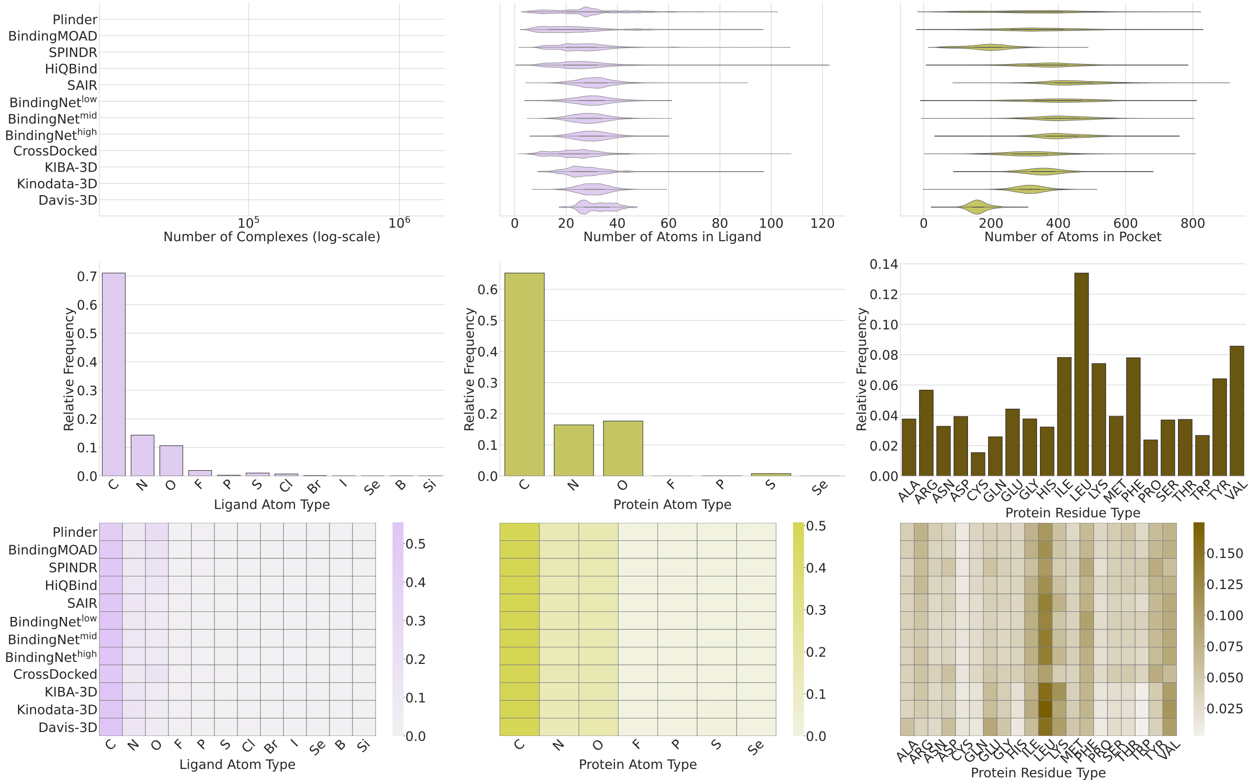

Supplementary Fig. 3. Dataset chemistry statistics. Distributions of ligand atom types, protein atom types, and protein residue types across multiple datasets. We show normalized frequencies of each type aggregated over all datasets, and per-dataset distributions visualized as heatmaps (logarithmic scale for atom types), highlighting differences in composition between datasets. Only the 20 standard amino acids are shown for residue types; hydrogens are excluded from atom type analyses. Per-dataset sample sizes (n, number of ligands or complexes) are listed in the corresponding paragraphs of Supplementary Section ; distributions are descriptive (no statistical test applied).

Dataset Statistics In Supplementary Section in Supplementary Fig. 2 we visualize the data generation framework that we established for this work. As outlined in Supplementary Section we used as protein-ligand complex data Plinder, BindingMOAD, SPINDR, HiQBind, SAIR, BindingNet, CrossDocked2020, KIBA-3D, Davis-3D, and Kinodata-3D. After downloading and aggregating the different data sources, we split proteins and ligands, if not already done, into separate files. We save ligands as SDF and proteins as PDB or CIF files. Afterwards, we filter the data and remove systems that classify the ligands either as ions, artifacts or not definable. Then, we use Schrodinger's LigPrep to prepare the ligands and Schrodinger's PrepWizard to prepare the protein-ligand complex (see Supplementary Section for more details). Successfully prepared systems are converted into our internal pocket-ligand representation encapsulated as PocketComplex instances for easy handling and data management. We retrieve pockets by cutting around the reference ligands with a cutoff of 7A and, if necessary, trim the pocket to the max size of 800 atoms. Every PocketComplex

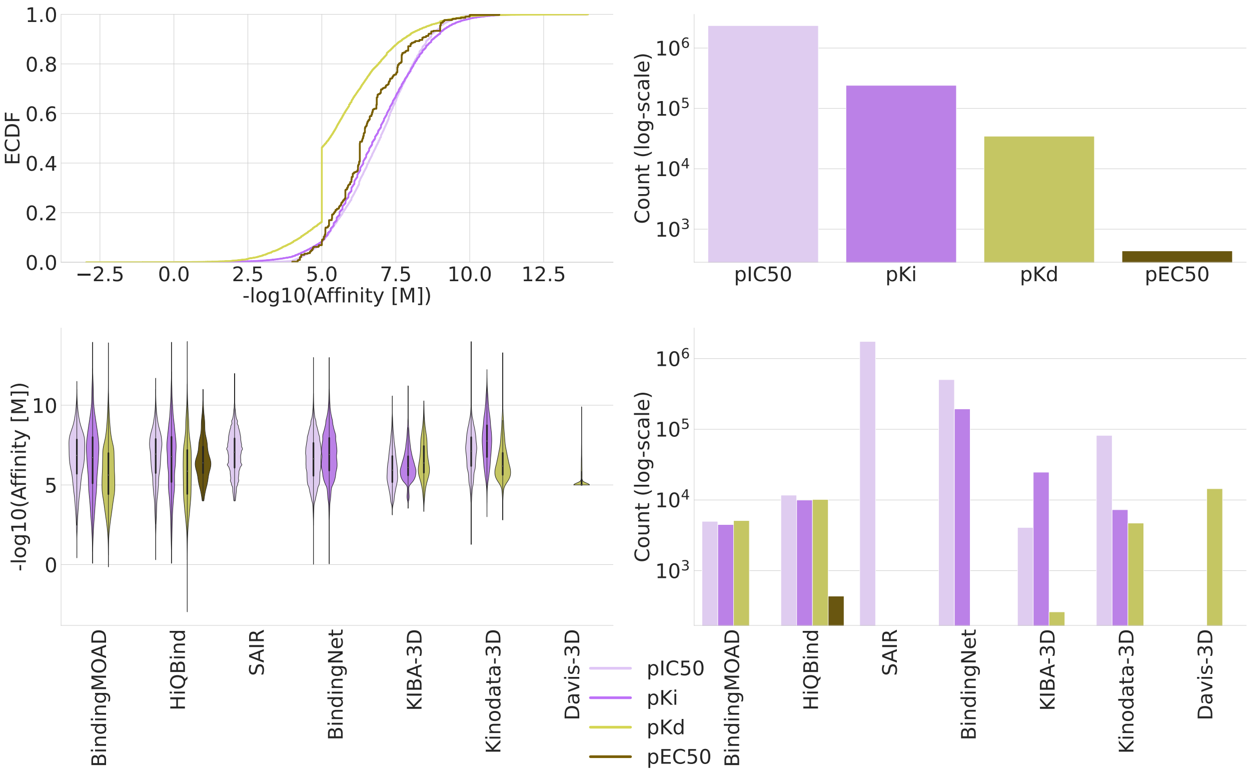

Supplementary Fig. 4. Dataset affinity statistics. Overview of affinity data distributions and dataset coverage. We show empirical cumulative distribution functions (ECDFs) of affinity values for each affinity type across all datasets; overall counts of each affinity type (log scale); and distribution of affinity values per dataset and affinity type (horizontal violin plots), as well as affinity type counts per dataset (horizontal bar plots, log scale). Legend indicates affinity type color coding used throughout all plots. Per-dataset sample sizes (n, number of affinity-annotated complexes) are listed in the corresponding paragraphs of Supplementary Section ; distributions are descriptive (no statistical test applied).

instance is annotated with comprehensive metadata spanning affinity values (if available), GNINA scores, interaction fingerprints, and selected RDKit features, like molecular weight, logP, TPSA, number of hydrogen acceptors and donors, number of rotatable bonds, number of aliphatic rings and many more. Finally, for all datasets we calculate distribution statistics comprising atom, charge, and bond type distributions as well as bond angles, bond lengths and dihedral angles distributions. Importantly, all datasets are splitted following the Plinder train, validation and test splits.

In Supplementary Fig. 3, we compared the chemical composition of all protein–ligand datasets used in this work by analyzing the distributions of ligand atom types, protein atom types, and protein residue types. The aggregated distributions (top row) reveal the overall prevalence of each type, while the per-dataset heatmaps (bottom row) illustrate how these distributions vary between datasets. For atom types, a logarithmic scale was used to emphasize less frequent elements. Only the 20 standard amino acids were considered for residue type comparisons, and hydrogens were excluded from atom type analyses to improve interpretability.

To provide a comprehensive overview of the affinity data, Supplementary Fig. 4 summarizes both the distribution and coverage of affinity types across all datasets. The ECDFs display the range and distribution of affinity values for each affinity type, highlighting differences in spread and modality. The overall affinity type counts, presented on a logarithmic scale, reveal a pronounced imbalance, with IC50 being the most abundant. Dataset-specific distributions are visualized using horizontal violin plots, while horizontal bar plots summarize the number of measurements for each affinity type within each dataset. The majority of affinity values originate from the SAIR and BindingNet datasets. Notably, BindingNet also provides Ki measurements, while HiQBind and BindingMOAD offer a balanced representation of IC50, Ki, and Kd values. HiQBind is the only dataset containing EC50 measurements. In contrast, Davis-3D is exclusively annotated with Kd values. This overview facilitates the identification of data biases and gaps that may influence downstream analyses.

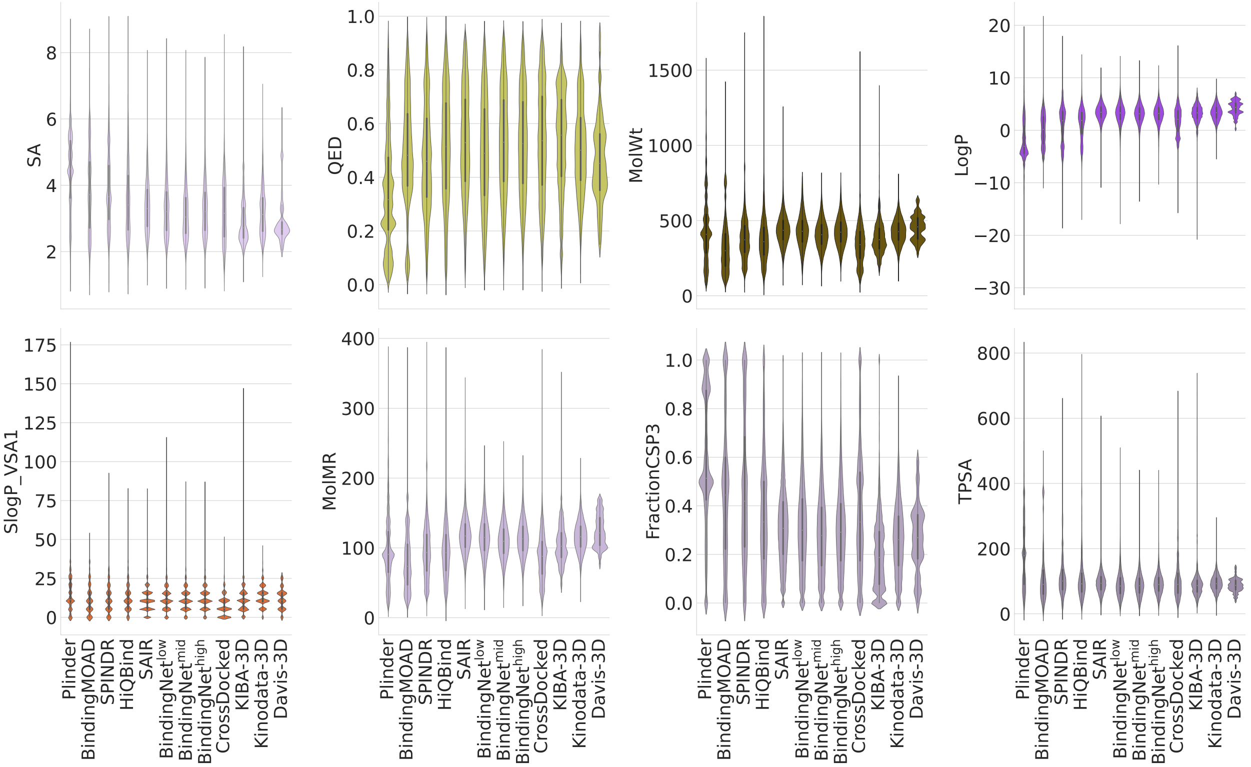

Supplementary Fig. 5. Dataset property statistics. Comparison of key continuous molecular properties of ligands—including molecular weight, logP, molar refractivity, and topological polar surface area—across all datasets. The distributions reveal substantial variability in ligand size, polarity, and hydrophobicity, reflecting the chemical diversity and distinct selection criteria of each dataset. Per-dataset sample sizes (n, number of ligands) are listed in the corresponding paragraphs of Supplementary Section ; distributions are descriptive (no statistical test applied).

To systematically assess the chemical diversity of ligands across datasets, we analyzed the distributions of several continuous molecular descriptors in Supplementary Fig. 5, including molecular weight, logP, molar refractivity, and topological polar surface area (TPSA). These features capture key aspects of ligand size, hydrophobicity, polarizability, and polarity, which are relevant for molecular recognition and drug-likeness. The results reveal pronounced differences between datasets: for example, BindingNet and SAIR contain ligands with a broader range of molecular weights and higher TPSA values, while datasets such as HiQBind and BindingMOAD exhibit more constrained distributions. These trends reflect the varying selection criteria and source domains of the datasets, and highlight the importance of considering chemical diversity in benchmarking and model development.

We further characterized the datasets by comparing the distributions of discrete ligand features in Supplementary Fig. 6, including the number of hydrogen bond acceptors and donors, rotatable bonds, ring systems, and chiral centers. These properties provide insight into molecular complexity, flexibility, and the presence of functional groups relevant for binding interactions. The analysis demonstrates that datasets such as BindingNet and SAIR encompass ligands with higher numbers of rotatable bonds and greater ring system diversity, whereas datasets like Davis-3D and KIBA-3D are more restricted in these aspects. Notably, the number of chiral centers and structural alerts also varies substantially, underscoring differences in stereochemical complexity and potential reactivity. Together, these comparisons elucidate the distinct chemical spaces sampled by each dataset and inform the interpretation of downstream modeling results.

KIBA-3D The KIBA dataset was originally introduced by Tang *et al.* [26] as a large-scale benchmark for kinase–ligand binding affinity prediction, integrating heterogeneous bioactivity readouts into a standardized KIBA score. The original dataset contained $52,498$ chemical compounds, 467 kinase targets and over $240,000$ interaction records. While comprehensive, the original KIBA dataset was highly scattered, due to an imbalance between the number of ligand-kinase pairs and associated measurements. To improve the density and ensure more reliable evaluation, subsequent

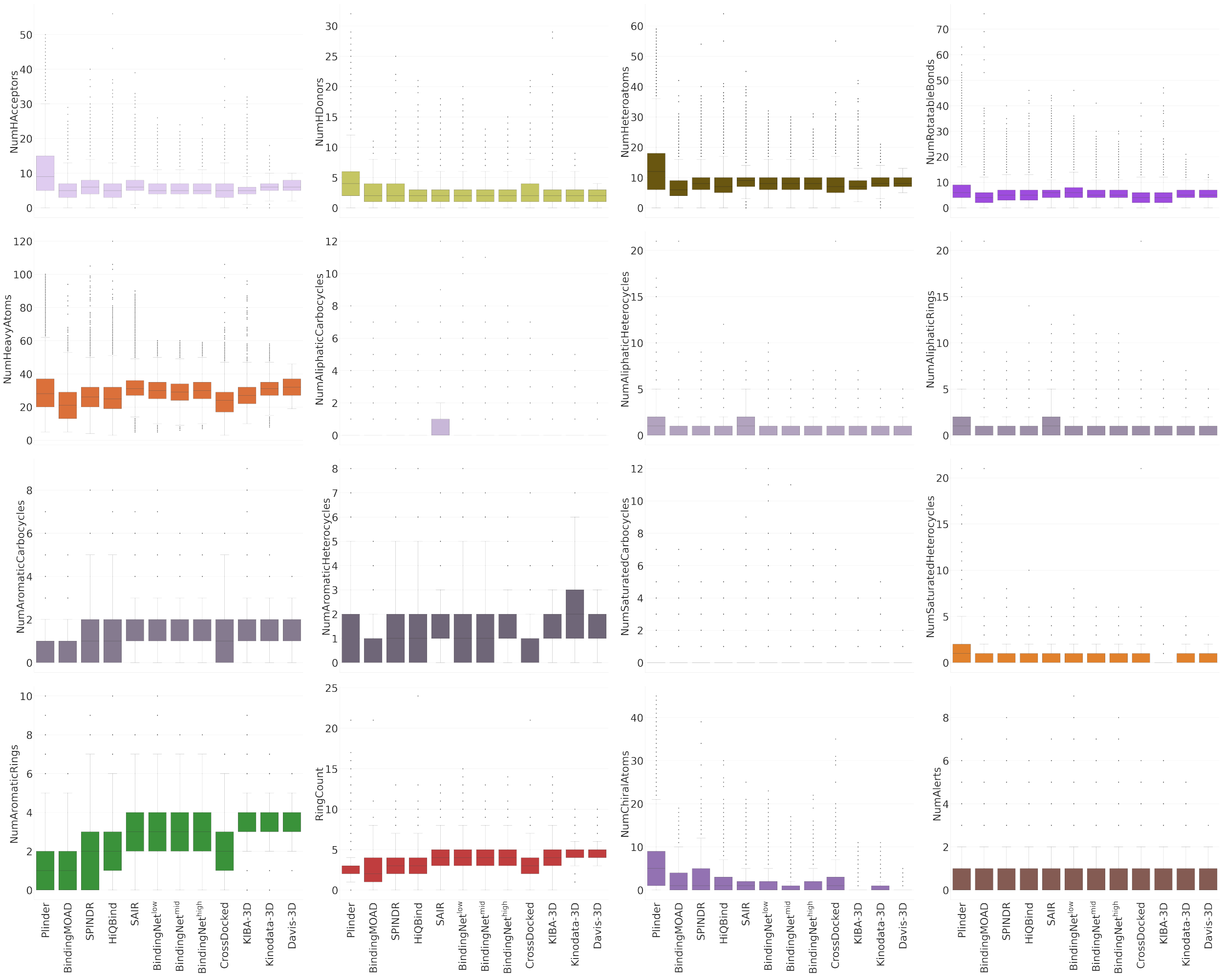

Supplementary Fig. 6. Dataset property statistics. Comparison of the distributions of discrete ligand features such as hydrogen bond acceptors and donors, rotatable bonds, ring systems, and chiral centers for each dataset. The observed differences highlight variations in molecular complexity, flexibility, and functional group composition among the datasets, providing insight into their respective chemical spaces. Per-dataset sample sizes (n, number of ligands) are listed in the corresponding paragraphs of Supplementary Section ; distributions are descriptive (no statistical test applied).

studies discarded the ligands and targets with less than 10 interaction records. This procedure reduced the dataset to $2,094$ ligands and 229 kinase targets. For this work, we prepared the 3D extension of KIBA (KIBA-3D) based on kinase targets with experimentally solved structures deposited in the PDB. Supplementary Fig. 7 shows examples of our docking results, illustrating that Glide could reproduce near-native binding poses for selected kinase inhibitors with conserved bidentate hydrogen bond pattern in the hinge region (for example, PDBID:2XB7-CHEMBL461876, glide gscore $-11,4$, PDBID:6LVM-CHEMBL1968590, glide gscore $-11,9$). The KIBA-3D dataset serves as a large-scale, structure-consistent benchmark for evaluating generative and predictive models.

Target protein structure selection for KIBA-3D Kinase targets from the KIBA dataset were mapped to their UniProt identifiers. For each UniProt accession, X-ray crystal structures were selected according to a set of heuristics to ensure biological relevance of the binding site. Only entries covering the kinase domain were considered. Among the available structures, the highest resolution one was chosen, with preference for holo over apo forms. Complexes with ATP, ADP, or staurosporine were deprioritized, as these ligands tend to stabilize non-representative conformations of the binding pocket; in particular, staurosporine, aside from being a highly promiscuous kinase inhibitor has a flat shape that inflate

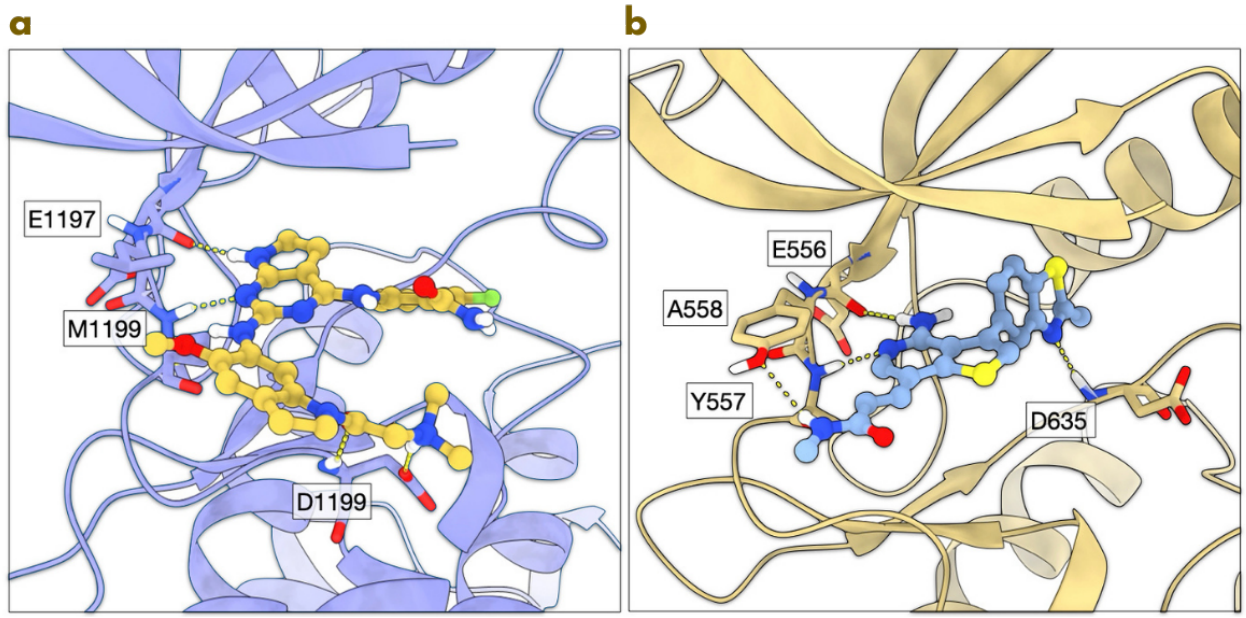

Supplementary Fig. 7. Example visualizations of protein-ligand complexes in KIBA-3D. a The structure of anaplastic lymphoma kinase (ALK, PDB ID: 2XB7, blue) with docked CHEMBL461876. The pyrrolopyrimidine moiety is positioned in the adenine region of the catalytic pocket, forming a bidentate hydrogen bond (yellow dashed lines) with the main chains of hinge residues Glu1197 and Met1199. **b** the structure of the tyrosine kinase fibroblast growth factor receptor 3 (FGFR3, PDB ID: 6LVM, yellow) accommodates an aminopyridine derivative (CHEMBL1968590) in a similar manner, with a conserved bidentate H-bond pattern in the hinge region (Glu556 and Ala558), complemented by an additional H-bond to the Tyr557 side chain. Furthermore, the nitrogen of the thiazole ring forms a hydrogen bond with Asp635 from the functional DFG motif. Both poses were favourable scored, with Glide docking gscore below -11 (-11.4 for the 2XB7–CHEMBL461876 complex and -11.9 for the 6LVM–CHEMBL1968590 complex). Representative panels: $n = 2$ docked complexes shown out of 333,670 Glide-docked KIBA-3D complexes spanning 2,094 unique ligands and 174 kinase targets.

the catalytic site and biases the pocket toward accommodating larger ligands. Structures with fewer missing residues were favored, and cases where the bound small molecule occupied an allosteric or noncanonical pocket were excluded. The resulting curated set provides 174 kinase structures suitable for docking of the KIBA ligand set.

Docking We performed docking with the Maestro suite (Schrödinger Release 2023-2: Maestro, Schrödinger, LLC, New York, NY, 2023) to generate consistent protein–ligand complexes across the 174 kinase targets. Small molecules were first optimized with LigPrep. All the 2094 ligands were processed. The dataset covers a broad chemical space, with molecular weights ranging from 180.15 to 1381.64 Da (median 376.49 Da). Protein structures were processed using the Protein Preparation Workflow, including protonation with Epik and propka [27] and restrained minimization with the OPLS4 force field [28]. Missing residues were built with Prime. Docking grids were centered on the kinase domain's hinge region, covering the canonical ATP-binding pocket used by first-type kinase inhibitors. Docking was performed with the Glide SP protocol [29], with no constraints applied, generating one pose per ligand.

Additional results

GEOM-Drugs and CrossDocked2020 Benchmarks Complete benchmark results on the unconditional GEOM-Drugs dataset and pocket-conditional CrossDocked2020 dataset are provided below, demonstrating that FLOWR.ROOT achieves leading performance across chemical validity, geometric accuracy, and energetic stability metrics.

On the GEOM-DRUGS dataset, FLOWR.ROOT achieves a PoseBusters-validity of 94.0%, surpassing all other models including FLOWMOL3 (91.9%). The model demonstrates exceptional geometric precision with a median relaxation RMSD of only 0.07 Å, substantially lower than all baselines. The relaxation energy of 3.65 kcal/mol indicates that

Supplementary Table 1. Evaluation and comparison of FLOWR.ROOT unconditional base model on GEOM-DRUGS. Benchmark comparison of the non-pretrained FLOWR.ROOT ligand-only base model against EQGAT-DIFF, ADIT, SEMLAFLOW, MEGALODON, FLOWMOL3 on the GEOM-DRUGS dataset. We follow the conventions in this field and sample 10,000 molecules with molecule sizes randomly sampled from the test set. We evaluate the performance of FLOWR.ROOT on RDKit- and PoseBusters-validity, the change in potential energy resulting from GFN2-xTB minimization ($\Delta E_{\mathrm{relax}}$) and the all-atom RMSD between the predicted and GFN2-xTB minimized conformations (Relax RMSD). For each model, $n = 10{,}000$ molecules were sampled per replicate; values are the sample mean with the 95% confidence interval over five replicate runs with different random seeds. For $\Delta E_{\mathrm{relax}}$ and Relax RMSD we report the median over the same $n = 10{,}000$ molecules per replicate.

MODEL	RDKIT-VALID(%)↑	PB-VALID(%)↑	$\Delta E_{\mathrm{relax}}$↓	RELAX RMSD↓	PARAMS (M)
EQGAT-DIFF	86.0 ± 0.9	77.6 ± 0.8	6.51 ± 0.16	0.60 ± 0.01	12
ADIT	99.9 ± 0.0	82.7 ± 0.8	79.32 ± 1.00	1.30 ± 0.02	150
SEMLAFLOW	95.5 ± 0.5	88.5 ± 1.3	31.9 ± 2.30	0.24 ± 0.03	40
MEGALODON	94.8 ± 0.3	86.6 ± 0.7	3.17 ± 0.11	0.41 ± 0.01	60
FLOWMOL3	99.9 ± 0.1	91.9 ± 0.7	3.83 ± 0.08	0.39 ± 0.01	6
FLOWR.ROOT$^{\mathrm{BASE}}_{\mathrm{UNCOND.}}$	98.5 ± 0.2	94.0 ± 0.2	3.65 ± 0.07	0.07 ± 0.02	34
TRAIN SET	100.0 ± 0.0	93.2 ± 0.1	-	-	

generated conformations are close to local energy minima.

Supplementary Table 2. Evaluation and comparison of FLOWR.ROOT base model on CROSSDOCKED2020. Benchmark comparison of the non-pretrained FLOWR.ROOT base model against POCKET2MOL, TARGETDIFF, DIFFSBDD, PILOT, DRUGFLOW and FLOWR on the CROSSDOCKED2020 test dataset. We follow the conventions in this field and sample 100 ligands per test target, of which there are 100. We evaluate the most expressive metrics, namely PoseBusters-validity, GenBench3D strain energy, AutoDock-Vina scores and the Wasserstein distance of the generated ligands' bond angles (BondA.W1) and bond lengths (BondL.W1) distributions relative to the test set. Sample sizes: $n = 100$ generated ligands per target across $n = 100$ CROSSDOCKED2020 test-set targets. Values are mean ± standard deviation;.

MODEL	PB-VALID↑	STRAIN↓	VINA SCORE↓	VINA SCORE^{MIN}↓	BONDA.W1↓	BONDL.W1 $[10^{-2}]$↓	SIZE	TIME (S)↓
POCKET2MOL	0.76 ± 0.39	147.22 ± 61.41	-4.72 ± 1.47	-5.80 ± 1.26	2.04	0.66	17.04 ± 4.11	2320 ± 45
DIFFSBDD	0.38 ± 0.46	519.03 ± 251.32	-2.97 ± 5.21	-4.71 ± 3.30	7.00	0.51	24.85 ± 8.94	160.31 ± 73.30
TARGETDIFF	0.57 ± 0.46	294.89 ± 136.32	-5.20 ± 1.79	-5.82 ± 1.60	7.76	0.42	22.79 ± 9.46	3228 ± 121
DRUGFLOW	0.75 ± 0.39	120.21 ± 73.28	-5.66 ± 1.78	-6.10 ± 1.62	2.11	0.38	21.14 ± 6.81	-
PILOT	0.83 ± 0.33	110.48 ± 87.47	-5.73 ± 1.72	-6.21 ± 1.65	1.75	0.33	22.58 ± 9.77	295.42 ± 117.35
FLOWR	0.92 ± 0.22	87.83 ± 74.30	-6.29 ± 1.56	-6.48 ± 1.45	0.96	0.27	22.28 ± 9.78	12.05 ± 8.01
FLOWR.ROOT^{BASE}	0.97 ± 0.22	67.13 ± 53.05	-7.76 ± 0.55	-7.93 ± 0.42	0.91	0.22	22.41 ± 8.95	15.43 ± 6.22
TEST SET	0.95 ± 0.21	75.62 ± 57.29	-6.44 ± 2.74	-6.46 ± 2.61	-	-	22.75 ± 9.90	-

On CROSSDOCKED2020, FLOWR.ROOT achieves a PoseBusters-validity of 0.97, surpassing all models including the test set reference (0.95). The strain energy of 67.13 kcal/mol is substantially lower than FLOWR (87.83) and PILOT (110.48), indicating energetically favorable binding poses. FLOWR.ROOT also achieves the best AutoDock-Vina score of -7.76 kcal/mol while maintaining efficient inference times (15.43s per ligand).

Pocket-conditional ligand generation: SPINDR We evaluate the non-pretrained FLOWR.ROOT^{base} model across three conditional generation modes and compare against the corresponding FLOWR.MULTI [17] baselines. For interaction-conditional generation, where the model is guided by predefined protein-ligand interactions, results are shown in Supplementary Table 4). We also assess scaffold inpainting, where functional groups are provided as context and the mod el generates the core scaffold (Supplementary Table 5). Conversely, for functional group inpainting, where the scaffold is given and the model decorates it with functional groups (Supplementary Table 6).

Supplementary Table 3. Benchmark of the (non-pretrained) FLOWR.ROOT^{base} model against FLOWR on the SPINDR test set. FLOWR.ROOT$^{base}_{large}$ denotes a base model with ∼1.5x the number of parameters to see the effect of model scaling on the SPINDR dataset. We report RDKit- and PoseBusters-validity of generated ligands, the GenBench3D strain energy and the AutoDock-Vina score. We also state the Wasserstein distance of generated ligands for the bond angles and bond lengths distribution to the SPINDR test set. Novelty, uniqueness and diversity measure the capability of the model to explore the chemical space both in 2D and 3D with the latter evaluating uniqueness and diversity of conformers of the same molecule (Note: if the list of generated ligands for a target contains duplicated molecules, otherwise they become zero). RDKit's QED evaluation, SAScore, the molecular weight as well as the logP values evaluate drug-likeness of generated ligands. All presented values are mean values taken for 100 sampled ligands per test set target. The test dataset comprises 225 test set targets. Note, both RDKit- and PoseBusters-validity are evaluated on the raw generated set of 100 ligands per target. All other metrics are calculated on the subset of RDKit-valid ligands. Values are mean ± standard deviation across n = 225 SPINDR test-set targets. Reported values are descriptive.

METRIC	TEST SET	FLOWR	FLOWR.ROOT^{BASE}	FLOWR.ROOT$^{BASE}_{LARGE}$
RDKIT-VALIDITY	1.00 ± 0.00	0.94 ± 0.24	0.98 ± 0.13	0.98 ± 0.14
PB-VALIDITY	0.99 ± 0.02	0.88 ± 0.21	0.97 ± 0.10	0.98 ± 0.09
STRAIN ENERGY	43.27 ± 41.85	90.05 ± 52.18	50.36 ± 34.59	47.67 ± 34.59
VINA SCORE	-7.69 ± 2.00	-6.93 ± 0.92	-7.52 ± 0.84	-7.56 ± 0.85
VINA SCORE (MINIMIZED)	-7.88 ± 2.00	-7.22 ± 0.92	-7.71 ± 0.85	-7.74 ± 0.85
BONDANGLESW1	-	1.08	0.60	0.68
BONDLENGTHSW1 [10^{-2}]	-	0.35	0.43	0.40
NOVELTY	1.00 ± 0.00	0.94 ± 0.23	1.00 ± 0.00	1.00 ± 0.00
UNIQUENESS2D	0.92 ± 0.10	0.94 ± 0.13	0.89 ± 0.18	0.89 ± 0.18
UNIQUENESS3D	-	0.50 ± 0.20	0.45 ± 0.22	0.43 ± 0.18
DIVERSITY2D	0.92 ± 0.04	0.86 ± 0.05	0.83 ± 0.10	0.82 ± 0.09
DIVERSITY3D	-	0.21 ± 0.12	0.13 ± 0.11	0.12 ± 0.11
SA	0.66 ± 0.12	0.67 ± 0.13	0.67 ± 0.14	0.66 ± 0.14
QED	0.49 ± 0.22	0.52 ± 0.21	0.49 ± 0.20	0.50 ± 0.20
RINGS	2.98 ± 1.42	2.68 ± 1.35	3.33 ± 1.52	3.41 ± 1.56
AROMATIC RINGS	1.84 ± 1.31	1.52 ± 1.16	2.07 ± 1.39	2.13 ± 1.35
HACCEPTORS	7.30 ± 4.49	6.67 ± 4.23	7.36 ± 4.78	7.39 ± 4.79
HDONORS	2.62 ± 1.68	2.52 ± 1.68	2.49 ± 1.67	2.50 ± 1.65
LOGP	0.29 ± 3.48	0.29 ± 3.31	0.59 ± 3.52	0.54 ± 3.45
MOLWT	390.43 ± 119.82	350.10 ± 114.00	384.26 ± 121.50	384.45 ± 121.68
LIPINSKI	4.00 ± 1.34	4.35 ± 1.05	4.25 ± 1.11	4.27 ± 1.10

Supplementary Table 4. Benchmark of the (non-pretrained) FLOWR.ROOT^{base} model against FLOWR.MULTI running interaction-/pharmacophore-conditional generation on the SPINDR test set. Given ligand atoms forming interactions with the protein pocket as context, the model generates the remaining structure. We report RDKit- and PoseBusters-validity of generated ligands, the GenBench3D strain energy and the AutoDock-Vina score. We also state the Wasserstein distance of generated ligands for the bond angles and bond lengths distribution to the SPINDR test set. Novelty, uniqueness and diversity measure the capability of the model to explore the chemical space both in 2D and 3D with the latter evaluating uniqueness and diversity of conformers of the same molecule (Note: if the list of generated ligands for a target contains duplicated molecules, otherwise they become zero). RDKit's QED evaluation, SAScore, the molecular weight as well as the logP values evaluate drug-likeness of generated ligands. All presented values are mean values taken for 100 sampled ligands per test set target. The test dataset comprises 225 complexes. Note, both RDKit- and PoseBusters-validity are evaluated on the raw generated set of 100 ligands per target. All other metrics are calculated on the subset of RDKit-valid ligands. Values are mean ± standard deviation across n = 225 SPINDR test-set targets. Reported values are descriptive.

METRIC	TEST SET	FLOWR.MULTI$^{interact.-cond.}$	FLOWR.ROOT$^{BASE}_{interact.-cond.}$
RDKIT-VALIDITY	1.00 ± 0.00	0.93 ± 0.25	0.98 ± 0.15
PB-VALIDITY	0.99 ± 0.02	0.86 ± 0.19	0.95 ± 0.11
STRAIN ENERGY	43.27 ± 41.85	107.60 ± 93.07	56.60 ± 43.19
VINA SCORE	-7.69 ± 2.00	-7.18 ± 0.83	-7.57 ± 0.67
VINA SCORE (MINIMIZED)	-7.88 ± 2.00	-7.48 ± 0.80	-7.82 ± 0.68
BONDANGLESW1	-	1.17	0.61
BONDLENGTHSW1 [10^{-2}]	-	0.43	0.37
NOVELTY	1.00 ± 0.00	0.93 ± 0.26	1.00 ± 0.00
UNIQUENESS2D	0.92 ± 0.10	0.83 ± 0.26	0.74 ± 0.32
UNIQUENESS3D	-	0.40 ± 0.21	0.36 ± 0.21
DIVERSITY2D	0.92 ± 0.04	0.82 ± 0.08	0.79 ± 0.10
DIVERSITY3D	-	0.06 ± 0.07	0.09 ± 0.08
SA	0.66 ± 0.12	0.67 ± 0.13	0.66 ± 0.13
QED	0.49 ± 0.22	0.50 ± 0.21	0.49 ± 0.20
RINGS	2.98 ± 1.42	2.98 ± 1.38	3.33 ± 1.52
AROMATIC RINGS	1.84 ± 1.31	1.79 ± 1.22	2.05 ± 1.34
HACCEPTORS	7.30 ± 4.49	7.23 ± 4.44	7.25 ± 4.60
HDONORS	2.62 ± 1.68	2.75 ± 1.58	2.72 ± 1.56
LOGP	0.29 ± 3.48	0.41 ± 3.43	0.35 ± 3.44
MOLWT	390.43 ± 119.82	379.85 ± 115.96	382.45 ± 117.86
LIPINSKI	4.00 ± 1.34	4.29 ± 1.11	4.31 ± 1.08

Supplementary Table 5. Benchmark of the (non-pretrained) FLOWR.ROOT^{base} model against FLOWR.MULTI on the SPINDR test set for scaffold hopping. Given functional groups as context, the model generates the remaining scaffold structure. We report RDKit- and PoseBusters-validity of generated ligands, the GenBench3D strain energy and the AutoDock-Vina score. We also state the Wasserstein distance of generated ligands for the bond angles and bond lengths distribution to the SPINDR test set. Novelty, uniqueness and diversity measure the capability of the model to explore the chemical space both in 2D and 3D with the latter evaluating uniqueness and diversity of conformers of the same molecule (Note: if the list of generated ligands for a target contains duplicated molecules, otherwise they become zero). RDKit's QED evaluation, SAScore, the molecular weight as well as the logP values evaluate drug-likeness of generated ligands. All presented values are mean values taken for 100 sampled ligands per test set target. The test dataset comprises 225 test set targets. Note, both RDKit- and PoseBusters-validity are evaluated on the raw generated set of 100 ligands per target. All other metrics are calculated on the subset of RDKit-valid ligands. Values are mean ± standard deviation across n = 225 SPINDR test-set targets. Reported values are descriptive.

METRIC	TEST SET	FLOWR.MULTI$^{\text{SCAFFOLD-INPAINT}}$	FLOWR.ROOT$^{\text{BASE}}_{\text{SCAFFOLD-INPAINT}}$
RDKIT-VALIDITY	1.00 $_{\pm 0.00}$	0.92 $_{\pm 0.26}$	0.98 $_{\pm 0.14}$
PB-VALIDITY	0.99 $_{\pm 0.02}$	0.86 $_{\pm 0.17}$	0.95 $_{\pm 0.12}$
STRAIN ENERGY	43.27 $_{\pm 41.85}$	105.32 $_{\pm 95.47}$	56.40 $_{\pm 44.10}$
VINA SCORE	-7.69 $_{\pm 2.00}$	-7.10 $_{\pm 0.71}$	-7.27 $_{\pm 0.63}$
VINA SCORE (MINIMIZED)	-7.88 $_{\pm 2.00}$	-7.34 $_{\pm 0.72}$	-7.51 $_{\pm 0.64}$
BONDANGLESW1	-	1.14	0.46
BONDLENGTHSW1 [10^{-2}]	-	0.58	0.33
NOVELTY	1.00 $_{\pm 0.00}$	0.87 $_{\pm 0.33}$	1.00 $_{\pm 0.00}$
UNIQUENESS2D	0.92 $_{\pm 0.10}$	0.70 $_{\pm 0.33}$	0.71 $_{\pm 0.29}$
UNIQUENESS3D	-	0.31 $_{\pm 0.20}$	0.35 $_{\pm 0.15}$
DIVERSITY2D	0.92 $_{\pm 0.04}$	0.78 $_{\pm 0.08}$	0.75 $_{\pm 0.10}$
DIVERSITY3D	-	0.07 $_{\pm 0.12}$	0.06 $_{\pm 0.06}$
SA	0.66 $_{\pm 0.12}$	0.65 $_{\pm 0.13}$	0.66 $_{\pm 0.13}$
QED	0.49 $_{\pm 0.22}$	0.49 $_{\pm 0.22}$	0.48 $_{\pm 0.21}$
RINGS	2.98 $_{\pm 1.42}$	2.93 $_{\pm 1.36}$	2.93 $_{\pm 1.37}$
AROMATIC RINGS	1.84 $_{\pm 1.31}$	1.60 $_{\pm 1.20}$	1.82 $_{\pm 1.30}$
HACCEPTORS	7.30 $_{\pm 4.49}$	7.54 $_{\pm 4.40}$	7.43 $_{\pm 4.49}$
HDONORS	2.62 $_{\pm 1.68}$	2.80 $_{\pm 1.67}$	2.69 $_{\pm 1.81}$
LOGP	0.29 $_{\pm 3.48}$	0.07 $_{\pm 3.46}$	0.30 $_{\pm 3.59}$
MOLWT	390.43 $_{\pm 119.82}$	382.87 $_{\pm 117.73}$	388.79 $_{\pm 120.44}$
LIPINSKI	4.00 $_{\pm 1.34}$	4.20 $_{\pm 1.18}$	4.20 $_{\pm 1.17}$

Supplementary Table 6. Benchmark of the (non-pretrained) FLOWR.ROOT^{base} model for functional group inpainting against FLOWR.MULTI on the SPINDR test set. Given a scaffold as context, the model generates the remaining functional groups. We report RDKit- and PoseBusters-validity of generated ligands, the GenBench3D strain energy and the AutoDock-Vina score. We also state the Wasserstein distance of generated ligands for the bond angles and bond lengths distribution to theSPINDR test set. Novelty, uniqueness and diversity measure the capability of the model to explore the chemical space both in 2D and 3D with the latter evaluating uniqueness and diversity of conformers of the same molecule (Note: if the list of generated ligands for a target contains duplicated molecules, otherwise they become zero). RDKit's QED evaluation, SAScore, the molecular weight as well as the logP values evaluate drug-likeness of generated ligands. All presented values are mean values taken for 100 sampled ligands per test set target. The test dataset comprises 225 test set targets. Note, both RDKit- and PoseBusters-validity are evaluated on the raw generated set of 100 ligands per target. All other metrics are calculated on the subset of RDKit-valid ligands. Values are mean ± standard deviation across n = 225 SPINDR test-set targets. Reported values are descriptive.

METRIC	TEST SET	FLOWR.MULTI$^{func.-group.-inpaint}$	FLOWR.ROOT$^{BASE}_{func.-group.-inpaint}$
RDKIT-VALIDITY	1.00 $_{\pm 0.00}$	0.93 $_{\pm 0.25}$	0.98 $_{\pm 0.15}$
PB-VALIDITY	0.99 $_{\pm 0.02}$	0.88 $_{\pm 0.13}$	0.98 $_{\pm 0.10}$
STRAIN ENERGY	43.27 $_{\pm 41.85}$	86.26 $_{\pm 78.31}$	52.52 $_{\pm 36.64}$
VINA SCORE	-7.69 $_{\pm 2.00}$	-7.41 $_{\pm 0.67}$	-7.35 $_{\pm 0.58}$
VINA SCORE (MINIMIZED)	-7.88 $_{\pm 2.00}$	-7.72 $_{\pm 0.59}$	-7.62 $_{\pm 0.59}$
BONDANGLESW1	-	0.84	0.58
BONDLENGTHSW1 [10^{-2}]	-	0.52	0.27
NOVELTY	1.00 $_{\pm 0.00}$	0.94 $_{\pm 0.23}$	1.00 $_{\pm 0.00}$
UNIQUENESS2D	0.92 $_{\pm 0.10}$	0.74 $_{\pm 0.28}$	0.75 $_{\pm 0.24}$
UNIQUENESS3D	-	0.35 $_{\pm 0.12}$	0.37 $_{\pm 0.15}$
DIVERSITY2D	0.92 $_{\pm 0.04}$	0.77 $_{\pm 0.07}$	0.75 $_{\pm 0.09}$
DIVERSITY3D	-	0.02 $_{\pm 0.01}$	0.06 $_{\pm 0.07}$
SA	0.66 $_{\pm 0.12}$	0.67 $_{\pm 0.13}$	0.66 $_{\pm 0.13}$
QED	0.49 $_{\pm 0.22}$	0.51 $_{\pm 0.21}$	0.51 $_{\pm 0.21}$
RINGS	2.98 $_{\pm 1.42}$	3.29 $_{\pm 1.46}$	3.27 $_{\pm 1.50}$
AROMATIC RINGS	1.84 $_{\pm 1.31}$	1.91 $_{\pm 1.33}$	1.84 $_{\pm 1.30}$
HACCEPTORS	7.30 $_{\pm 4.49}$	6.90 $_{\pm 4.21}$	7.00 $_{\pm 4.38}$
HDONORS	2.62 $_{\pm 1.68}$	2.61 $_{\pm 1.62}$	2.53 $_{\pm 1.73}$
LOGP	0.29 $_{\pm 3.48}$	0.83 $_{\pm 3.33}$	0.69 $_{\pm 3.29}$
MOLWT	390.43 $_{\pm 119.82}$	380.10 $_{\pm 115.69}$	381.96 $_{\pm 118.04}$
LIPINSKI	4.00 $_{\pm 1.34}$	4.40 $_{\pm 1.02}$	4.34 $_{\pm 1.07}$

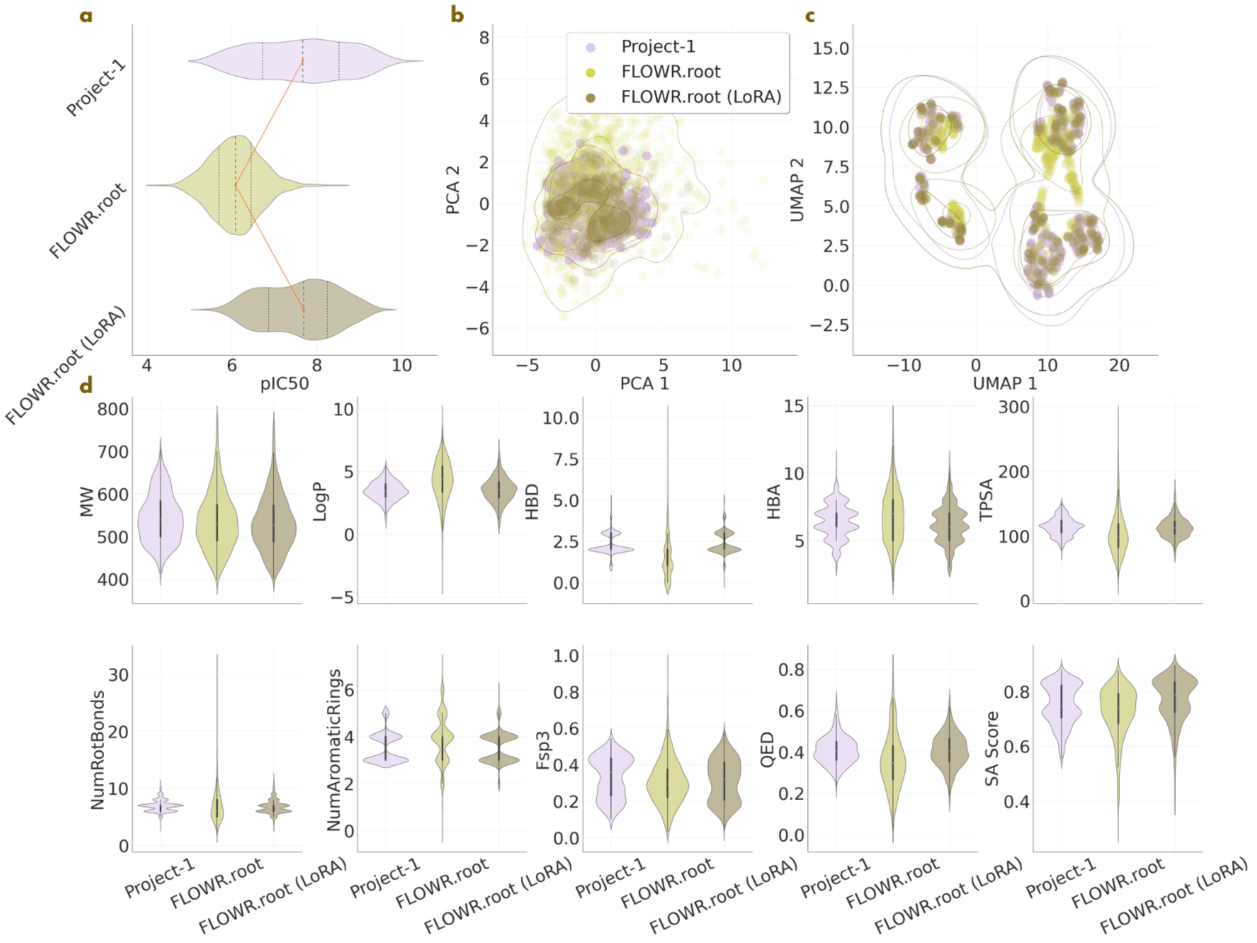

Supplementary Fig. 8. Evaluation of FLOWR.ROOT-generated samples on the in-house Project-1 test dataset. **a** Comparing the distribution of pIC_{50} values of generated ligands across test set complexes between FLOWR.ROOT and LoRA-finetuned FLOWR.ROOT. The pIC_{50} values for the test set are experimental, and otherwise predicted. **b** Depiction of chemical space comparison via PCA analysis showing the first two principal components. **c** Depiction of chemical space comparison via 2D UMAP analysis. **d** Distribution comparison regarding different chemical properties, namely molecular weight (MW), logP, number of hydrogen donors (HBD) and acceptors (HBA), topological surface area (TPSA), number of rotatable bonds (NumRotBonds) and aromatic rings (NumAromaticRings), fraction of sp^3 carbons (Fsp3), druglikeness (QED) and synthesizability (SA Score). Panels **a** and **d** show kernel density estimates over n =1000 generated ligands per test-set complex across n =1000 Project-1 test complexes;.

Domain Adaptation via Finetuning: Project-1 In Supplementary Fig. 8, we visualize the structure–activity landscape of the test data from one of our in-house project datasets, comparing it to the distribution of ligands generated by both FLOWR.ROOT and its LoRA-finetuned variant. Following finetuning, we observe substantial adaptation to the SAR characteristics of the in-house distribution across predicted potency as well as a broad range of chemical properties, including the number of hydrogen bond donors and acceptors, rotatable bonds, and TPSA. These results underscore the effectiveness of the proposed domain adaptation approach. As expected, FLOWR.ROOT performs substantially worse when comparing chemical feature distributions; however, structural fidelity remains high. The mean PoseBusters validity is 0.90±0.28, and the mean strain energy is 48.04±19.19 (compared to a mean reference strain energy 45.16±11.43). Thus, in contrast to the zero-shot affinity prediction capabilities of FLOWR.ROOT, zero-shot structure prediction yields physically realistic structures.

Domain Adaptation via Finetuning: PDE10A In Supplementary Fig. 9, we visualize FLOWR.ROOT-generated ligands using fragment replacement and growing, allowing the model to extend the quinoline ring of compound 5SF4_46 from the PDE10A dataset within the 5SF4 protein pocket. The generated ligands effectively explore hydrogen bonding

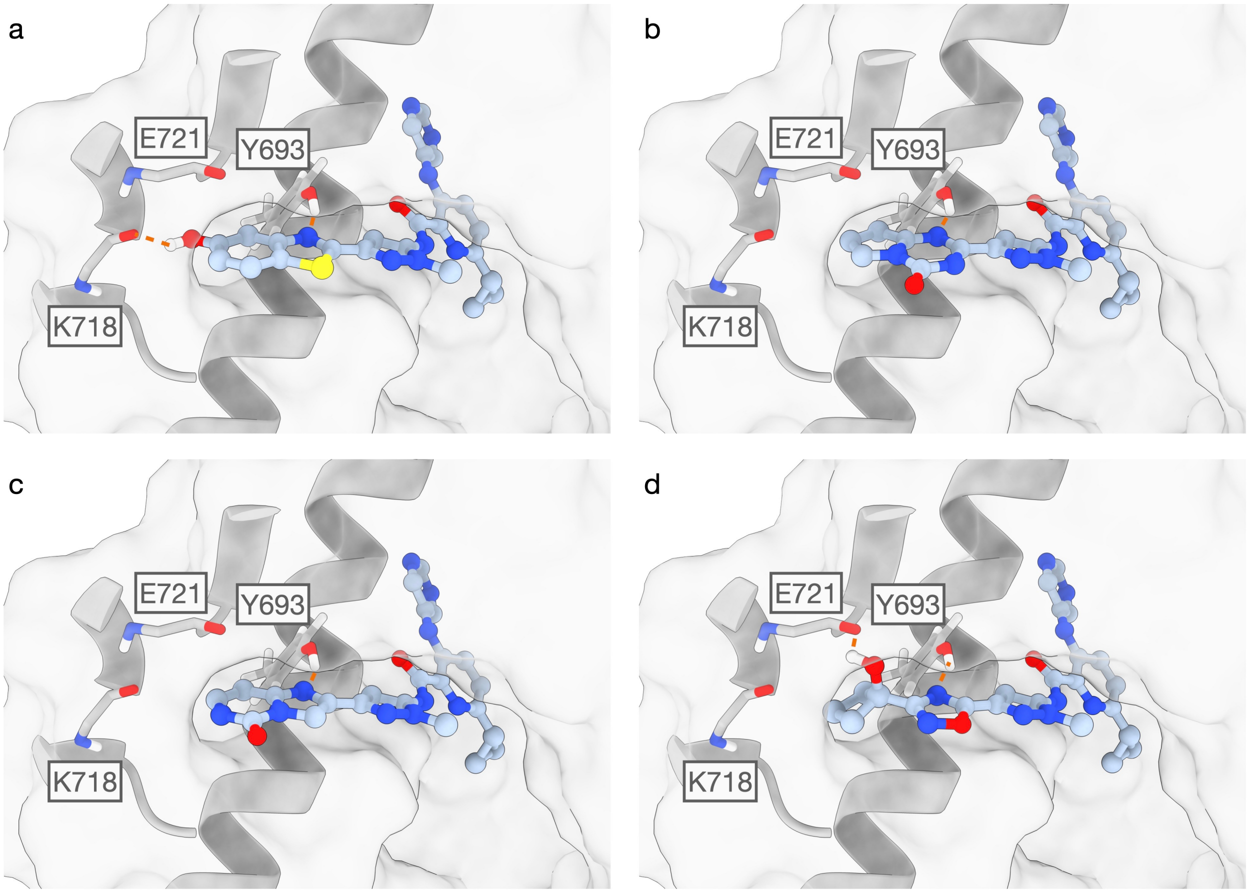

Supplementary Fig. 9. Visualization of FLOWR.ROOT-predicted ligands running fragment replacement on PDE10A (PDB ID: 5SF4). Several ligand chemotypes with different interaction patterns around the protein pocket. **a** capturing K718's main chain with a hydrogen bond. **b** and **c** similar chemotypes capable of avoiding interactions with the residues E721 and K718. **d** A ligand capable of capturing E721 with a hydrogen bond.

opportunities with the hydroxyl group of Y693 while extending in alternative directions, such as towards residues E721 and K718. In most cases, the generated ligands tend to avoid interactions with these residues. However, in two instances (Supplementary Fig. 9**a** and Supplementary Fig. 9**d**), the ligand developed functional groups capable of forming hydrogen bonds with the main chain atoms of E721 and K718. The generated structures maintain high physical plausibility, with a mean PoseBusters-validity of 0.89 ± 0.31 and a mean strain energy of 93.92 ± 15.48 kcal/mol (compared to a reference ligand strain energy of 83.50 kcal/mol).

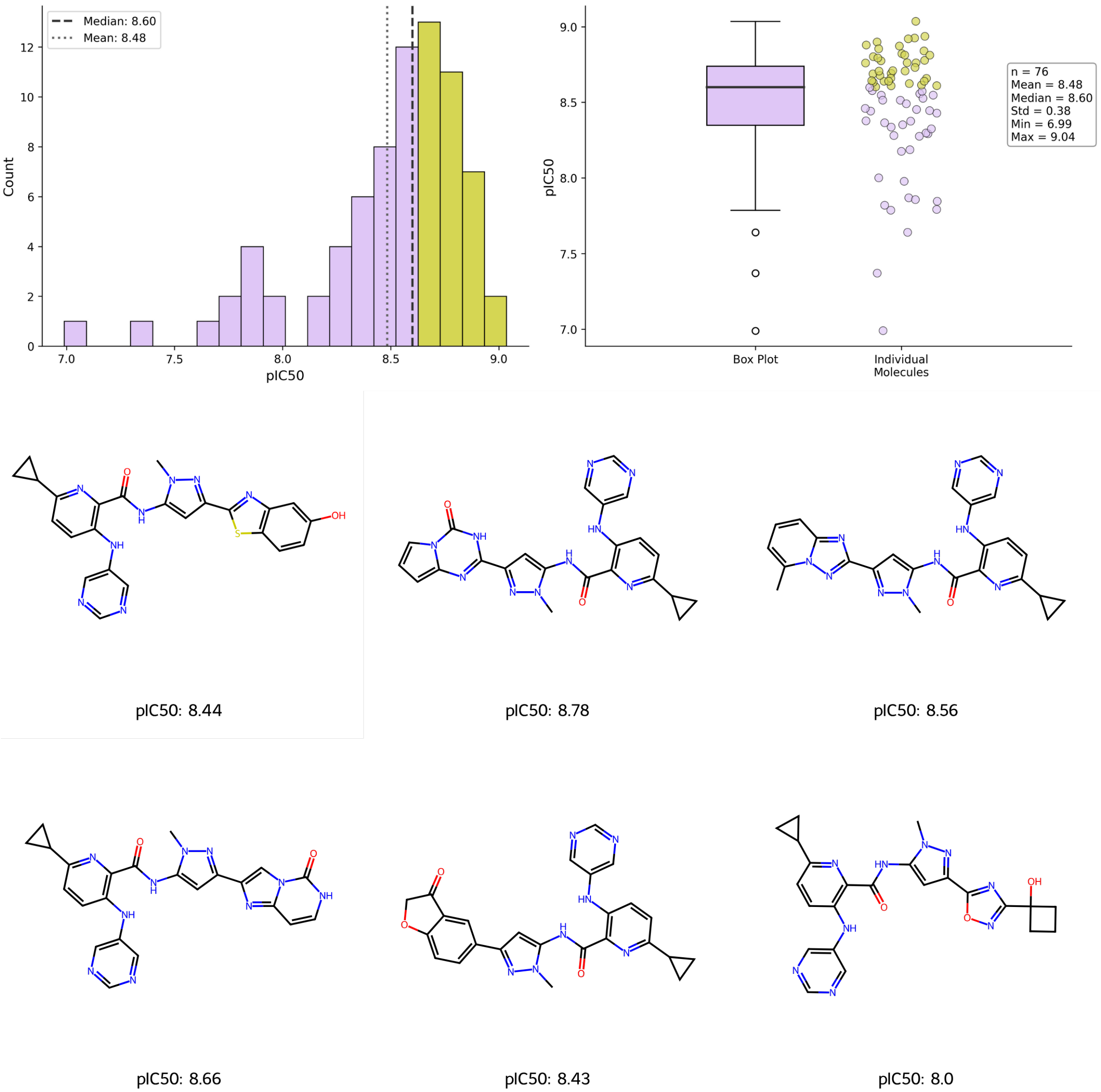

Supplementary Fig. 10. Analysis of FLOWR.ROOT-predicted ligands after running fragment replacement on PDE10A (PDB ID: 5SF4). Distribution over predicted pIC_{50} values of generated ligands within the 5SF4 protein pocket and a selection over promising candidates after interaction-analysis. Histogram over n =76 generated ligands.

Case studies

Hsp90: Heat shock protein 90 We also performed QM validation of FLOWR.ROOT's affinity head prediction on the heat shock protein 90 (Hsp90), Supplementary Fig. 11. Unlike the cases in the main text, the dynamic range of predicted affinities is substantially smaller, a consequence of the narrower pocket filled with water molecules. We performed two sets of benchmarks, on different sets: test1 includes more ligands with higher variation in functional groups (Supplementary Fig. 11**a-c**); test2 includes explicit water in the QM calculations (Supplementary Fig. 11**d-f**). In all cases, critical interactions, like the hydrogen bond to Asp93, are retained over the ligand space. In test1, FLOWR.ROOT tried to explore interactions in a lipophilic subpocket of Hsp90 by replacing a terminal phenyl ring with a pyridyl, to realize the latter is less likely to be stabilized in the lipophilic environment. The model also replaced the pyrimidine ring

with a quinazoline, in order to reduce the ligand's degrees of freedom, leading to less penalizing changes in entropy upon binding. This is seen when comparing the worst and best binders of the series. Finally, FLOWR.ROOT also tried to explore substitutions on the pyrimidine/quinazoline rings to further grow the ligand. As this is a solvent-exposed domain, NH_2 groups are favored and lead to better interaction patterns. In the case of test2, we explicitly included water molecules in the QM calculations, on top of implicit aqueous environment. This resulted in substantially better correlation between FLOWR.ROOT and the QM binding energies, although some of the poorer ligands had poorer QM scores due to soft clashes with the water molecules. This indicates that, although FLOWR.ROOT implicitly learns the composition and orientations of solvation layers, future work might involve giving the model the ability to capture these dynamically.

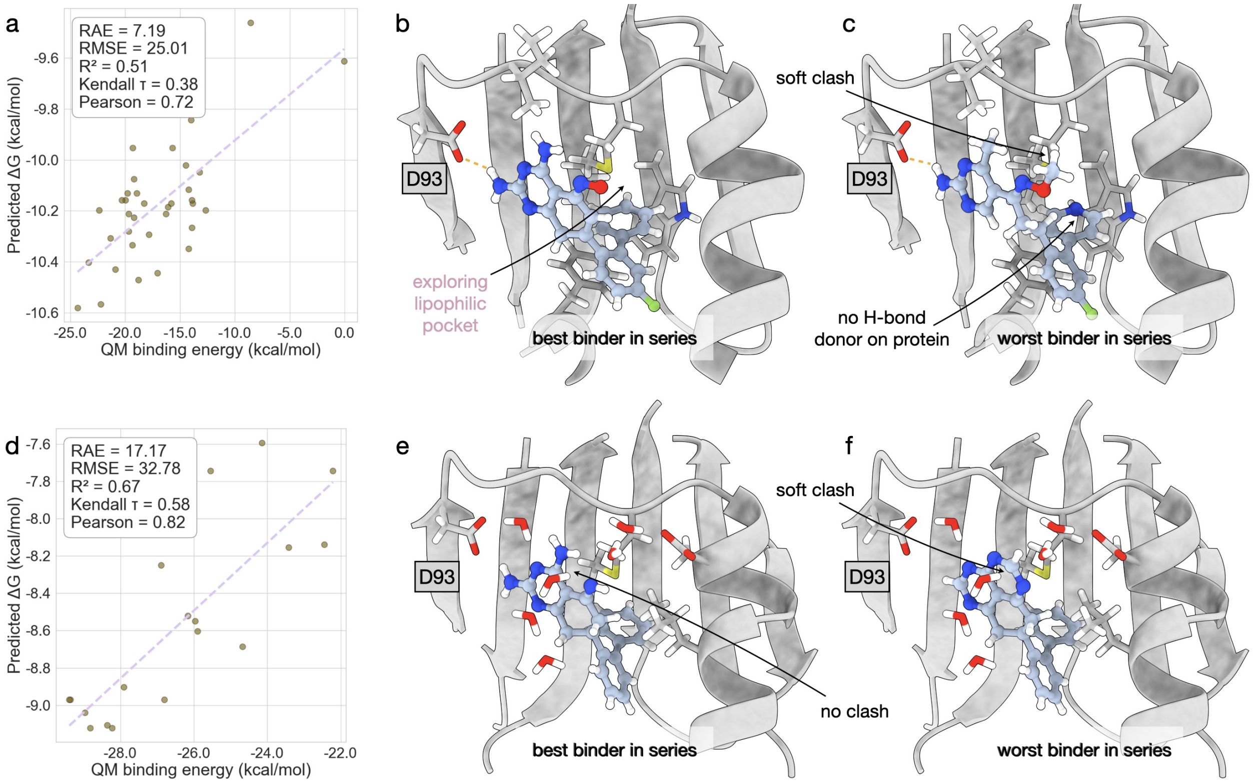

Supplementary Fig. 11. FLOWR.ROOT quantum mechanical validation on Hsp90. Test-1 validation, with more ligands but without explicit waters. **a** Correlation between FLOWR.ROOT affinity metrics and QM binding energies. **b** Schematic representation of the best binder in the series. **c** Schematic representation of the worst binder in the series. Test-2 validation, with explicit waters considered in the QM calculations. **d** Correlation between FLOWR.ROOT affinity metrics and QM binding energies. **e** Schematic representation of the best binder in the series. **f** Schematic representation of the worst binder in the series. In **a** and **d**, points are individual ligands (n =33 ligands for test-1; n =17 ligands for test-2). Reported correlation coefficients are Pearson's r from a two-sided test; exact p-values: **[p=??]**. HSP90 reference complex: PDB `3FT8`.

Related Works

Diffusion- and flow-based generative models have emerged as leading frameworks in machine learning [30, 31, 32]. Denoising Diffusion Probabilistic Models (DDPMs) learn a reverse process to transform noise into data samples [30], while score-based models formulate generation via reverse-time stochastic differential equations (SDEs) or their probability-flow ordinary differential equation (ODE) counterparts [31]. Flow matching, in turn, directly regresses the velocity field along a prescribed probability path to train continuous normalizing flows [33, 34], with stochastic-interpolant theory offering a unified perspective on diffusion and flow models [35, 36]. Beyond applications in natural language processing and computer vision, these models are increasingly applied to biology and chemistry, fueling

advances in generative chemistry for drug discovery [37, 38, 39, 40, 41, 4, 42, 43, 17]. In drug discovery, designing small molecules that selectively bind to specific protein targets remains a critical challenge. Diffusion and flow models have proven effective at capturing complex molecular and structural distributions, advancing methods from 3D molecular generation in structure-based drug design (SBDD) to protein–ligand (co-)folding [44, 45].

One important subfield of AI-driven SBDD is pocket-conditional, structure-aware ligand design. Models like DiffSBDD [40] and TargetDiff [41] have demonstrated that $SE(3)$-equivariant diffusion models can generate ligands directly within protein pockets. Follow-up studies have focused on improving chemical validity and pose accuracy [39, 49] while coupling pocket conditioning with large-scale pre-training and multi-objective importance sampling to guide generation toward potency and synthesizability [4]. Additionally, AI-driven models have increasingly integrated fragment priors into generative pipelines [50, 51, 52, 53, 54, 55]. In fragment-based drug discovery (FBDD), which focuses on hit-to-lead optimization, models typically specialize either in *de novo* design or fragment-based strategies. However, practical lead optimization requires flexible navigation between these regimes, such as fragment growing or scaffold hopping under interaction constraints, which most current frameworks do not adequately address. Furthermore, many models do not incorporate the geometric constraints imposed by protein pockets. Recent work proposes a unified flow matching approach that integrates *de novo*, interaction-constrained, and fragment-based generation under pocket conditioning with efficient ODE sampling [17].

However, the effectiveness of generated ligands ultimately depends on their potency and binding affinity, which requires reliable affinity prediction. While experimental validation remains indispensable, computational prioritization during the design phase relies on accurate affinity predictions to effectively navigate target-relevant regions of chemical space. Classical scoring functions, such as AutoDock Vina [56], Glide [57], and GOLD [58], offer computational efficiency but often lack the necessary accuracy for reliable prioritization. Physics-based methods like free energy perturbation (FEP) and absolute binding free energy (ABFE) calculations provide higher precision [59, 60, 61, 62, 63, 64], yet their computational cost prohibits application to large-scale generative campaigns, restricting their use to small subsets of candidates where absolute affinity data is critical. Machine learning–based scoring functions improve throughput but suffer from dataset bias and limited generalization [65]. Recent approaches using data augmentation have shown modest improvements [24], though they lack explicit structural information and remain insufficient for robust affinity prediction. The Boltz-2 ligand–protein co-folding model achieves near-FEP accuracy on selected targets with substantial speed advantages [45]. However, its decoupled architecture—where the affinity head is trained independently from the structural module—creates dependence on structure prediction quality, requires co-folding prior to each affinity prediction, and may limit co-adaptation between protein pocket and ligand geometry, particularly during project-specific finetuning.

These limitations motivate a framework that jointly learns structure and affinity while supporting fast, controllable, structure-aware generation. A model that learns the joint probability distribution over ligand geometry, binding pose, and affinity within the protein pocket context would enable efficient, potency-guided ligand generation with on-the-fly ranking capabilities. Crucially, joint training facilitates simple project-specific adaptation through finetuning—an essential capability given the distinct constraints of drug discovery campaigns, ranging from ADME/T requirements to R-group and scaffold novelty constraints, diverse assay readouts, and medicinal chemistry heuristics. A fundamental challenge in structure-based generative modeling lies in the inherent disconnect between public-domain training data and project-specific structure-activity relationships (SARs). While models can achieve broad coverage of chemical space through ligand generation, generalizing across unseen bioactivity landscapes represents a fundamentally more complex problem. We posit that expecting universal generalization without adaptation is unrealistic; instead, models should function as dynamic companions that continuously refine their understanding of project-specific SARs through sustained interaction with incoming data. This paradigm shift necessitates moving from static models to efficient iterative refinement processes where model utility grows through continuous adaptation. Additionally, techniques such as multi-objective guidance via inference-time importance sampling [4] can help steering generation toward pre-specified, desired properties, while deeper distribution mismatches or activity cliffs can be addressed through direct preference alignment [66] when suitable data is available.

While large ligand-only resources such as ZINC and PubChem offer abundant chemical diversity [2, 3], high-quality protein–ligand complexes with reliable affinity annotations remain scarce and noisy. Databases like PDBbind provide valuable training data [67], yet suffer from quality and coverage limitations; curated subsets such as HiQBind address these issues and even increase sample sizes [19], yet remain limited in scale. The Plinder dataset [1] expands scale by broadly curating the PDB, but most entries lack affinity annotations. More recent resources—BindingNet [11], Kinodata-3D [9], and especially SAIR [12]—improve chemical space coverage and include affinity data, though they do so with reduced accuracy. This heterogeneous data landscape, however, motivates a multi-stage training paradigm:

by systematically combining available resources ranked by fidelity, we can exploit their complementary strengths. Large-scale, lower-fidelity data establishes broad chemical space coverage and foundational structural understanding, while subsequent refinement on higher-quality, curated datasets sharpens affinity prediction and structural accuracy. Critically, this approach yields foundation models capable of efficient adaptation to project-specific objectives.

In the following, we discuss related works in more details for 3D molecular generation, pocket-aware ligand design, fragment-based drug discovery, and binding affinity prediction methods.

Molecule generation Early neural approaches to 3D molecular generation explored autoregressive models that sequentially add coordinates and atom types while maintaining geometric consistency. Symmetry-aware models such as G-SchNet [68] and its conditional inverse-design follow-up [69] demonstrated that enforcing $E(3)$ symmetries and conditioning signals can substantially improve the validity and controllability of generated 3D structures. Subsequent work proposed explicit autoregressive flows [70]. In parallel, the diffusion modeling paradigm matured from its non-equilibrium thermodynamics roots [71] through score-based [31] and variational formulations [72, 73]. These ideas rapidly translated to molecular geometry: Xu et al. [47] and Jing et al. [74] showed that $E(3)$-equivariant denoisers enable high-quality conformation generation by diffusing in Cartesian and torsional spaces, respectively.

The first $E(3)$-equivariant diffusion model for joint continuous coordinate and atom type generation was EDM [37]. Follow-ons pushed the design space along several axes: discrete and continuous combination with explicit bond order learning [38], and enhanced denoising learning objectives [39], establishing strong baselines.

Thereafter, diffusion-based methods pioneered pocket-aware *de novo* design, also utilizing $E(3)$-equivariant networks. Notably, DiffSBDD [40] and TargetDiff [41] demonstrated that conditional diffusion models can generate diverse, target-specific ligands within the protein pocket, adhering to symmetry constraints and enabling task versatility through sampling controls. More recently, PILOT [4] combined large-scale pre-training, pocket conditioning, and property guidance, highlighting the importance of multi-objective steering (for example, drug-likeness, synthesizability) under structure constraints. Together, these works established that pocket-aware 3D diffusion can simultaneously respect symmetry, improve pose realism, and support versatile constraints via conditioning and guided sampling.

Meanwhile, flow matching [33] (FM) was proposed refining continuous normalizing flows by directly regressing a time-dependent velocity field that pushes a simple prior to the data distribution, offering faster sampling and flexible priors. SEMLAFLOW [43] introduced a scalable $SE(3)$-equivariant architecture (Semla) trained via flow matching, achieving state-of-the-art unconditional 3D molecule generation with substantial speed-ups. Pushing FM into SBDD, FLOWR [17] extended the Semla-style backbones with a dedicated pocket encoder and mixed continuous/categorical FM, supporting multi-mode *de novo*, interaction-guided, and fragment-based generation in a single model. FLOWR reports large speedups over pocket-diffusion baselines.

Fragment-based drug discovery Fragment-based drug discovery (FBDD) motivates models that initiate from fragments and perform growth, linking, or merging under pocket constraints. Early deep generative linker design incorporated 3D information into graph models (DeLinker) [50], while SyntaLinker and AutoLinker utilized conditional transformers to synthesize linkers directly in the SMILES space, given fragment pairs and constraints [75, 76]. Recent advances have introduced $E(3)$-equivariant models: DiffLinker formulates linker generation as an $E(3)$-equivariant conditional diffusion, explicitly learning 3D geometry between fragment anchors [55]. For broader medicinal-chemistry workflows, Link-INVENT extends REINVENT with reinforcement learning to optimize linkers for multiple objectives, demonstrated on fragment linking, scaffold hopping, and PROTAC design [54]. STRIFE extracts target-specific pharmacophoric features to steer elaboration in 3D [77], while AutoFragDiff integrates fragment-wise, autoregressive diffusion with pocket conditioning to improve local 3D geometry during growth [78]. For scaffold hopping, DiffHopp employs an $E(3)$-equivariant graph diffusion model tailored for scaffold replacement conditioned on a protein–ligand complex [79], and TurboHopp accelerates pocket-conditioned 3D scaffold hopping with consistency models and reinforcement learning-based preference optimization [80].

Binding Affinity Prediction Estimating the change in free energy upon binding (ΔG_{bind}, or affinity) accurately remains a cornerstone of structure-enabled small-molecule discovery. Binding affinity is relevant for all early stages of drug discovery, starting from hit identification, where the goal is to find tight and selective binders, through hit-to-lead and lead optimization, where potency must be balanced with absorption, distribution, metabolism, excretion, safety, toxicity, and efficacy considerations. Given the astronomical size of chemical space, computer-aided drug

design (CADD) is indispensable to select and prioritize candidates *in silico* before spending scarce experimental resources [81, 82].

Classical structure-based approaches to affinity prediction span knowledge-based scoring and physics-based models grounded in molecular mechanics [83, 84]. Heuristic docking scores offer speed at the expense of physical rigor. Empirical scoring functions such as AutoDock Vina, Glide, or GOLD remain widely used due to speed, but show inconsistent results between targets [56, 57, 58]. Semi-empirical and QM / MM scoring have closed part of the gap at an intermediate cost, for example, SQM2.20 achieves DFT-quality affinity estimates in minutes, but only on selected targets [85, 86].

End-point methods such as MM-PBSA and MM-GBSA combine molecular mechanics with continuum solvation to approximate the ΔG_{bind} from MD snapshots at a relatively low cost and remain widely used when throughput is critical [87, 88, 89, 90]. Alchemical binding free energy methods, absolute (ABFE) and relative (RBFE), trade throughput for accuracy [91, 92, 93, 94, 63, 95, 96]. Modern workflows based on the free energy perturbation theory (FEP) [97] have achieved impressive accuracy on suitable congeneric series [98, 59, 20], but remain sensitive to force fields and system preparation.

Machine learning (ML) offers a complementary path to rapid affinity estimation by learning structure–activity relationships directly from data. The early ML scoring functions used interaction fingerprints and hand-made descriptors [99, 100, 101, 102]. Sequence-based CPI / DTA models (for example, DeepDTA) encode proteins and ligands from 1D inputs to predict binding affinity [103], while more recent deep architectures, such as 3D convolutional neural networks and graph neural networks, operate more holistically on complex geometry and interaction graphs [65, 104, 105, 106, 107, 108, 24].

ML models are typically trained and evaluated on community benchmarks (for example, CASF) [109, 110]. However, strong in-benchmark performance does not guarantee generalization. Multiple analyses show that models can overfit ligand biases, struggle on out-of-distribution (OOD) targets, or even partially fit to noise [111, 112, 113, 114]. This limits their reliability in prospective campaigns and underscores the need for approaches that encode biophysical constraints, reduce dataset shortcuts, and validate on OOD benchmarks.

Compounding these challenges is data scarcity: structure-based learning ideally requires reliable affinity measurements paired with high-resolution 3D protein–ligand complexes. Although data augmentation is a mainstay in computer vision and NLP [115, 116], generating meaningful molecular data that respect stereochemistry, conformational physics, and pocket geometry remains non-trivial. However, combining ChEMBL and PDBbind through comparative complex structure and enhanced template-based modeling resulted in the BindingNet resource, comprising *ca.* 690k complexes. This substantially densifies the bioactivity landscape compared to the PDBbind alone [10, 11].

A recent advancement is Boltz-2, a co-folding foundation model that predicts complex structures and based on that binding affinity, approaching FEP-level accuracy on certain targets while running orders of magnitude faster, makes large-scale affinity ranking feasible [45]. Boltz-2's affinity module couples structural inference with potency prediction, providing a stronger supervisory signal than *post hoc* scoring and highlighting the value of unified structure–affinity modeling in end-to-end pipelines [45].

References

[1] Durairaj, J., Adeshina, Y., Cao, Z., Zhang, X., Oleinikovas, V., Duignan, T. et al. PLINDER: The protein-ligand interactions dataset and evaluation resource. *bioRxiv* (2024).

[2] Irwin, J. J., Tang, K. G., Young, J., Dandarchuluun, C., Wong, B. R., Khurelbaatar, M. et al. ZINC20—A Free Ultralarge-Scale Chemical Database for Ligand Discovery. *Journal of Chemical Information and Modeling* **60**, 6065-6073 (2020).

[3] Bolton, E. E., Chen, J., Kim, S., Han, L., He, S., Shi, W. et al. PubChem3D: a new resource for scientists. *Journal of Cheminformatics* **3**, 32 (2011).

[4] Cremer, J., Le, T., Noé, F., Clevert, D. & Schütt, K. T. PILOT: equivariant diffusion for pocket-conditioned de novo ligand generation with multi-objective guidance via importance sampling. *Chem. Sci.* **15**, 14954-14967 (2024).

[5] Levine, D. S., Shuaibi, M., Spotte-Smith, E. W. C., Taylor, M. G., Hasyim, M. R., Michel, K. et al. The Open Molecules 2025 (OMol25) Dataset, Evaluations, and Models. Preprint at `https://arxiv.org/abs/2505.08762` (2025).

[6] Tang, J., Szwajda, A., Shakyawar, S., Xu, T., Hintsanen, P., Wennerberg, K. et al. Drug–target interaction prediction with the KIBA dataset. *PLoS Computational Biology* **10**, e1003765 (2014).

[7] Davis, M. I., Hunt, J. P., Herrgard, S., Ciceri, P., Wodicka, L. M., Pallares, G. et al. Comprehensive analysis of kinase inhibitor selectivity. *Nature Biotechnology* **29**, 1046-1051 (2011).

[8] Wu, M., Xie, Z. & Zhi, D. A Folding-Docking-Affinity framework for protein-ligand binding affinity prediction. *Communications Chemistry* **8**, 108 (2025).

[9] Backenköhler, M., Groß, J., Wolf, V. & Volkamer, A. Guided Docking as a Data Generation Approach Facilitates Structure-Based Machine Learning on Kinases. *Journal of Chemical Information and Modeling* **64**, 4009-4020 (2024).

[10] Li, X., Shen, C., Zhu, H., Yang, Y., Wang, Q., Yang, J. et al. A High-Quality Data Set of Protein–Ligand Binding Interactions Via Comparative Complex Structure Modeling. *Journal of Chemical Information and Modeling* **64**, 2454-2466 (2024).

[11] Zhu, H., Li, X., Chen, B. & Huang, N. Augmented BindingNet dataset for enhanced ligand binding pose predictions using deep learning. *npj Drug Discovery* **2**, 1 (2025).

[12] Lemos, P., Beckwith, Z., Bandi, S., van Damme, M., Crivelli-Decker, J., Shields, B. J. et al. SAIR: Enabling Deep Learning for Protein-Ligand Interactions with a Synthetic Structural Dataset. *bioRxiv* (2025).

[13] Wohlwend, J., Corso, G., Passaro, S., Reveiz, M., Leidal, K., Swiderski, W. et al. Boltz-1 Democratizing Biomolecular Interaction Modeling. *bioRxiv* (2024).

[14] Buttenschoen, M., Morris, G. M. & Deane, C. M. PoseBusters: AI-based docking methods fail to generate physically valid poses or generalise to novel sequences. `http://dx.doi.org/10.1039/D3SC04185A` (2024).

[15] Eberhardt, J., Santos-Martins, D., Tillack, A. F. & Forli, S. AutoDock Vina 1.2.0: New Docking Methods, Expanded Force Field, and Python Bindings. *Journal of Chemical Information and Modeling* **61**, 3891-3898 (2021).

[16] Hu, L., Benson, M. L., Smith, R. D., Lerner, M. G. & Carlson, H. A. Binding MOAD (Mother Of All Databases). *Proteins: Structure, Function, and Bioinformatics* **60**, 333–340 (2005).

[17] Cremer, J., Irwin, R., Tibot, A., Janet, J. P., Olsson, S. & Clevert, D. FLOWR: Flow Matching for Structure-Aware de Novo, Interaction- and Fragment-Based Ligand Generation. *arXiv preprint* (2025).

[18] Bouysset, C. & Fiorucci, S. ProLIF: a library to encode molecular interactions as fingerprints. *Journal of Cheminformatics* **13**, 72 (2021).

[19] Wang, Y., Sun, K., Li, J., Guan, X., Zhang, O., Bagni, D. et al. A workflow to create a high-quality protein–ligand binding dataset for training, validation, and prediction tasks. *Digital Discovery* **4**, 1209-1220 (2025).

[20] Ross, G. A., Lu, C., Scarabelli, G. & Wang, L. The maximal and current accuracy of rigorous protein–ligand binding free energy calculations. *Communications Chemistry* **6**, 222 (2023).

[21] Yosinski, J., Clune, J., Bengio, Y. & Lipson, H. How transferable are features in deep neural networks?. In *Advances in Neural Information Processing Systems* 3320–3328 (2014).

[22] Mahajan, D., Girshick, R., Ramanathan, V., He, K., Paluri, M., Li, Y. et al. Exploring the Limits of Weakly Supervised Pretraining. In *Proceedings of the European Conference on Computer Vision (ECCV)* 181–196 (2018).

[23] McCabe, M., Régaldo-Saint Blancard, B., Parker, L. H., Ohana, R., Cranmer, M., Bietti, A. et al. Multiple Physics Pretraining for Physical Surrogate Models. In *Advances in Neural Information Processing Systems* (2023).

[24] Valsson, Ö., Warren, M. T., Deane, C. M., Magarkar, A., Morris, G. M. & Biggin, P. C. Narrowing the gap between machine learning scoring functions and free energy perturbation using augmented data. *Communications Chemistry* **8**, 41 (2025).

[25] Hsu, W., Grevtsev, S., Herz, A. M., Douglas, T., Magarkar, A. & Biggin, P. C. Can AI-Predicted Complexes Teach Machine Learning to Compute Drug Binding Affinity?. *Journal of Chemical Information and Modeling* **65**, 13051–13056 (2025).

[26] Tang, J., Szwajda, A., Shakyawar, S., Xu, T., Hintsanen, P., Wennerberg, K. et al. Making Sense of Large-Scale Kinase Inhibitor Bioactivity Data Sets: A Comparative and Integrative Analysis. *Journal of Chemical Information and Modeling* **54**, 735–743 (2014).

[27] Johnston, R. C., Yao, K., Kaplan, Z., Chelliah, M., Leswing, K., Seekins, S. et al. Epik: pK_a and Protonation State Prediction through Machine Learning. *Journal of Chemical Theory and Computation* **19**, 2380–2388 (2023).

[28] Sastry, G. M., Adzhigirey, M., Day, T., Annabhimoju, R. & Sherman, W. Protein and ligand preparation: parameters, protocols, and influence on virtual screening enrichments. *Journal of Computer-Aided Molecular Design* **27**, 221–234 (2013).

[29] Yang, Y., Yao, K., Repasky, M. P., Leswing, K., Abel, R., Shoichet, B. K. et al. Efficient Exploration of Chemical Space with Docking and Deep Learning. *Journal of Chemical Theory and Computation* **17**, 7106–7119 (2021).

[30] Ho, J., Jain, A. & Abbeel, P. Denoising Diffusion Probabilistic Models. *NeurIPS* (2020).

[31] Song, Y., Sohl-Dickstein, J., Kingma, D. P., Kumar, A., Ermon, S. & Poole, B. Score-Based Generative Modeling through Stochastic Differential Equations. *ICLR* (2021).

[32] Rombach, R., Blattmann, A., Lorenz, D., Esser, P. & Ommer, B. High-Resolution Image Synthesis with Latent Diffusion Models. In *CVPR* (2022).

[33] Lipman, Y., Chen, R. T. Q., Ben-Hamu, H., Nickel, M. & Le, M. Flow Matching for Generative Modeling. *ICLR* (2023).

[34] Liu, X., Gong, C. & Liu, Q. Flow Straight and Fast: Learning to Generate and Transfer Data with Rectified Flow. *arXiv preprint* (2022).

[35] Albergo, M. S. & Vanden-Eijnden, E. Building Normalizing Flows with Stochastic Interpolants. *ICLR* (2023).

[36] Albergo, M. S., Boffi, N. M. & Vanden-Eijnden, E. Stochastic Interpolants: A Unifying Framework for Flows and Diffusions. *arXiv preprint* (2023).

[37] Hoogeboom, E., Satorras, V. G., Vignac, C. & Welling, M. Equivariant Diffusion for Molecule Generation in 3D. In *Proceedings of the 39th International Conference on Machine Learning* 8867–8887 (2022).

[38] Vignac, C., Osman, N., Toni, L. & Frossard, P. MiDi: Mixed Graph and 3D Denoising Diffusion for Molecule Generation. In *Machine Learning and Knowledge Discovery in Databases: Research Track - European Conference, ECML PKDD 2023, Turin, Italy, September 18-22, 2023, Proceedings, Part II* 560–576 (2023).

[39] Le, T., Cremer, J., Noé, F., Clevert, D. & Schütt, K. Navigating the Design Space of Equivariant Diffusion-Based Generative Models for De Novo 3D Molecule Generation. Preprint at `https://arxiv.org/abs/2309.17296` (2023).

[40] Schneuing, A., Du, Y., Harris, C., Jamasb, A., Igashov, I., Du, W. et al. Structure-based Drug Design with Equivariant Diffusion Models. Preprint at `https://arxiv.org/abs/2210.13695` (2023).

[41] Guan, J., Qian, W. W., Peng, X., Su, Y., Peng, J. & Ma, J. 3D Equivariant Diffusion for Target-Aware Molecule Generation and Affinity Prediction. In *The Eleventh International Conference on Learning Representations* (2023).

[42] Campbell, A., Yim, J., Barzilay, R., Rainforth, T. & Jaakkola, T. Generative Flows on Discrete State-Spaces: Enabling Multimodal Flows with Applications to Protein Co-Design. Preprint at `https://arxiv.org/abs/2402.04997` (2024).

[43] Irwin, R., Tibo, A., Janet, J. P. & Olsson, S. Efficient 3D Molecular Generation with Flow Matching and Scale Optimal Transport. Preprint at `https://arxiv.org/abs/2406.07266` (2024).

[44] Abramson, J., Adler, J., Dunger, J., Evans, R., Green, T., Pritzel, A. et al. Accurate structure prediction of biomolecular interactions with AlphaFold 3. *Nature* **630**, 493-500 (2024).

[45] Passaro, S., Corso, G., Wohlwend, J., Reveiz, M., Thaler, S., Somnath, V. R. et al. Boltz-2: Towards Accurate and Efficient Binding Affinity Prediction. *bioRxiv* (2025).

[46] Shi, C., Gao, M., Wang, S., Smidt, T. & Gómez-Bombarelli, R. Learning Gradient Fields for Molecular Conformation Generation. In *ICML* (2021).

[47] Xu, M., Yu, L., Song, Y., Shi, C., Ermon, S. & Tang, J. GeoDiff: a Geometric Diffusion Model for Molecular Conformation Generation. Preprint at `https://arxiv.org/abs/2203.02923` (2022).

[48] Dunn, I. & Koes, D. R. FlowMol3: Flow Matching for 3D De Novo Small-Molecule Generation. Preprint at `https://arxiv.org/abs/2508.12629` (2025).

[49] Wang, C., Xu, S. & Wang, Y. MolCRAFT: Structure-Based Drug Design in Continuous Parameter Space. *arXiv preprint* (2024).

[50] Imrie, F., Bradley, A. R., van der Schaar, M. & Deane, C. M. Deep Generative Models for 3D Linker Design. *Journal of Chemical Information and Modeling* **60**, 1983-1995 (2020).

[51] Voloboev, S. A Review on Fragment-based De Novo 2D Molecule Generation. *arXiv preprint* (2024).

[52] Zhang, O., Huang, Y., Cheng, S., Yu, M., Zhang, X., Lin, H. et al. FragGen: towards 3D geometry reliable fragment-based molecular generation. *Chem. Sci.* **15**, 19452-19465 (2024).

[53] Lee, J., Kim, S. & Kim, W. Y. FragFM: Efficient Fragment-Based Molecular Generation via Discrete Flow Matching. *arXiv preprint* (2025).

[54] Guo, J., Knuth, F., Margreitter, C., Janet, J. P., Papadopoulos, K., Engkvist, O. et al. Link-INVENT: generative linker design with reinforcement learning. *Digital Discovery* **2**, 392-408 (2023).

[55] Igashov, I., Stärk, H., Vignac, C., Schneuing, A., Satorras, V. G., Frossard, P. et al. Equivariant 3D-conditional diffusion model for molecular linker design. *Nature Machine Intelligence* **6**, 417-427 (2024).

[56] Trott, O. & Olson, A. J. AutoDock Vina: improving the speed and accuracy of docking with a new scoring function, efficient optimization, and multithreading. *Journal of Computational Chemistry* **31**, 455–461 (2010).

[57] Friesner, R. A., Banks, J. L., Murphy, R. B., Halgren, T. A., Klicic, J. J., Mainz, D. T. et al. Glide: A New Approach for Rapid, Accurate Docking and Scoring. 1. Method and Assessment of Docking Accuracy. *Journal of Medicinal Chemistry* **47**, 1739-1749 (2004).

[58] Jones, G., Willett, P., Glen, R. C., Leach, A. R. & Taylor, R. Development and validation of a genetic algorithm for flexible docking11Edited by F. E. Cohen. *Journal of Molecular Biology* **267**, 727-748 (1997).

[59] Wang, L., Wu, Y., Deng, Y., Kim, B., Pierce, L., Krilov, G. et al. Accurate and Reliable Prediction of Relative Ligand Binding Potency in Prospective Drug Discovery by Way of a Modern Free-Energy Calculation Protocol and Force Field. *Journal of the American Chemical Society* **137**, 2695-2703 (2015).

[60] Mey, A. S. J. S., Allen, B. K., Macdonald, H. E. B., Chodera, J. D., Hahn, D. F., Kuhn, M. et al. Best Practices for Alchemical Free Energy Calculations [Article v1.0]. *Living J Comput Mol Sci* **2** (2020).

[61] Mobley, D. L. & Klimovich, P. V. Perspective: Alchemical free energy calculations for drug discovery. *The Journal of Chemical Physics* **137**, 230901 (2012).

[62] Alibay, I., Magarkar, A., Seeliger, D. & Biggin, P. C. Evaluating the use of absolute binding free energy in the fragment optimisation process. *Communications Chemistry* **5**, 105 (2022).

[63] Feng, M., Heinzelmann, G. & Gilson, M. K. Absolute binding free energy calculations improve enrichment of actives in virtual compound screening. *Scientific Reports* **12**, 13640 (2022).

[64] Ries, B., Alibay, I., Anand, N. M., Biggin, P. C. & Magarkar, A. Automated Absolute Binding Free Energy Calculation Workflow for Drug Discovery. *Journal of Chemical Information and Modeling* **64**, 5357-5364 (2024).

[65] Jiménez, J., Škalič, M., Martínez-Rosell, G. & De Fabritiis, G. K_DEEP: Protein–Ligand Absolute Binding Affinity Prediction via 3D-Convolutional Neural Networks. *Journal of Chemical Information and Modeling* **58**, 287–296 (2018).

[66] Schneuing, A., Igashov, I., Dobbelstein, A. W., Castiglione, T., Bronstein, M. M. & Correia, B. Multi-domain Distribution Learning for De Novo Drug Design. In *The Thirteenth International Conference on Learning Representations* (2025).

[67] Wang, R., Fang, X., Lu, Y., Yang, C. & Wang, S. The PDBbind database: methodologies and updates. *Journal of medicinal chemistry* **48**, 4111–4119 (2005).

[68] Gebauer, N. W. A., Gastegger, M. & Schütt, K. T. Symmetry-adapted generation of 3d point sets for the targeted discovery of molecules. Preprint at `https://arxiv.org/abs/1906.00957` (2020).

[69] Gebauer, N. W. A., Gastegger, M., Hessmann, S. S. P., Müller, K. & Schütt, K. T. Inverse design of 3d molecular structures with conditional generative neural networks. *Nature Communications* **13**, 973 (2022).

[70] Luo, Y. & Ji, S. An Autoregressive Flow Model for 3D Molecular Geometry Generation from Scratch. In *International Conference on Learning Representations* (2022).

[71] Sohl-Dickstein, J., Weiss, E., Maheswaranathan, N. & Ganguli, S. Deep unsupervised learning using nonequilibrium thermodynamics. In *International conference on machine learning* 2256–2265 (2015).

[72] Ho, J., Jain, A. & Abbeel, P. Denoising diffusion probabilistic models. *Advances in neural information processing systems* **33**, 6840–6851 (2020).

[73] Kingma, D. P., Salimans, T., Poole, B. & Ho, J. Variational Diffusion Models. In *Advances in Neural Information Processing Systems (NeurIPS)* (2021).

[74] Jing, B., Corso, G., Chang, J., Barzilay, R. & Jaakkola, T. Torsional Diffusion for Molecular Conformer Generation. Preprint at `https://arxiv.org/abs/2206.01729` (2023).

[75] Yang, Y., Zheng, S., Su, S., Zhao, C., Xu, J. & Chen, H. SyntaLinker: automatic fragment linking with deep conditional transformer neural networks. *Chem. Sci.* **11**, 8312-8322 (2020).

[76] Feng, Y., Yang, Y., Deng, W., Chen, H. & Ran, T. SyntaLinker-Hybrid: A deep learning approach for target specific drug design. *Artificial Intelligence in the Life Sciences* **2**, 100035 (2022).

[77] Hadfield, T. E., Imrie, F., Merritt, A., Birchall, K. & Deane, C. M. Incorporating Target-Specific Pharmacophoric Information into Deep Generative Models for Fragment Elaboration. *Journal of Chemical Information and Modeling* **62**, 2280-2292 (2022).

[78] Ghorbani, M., Gendelev, L., Beroza, P. & Keiser, M. J. Autoregressive fragment-based diffusion for pocket-aware ligand design. *arXiv preprint arXiv:2401.05370* (2023).

[79] Torge, J., Harris, C., Mathis, S. V. & Liò, P. DiffHopp: A Graph Diffusion Model for Novel Drug Design via Scaffold Hopping. In *ICML Workshop on Computational Biology* (2023).

[80] Yoo, K., Oertell, O., Lee, J., Lee, S. & Kang, J. TurboHopp: Accelerated Molecule Scaffold Hopping with Consistency Models. In *NeurIPS* (2024).

[81] Reymond, J., van Deursen, R., Blum, L. C. & Ruddigkeit, L. Chemical space as a source for new drugs. *MedChemComm* **1**, 30–38 (2010).

[82] Jorgensen, W. L. The Many Roles of Computation in Drug Discovery. *Science* **303**, 1813–1818 (2004).

[83] Liu, J. & Wang, R. Classification of Current Scoring Functions. *Journal of Chemical Information and Modeling* **55**, 475–482 (2015).

[84] Gilson, M. K. & Zhou, H. Calculation of Protein–Ligand Binding Affinities. *Annual Review of Biophysics and Biomolecular Structure* **36**, 21–42 (2007).

[85] Pecina, A., Fanfrlík, J., Lepšík, M. & Řezáč, J. SQM2.20: Semiempirical quantum-mechanical scoring function yields DFT-quality protein–ligand binding affinity predictions in minutes. *Nature Communications* **15**, 1127 (2024).

[86] Molani, F. & Cho, A. E. Accurate protein-ligand binding free energy estimation using QM/MM on multi-conformers predicted from classical mining minima. *Commun Chem* **7**, 247 (2024).

[87] Kollman, P. A., Massova, I., Reyes, C., Kuhn, B., Huo, S., Chong, L. et al. Calculating Structures and Free Energies of Complex Molecules: Combining Molecular Mechanics and Continuum Models. *Accounts of Chemical Research* **33**, 889–897 (2000).

[88] Homeyer, N. & Gohlke, H. Free Energy Calculations by the Molecular Mechanics Poisson–Boltzmann Surface Area Method. *Molecular Informatics* **31**, 114–122 (2012).

[89] Still, W. C., Tempczyk, A., Hawley, R. C. & Hendrickson, T. Semianalytical Treatment of Solvation for Molecular Mechanics and Dynamics. *Journal of the American Chemical Society* **112**, 6127–6129 (1990).

[90] Gohlke, H. & Case, D. A. Converging Free Energy Estimates: MM-PB(GB)SA Studies on the Protein–Protein Complex Ras–Raf. *Journal of Computational Chemistry* **25**, 238–250 (2004).

[91] Aldeghi, M., Heifetz, A., Bodkin, M. J., Knapp, S. & Biggin, P. C. Accurate calculation of the absolute free energy of binding for drug molecules. *Chem. Sci.* **7**, 207-218 (2016).

[92] Boresch, S., Tettinger, F., Leitgeb, M. & Karplus, M. Absolute Binding Free Energies: A Quantitative Approach for Their Calculation. *The Journal of Physical Chemistry B* **107**, 9535–9551 (2003).

[93] Gilson, M. K., Given, J. A., Bush, B. L. & McCammon, J. A. The Statistical-Thermodynamic Basis for Computation of Binding Affinities: A Critical Review. *Biophysical Journal* **72**, 1047–1069 (1997).

[94] Fu, H., Chen, H., Blazhynska, M., Goulard Coderc de Lacam, E., Szczepaniak, F., Pavlova, A. et al. Accurate determination of protein:ligand standard binding free energies from molecular dynamics simulations. *Nature Protocols* **17**, 1114-1141 (2022).

[95] Cournia, Z., Allen, B. & Sherman, W. Relative Binding Free Energy Calculations in Drug Discovery: Recent Advances and Practical Considerations. *Journal of Chemical Information and Modeling* **57**, 2911–2937 (2017).

[96] Ross, G. A., Lu, C., Scarabelli, G., Albanese, S. K., Houang, E., Abel, R. et al. The maximal and current accuracy of rigorous protein-ligand binding free energy calculations. *Communications Chemistry* **6**, 222 (2023).

[97] Zwanzig, R. W. High-Temperature Equation of State by a Perturbation Method. I. Nonpolar Gases. *The Journal of Chemical Physics* **22**, 1420–1426 (1954).

[98] Abel, R., Wang, L., Harder, E. D., Berne, B. J. & Friesner, R. A. Advancing Drug Discovery through Enhanced Free Energy Calculations. *Accounts of Chemical Research* **50**, 1625–1632 (2017).

[99] Ballester, P. J. & Mitchell, J. B. O. A machine learning approach to predicting protein–ligand binding affinity with applications to molecular docking. *Bioinformatics* **26**, 1169–1175 (2010).

[100] Wójcikowski, M., Kukiełka, M., Stepniewska-Dziubińska, M. M. & Siedlecki, P. Development of a protein–ligand extended connectivity (PLEC) fingerprint and its application for binding affinity predictions. *Bioinformatics* **35**, 1334–1341 (2019).

[101] Kundu, I., Paul, G. & Banerjee, R. A machine learning approach towards the prediction of protein–ligand binding affinity based on fundamental molecular properties. *RSC Advances* **8**, 12127–12137 (2018).

[102] Boyles, F., Deane, C. M. & Morris, G. M. Learning from the ligand: using ligand-based features to improve binding affinity prediction. *Bioinformatics* **36**, 758–764 (2020).

[103] Öztürk, H., Özgür, A. & Ozkirimli, E. DeepDTA: Deep Drug–Target Binding Affinity Prediction. *Bioinformatics* **34**, i821–i829 (2018).

[104] Jiang, D., Hsieh, C., Wang, Z., Kang, Y., Wang, J., Liao, B. et al. InteractionGraphNet: A Novel and Efficient Deep Graph Representation Learning Framework for Accurate Protein–Ligand Interaction Predictions. *Journal of Medicinal Chemistry* **64**, 18209–18232 (2021).

[105] Karlov, D. S., Sosnin, S., Fedorov, M. V. & Popov, P. graphDelta: MPNN Scoring Function for the Affinity Prediction of Protein–Ligand Complexes. *ACS Omega* **5**, 5150–5159 (2020).

[106] Nguyen, T., Le, T. M., Quinn, T. P., Nguyen, T. & Venkatesh, S. GraphDTA: Predicting Drug–Target Binding Affinity with Graph Neural Networks. *Bioinformatics* **37**, 1140–1147 (2021).

[107] Li, S., Zhou, J., Xu, T., Huang, L., Wang, F., Xiong, H. et al. Structure-Aware Interactive Graph Neural Networks for the Prediction of Protein–Ligand Binding Affinity. In *Proceedings of the 27th ACM SIGKDD Conference on Knowledge Discovery and Data Mining* 975–985 (2021).

[108] Meli, R., Morris, G. M. & Biggin, P. C. Scoring Functions for Protein–Ligand Binding Affinity Prediction Using Structure-Based Deep Learning: A Review. *Frontiers in Bioinformatics* **2**, 885983 (2022).

[109] Li, Y., Su, M., Liu, Z., Li, J., Liu, J., Han, L. et al. Assessing protein–ligand interaction scoring functions with the CASF-2013 benchmark. *Nature Protocols* **13**, 666–680 (2018).

[110] Su, M., Yang, Q., Du, Y., Feng, G., Liu, Z., Li, Y. et al. Comparative Assessment of Scoring Functions: The CASF-2016 Update. *Journal of Chemical Information and Modeling* **59**, 895–913 (2019).

[111] Volkov, M., Turk, J. A., Drizard, N., Martin, N., Hoffmann, B., Stahl, M. et al. On the Frustration to Predict Binding Affinities from Protein–Ligand Structures with Deep Neural Networks. *Journal of Medicinal Chemistry* **65**, 7946–7958 (2022).

[112] Scantlebury, J., Vost, L., Carbery, A., Hadfield, T. E., Turnbull, O. M., Brown, N. et al. A Small Step Toward Generalizability: Training a Machine Learning Scoring Function for Structure-Based Virtual Screening. *Journal of Chemical Information and Modeling* **63**, 2960–2974 (2023).

[113] Yang, J., Shen, C. & Huang, N. Predicting or Pretending: Artificial Intelligence for Protein–Ligand Interactions Lack of Sufficiently Large and Unbiased Datasets. *Frontiers in Pharmacology* **11**, 69 (2020).

[114] Crusius, D., Cipcigan, F. & Biggin, P. C. Are we fitting data or noise? Analysing the predictive power of commonly used datasets in drug-, materials-, and molecular-discovery. *Faraday Discussions* **256**, 304–321 (2025).

[115] Paulin, G. & Ivašić-Kos, M. Review and analysis of synthetic dataset generation methods and techniques for application in computer vision. *Artificial Intelligence Review* **56**, 9221–9265 (2023).

[116] Pellicer, L. F. A. O., Ferreira, T. M. & Costa, A. H. R. Data augmentation techniques in natural language processing. *Applied Soft Computing* **132**, 109803 (2023).

[117] Ramírez, S. FastAPI. `https://fastapi.tiangolo.com` (2018).

[118] Rego, N. & Koes, D. 3Dmol.js: molecular visualization with WebGL. *Bioinformatics* **31**, 1322–1324 (2015).

[119] Landrum, G. et al. RDKit: Open-Source Cheminformatics Software. `https://www.rdkit.org` (2006).

[120] Plotly Technologies Inc. Plotly.js—Open Source Graphing Library for JavaScript. `https://plotly.com/javascript/` (2015).

[121] van der Maaten, L. & Hinton, G. Visualizing Data using t-SNE. *Journal of Machine Learning Research* **9**, 2579–2605 (2008).

[122] McInnes, L., Healy, J. & Melville, J. UMAP: Uniform Manifold Approximation and Projection for Dimension Reduction. Preprint at `https://arxiv.org/abs/1802.03426` (2018).

[123] Rogers, D. & Hahn, M. Extended-Connectivity Fingerprints. *Journal of Chemical Information and Modeling* **50**, 742–754 (2010).

[124] Bannwarth, C., Ehlert, S. & Grimme, S. GFN2-xTB—An Accurate and Broadly Parametrized Self-Consistent Tight-Binding Quantum Chemical Method with Multipole Electrostatics and Density-Dependent Dispersion Contributions. *Journal of Chemical Theory and Computation* **15**, 1652–1671 (2019).

[125] Hu, E. J., Shen, Y., Wallis, P., Allen-Zhu, Z., Li, Y., Wang, S. et al. LoRA: Low-Rank Adaptation of Large Language Models. Preprint at `https://arxiv.org/abs/2106.09685` (2021).
