# Supplementary material for: FLOWR.ROOT – A flow matching-based foundation model for joint multi-purpose structure-aware 3D ligand generation and affinity prediction
Source: Nat Commun. 2026 Jul 6;17:5883. doi: 10.1038/s41467-026-74130-9 (PMC13338463; doi:10.1038/s41467-026-74130-9)
Supplement: Supplementary file 2 — Transparent Peer Review file [file 41467_2026_74130_MOESM2_ESM.pdf]

nature portfolio

Peer Review File

FLOWR.root: A flow matching based foundation model for joint multi-purpose structure-aware 3D ligand generation and affinity prediction

Corresponding Author: Dr Julian Cremer

This file contains all reviewer reports in order by version, followed by all author rebuttals in order by version.

Version 0:

Reviewer comments:

Reviewer #1

(Remarks to the Author)
The authors present FLOWR.ROOT, a multi-fidelity SE(3)-equivariant generative and affinity-prediction framework trained on a heterogeneuos collection of structural and biochemical datasets. Although the manuscript is ambitious, several fundamental scientific and methodological problems undermine the reliability of the results and novelty of the scientific point and prevent the work from meeting the standards of Nature Communications.
Some major points are listed below:
1. The use of heterogeneous affinity labels (IC50, Ki, Kd, EC50) as equivalent training targets is not scientifically justified. These measurements arise from fundamentally different assay modalities, kinetic regimes, protein constructs, and experimental conditions. Treating them as interchangeable and reporting aggregated correlations across them cannot provide meaningful interpretation of the results. Without normalization or model separation across assay types, the reported performance metrics cannot be considered reliable indicators.
2. Datasets such as SAIR, BindingNet, KIBA-3D, Davis-3D, and Plinder contain computationally folded pockets, docking-based placements, and template-based complexes. These structures often deviate significantly from physically realistic binding modes, but the model treats them equivalently to curated experimental structures. No ablation analysis is provided to quantify how these inaccuracies influence the generative or affinity components of the model, raising concerns that performance is driven by dataset-specific shortcuts rather than genuine physical signal.
3. The manuscript refers to FEP+/OpenFEE benchmarks but does not present detailed, quantitative comparisons. The quantum-chemical validation relies only on GFN2-xTB, which is too low in accuracy for protein-ligand energetics.
4.There is no controlled demonstration showing how inclusion of the affinity head modifies ligand placement, improves pose quality, or affects structural sampling. In the absence of ablation studies, it is unclear whether the generative model benefits from affinity supervision or whether the claim is largely conceptual.
5. Long-range electrostatics, water-mediated interactions, distal hydrogen bonds, and entropic effects are all excluded by such a cutoff. It is therefore difficult to reconcile the claimed affinity prediction accuracy with the limited physical information provided to the model.
6. CrossDocked2020 and BindingNet involve shared protein backbones and docking templates. Without explicit guarantees that protein families, templates, or backbone conformations do not overlap between training and test sets, reported improvements in pose generation and affinity prediction may be artificially inflated.
7. The authors use residue embeddings and local environmental vectors extracted from a truncated radius sphere. However, when pocket atoms at different distances are masked or omitted, the equivariance assumptions of the model no longer strictly hold. The paper does not analyze the conditions under which this approximation is valid, nor whether equivariance is maintained for irregular pocket geometries.
8. The core architectural ideas: equivariant flows, pocket conditioning, and molecular generation, are very close to those in FLOWR, FLOWR.MULTI, and PILOT. The primary differences seem to involve larger datasets and joint affinity training. They do not constitute a sufficiently novel conceptual contributuon for Nature Communications.

Minor:
Several figures lack statistical uncertainty or variance estimates.

(Remarks on code availability)

Reviewer #2

(Remarks to the Author)
The authors present a foundation model for ligand generation with binding affinity prediction and confidence estimation. The model addresses a long-standing challenge of drug discovery and related fields. Therefore the paper represents an important advance, and is potentially interesting.

I have a few comments for the authors to address

1. The authors need to demonstrate and articulate more clearly how the new foundation model is distinct and/or superior to earlier approaches.
Specifically:
There are an number of other foundation models (some of which are included in the _Results_ section of the manuscript) which aim to do similar things -- ligand generation, binding affinity prediction etc. They typically have robust zero-shot protein–ligand docking and few-shot binding affinity capabilities, but still require finetuning to be fully applicable in a drug discovery setting.
The foundation model presented in this manuscript appears to be relatively similar, albeit with superior performance across different dimensions.
The authors should articulate more clearly if their model is (i) similar-but-slightly-better; or (ii) distinct in some fundamentally different way.
If it's (i), then the authors need to provide a deeper discussion of what enabled the superior performance (e.g. training data used, data and evidence of how the model learns, evidence of scaling/speed etc.)
And if it's (ii), the we need a clearer articulation of what the fundamental differences are.

2. Beyond the data shown in the _Results_ section (which are self-reported) is there any way to demonstrate performance in an external and independent setting?
For example by participating in external competitions, such as the CACHE challenge.
If this is not possible in a reasonable timeframe, the authors should commit to participating in future such competitions.

3. In many places, the manuscript reads a bit like a PhD thesis, rather than an academic or industrial publication.
To make is suitable as a publication, I would recommend restructuring the manuscript extensively.
Specifically:
- Section 1 (_Introduction_) and Section 2 (_Related works_) read a bit like a review. Both should be substantially condensed.
- Section 3 (_Datasets_) has an extensive list and description of datasets. This should be shortened and/or moved to a supplementary file
- Section 4 (_Methods_) should be moved to a supplementary file; the main manusript could include a condensed version of the methods
- Section 5 (_Results_) is of course the most important. but at the moment, it's 12 pages long which feels unwieldy. Could this be shorted and sharpened? Less-critical results could be moved to supplemental file.

4. I note that some of authors are affiliated with a pharmaceutical company; other authors are based in academic institutions. Given the subject matter of the article (drug discovery), the manuscript therefore needs to include a declaration of potential conflicts of interest. All funding sources, industrial as well as academic, need to be fully acknowledged.

5. In the _Datasets_ section and in the _Results_ section, the authors talk about many similar datasets.
The authors need to describe more clearly how they avoided data leakage -- otherwise the superior performance reported in the manuscript may not be believed.

(Remarks on code availability)
n/a

Reviewer #3

(Remarks to the Author)
General Comments: The authors present an impressively large-scale training pipeline, integrating diverse datasets such as BindingNet, SAIR, and SPINDR. This data-driven strategy aligns well with current trends in deep learning for drug

discovery. Furthermore, the application of importance sampling for property steering demonstrates the model's potential flexibility in practical drug design scenarios. However, there are several concerns regarding the validation of the joint training objective, the generalization capabilities of the model, and the rigor of the evaluation metrics.

Major Points:

1. Lack of Ablation Studies on the Affinity Head: While the joint training of structure generation and affinity prediction is a core contribution, the manuscript lacks a controlled ablation study to quantify the specific contribution of the affinity head to the quality of structure generation. It is unclear whether the performance gains are derived from the auxiliary affinity supervision or simply from the massive scale of the training data. A comparison between a model trained solely on structure generation versus the joint model (controlled for dataset size) is necessary to validate the architectural benefit.

2. Generalization vs. Memorization: There is a concern regarding whether the model is genuinely "predicting" interactions or merely "memorizing" the training distribution. Section 5.6 acknowledges that the model exhibits negligible correlation on in-house data without fine-tuning, which hints at poor zero-shot generalization. To demonstrate true utility, the authors should evaluate:
2.1 The novelty of generated molecules (e.g., similarity distributions relative to the training set).
2.2 The model’s ability to generate valid, high-quality binders for out-of-distribution (OOD) targets without reliance on project-specific fine-tuning.

3. Diversity Analysis under Affinity Steering: In Section 5.4, the authors discuss affinity steering via importance sampling. However, an analysis of internal diversity is missing. As the guidance strength increases, there is a significant risk of mode collapse, where the model may converge on a limited number of high-scoring but repetitive structures. The authors should provide metrics (e.g., IntDiv or Uniqueness) plotted against steering intensity to demonstrate that the model maintains chemical diversity while optimizing for affinity.

4. Insufficient Benchmarking in Section 5.4: In the affinity prediction evaluation on SPINDR (Section 5.4), the comparison is limited to the pre-trained FLOWR.ROOT versus the non-pre-trained base model. This only proves the value of pre-training. To demonstrate the superiority of the Flow Matching architecture itself, the authors should compare their approach against other state-of-the-art affinity prediction architectures (e.g., GNNs or Transformers) on this specific benchmark.

5. Incomplete Validation of "Valid" Generation (Section 5.4): The conclusion that the model can be biased toward higher affinity "without sacrificing chemical or geometric validity" is not fully supported by the data.
5.1 Figure 4 shows that the SA-score (Synthetic Accessibility) actually degrades (shifts higher) with guidance6.
5.2 Crucially, geometric validity metrics—such as Strain Energy and Vina Scores—are missing for the steered samples. It is vital to prove that forcing high-affinity scores does not result in physically unrealistic or highly strained conformations.

Minor Points:

6. Typo in Section 4.2:
The text states, "Both S and P are stacked to the existing pairwise message tensor..."7. However, based on the preceding equations, the cross-product tensor is defined as C, not P. Please correct this variable name.

7. Interpretation of Figure Density (Section 5.5): The statement, "The denser regions... represent higher prediction frequencies, indicating where the model performs best," is logically flawed. High density in a scatter plot typically correlates with the bulk of the training data distribution (data bias) rather than the intrinsic accuracy of the model. "Best performance" should be defined by prediction error (RMSE/MAE) across the dynamic range, not by the frequency of predictions in the mean range. Please rephrase this interpretation.

8. Misleading Metrics in Figure 9 (Top Left): The reviewer notes significant confusion regarding the metrics box presented in Figure 9 (Top Left), which reports an RMSE of 25.06 kcal/mol. This plot compares the QM binding energies of CLK3 (off-target) versus CK2α (on-target) to demonstrate selectivity. In this context, a large deviation from the identity line (high RMSE) is the intended outcome (selectivity), not an error. Labeling this as "RMSE" suggests poor performance and is highly misleading. The authors should remove these regression metrics or strictly contextualize them as a selectivity measurement.

(Remarks on code availability)
The code executes successfully, outputting the molecular conformations and their binding affinities.

Reviewer #4

(Remarks to the Author)
I co-reviewed this manuscript with one of the reviewers who provided the listed reports. This is part of the Nature Communications initiative to facilitate training in peer review and to provide appropriate recognition for Early Career Researchers who co-review manuscripts.

(Remarks on code availability)
The code executes successfully, outputting the molecular conformations and their binding affinities.

Version 1:

Reviewer comments:

Reviewer #1

(Remarks to the Author)
The manuscript has improved in clarity and organization following revision. However, several central methodological concerns remain unresolved. These issues directly affect the core claims of the work, and the revisions do not provide sufficient evidence to justify the main conclusions.
1. The manuscript presents a joint structure-affinity architecture, yet no controlled ablation study has been provided to determine whether the affinity head meaningfully impacts structural generation. A structure-only model should be trained under identical condition (same data, backbone, and hyperparameters) and directly compared to the joint model. Structural quality, diversity, stability across seeds, and convergence behavior should be evaluated. Without this comparison, the architectural benefit of joint training remains unproved and not convincing.
2. The model is trained on heterogeneous data, including computationally generated poses. The robustness of this strategy is not empirically demonstrated. A sensitivity analysis is needed. This could include retraining without lower-fidelity datasets or reporting performance stratified by data source. Without such analysis, it is unclear whether performance gains reflect architectural improvements or simply data scale and heterogeneity. For a foundation-model claim, robustness to data quality must be demonstrated.
3. The authors acknowledges likely overlap between training data and the FEP+/OpenFE benchmark. In this case, superiority claims must be supported with quantitative overlap analysis (ligand and protein similarity), or the interpretation of these results must be tempered. If overlap exists, the results should not be framed as evidence of broad generalization.
4. The manuscript positions FLOWR.ROOT as a foundation model. However, the results on proprietary datasets show that zero-shot generalization fails and that meaningful performance requires project-specific fine-tuning. While fine-tuning is common practice, this behavior does not by itself demonstrate foundation-level transfer capability. The manuscript should clarify this positioning and avoid overstating generalization claims unless additional evidence of cross-domain robustness is provided.

(Remarks on code availability)

Reviewer #2

(Remarks to the Author)
I thank the authors for addressing the points raised in a comprehensive manner.
Especially the revised structure of the manuscript works well.

Two last points, for you to consider:
1. The revised introduction section is a little dense, with very complex terminology. I would recommend, in the interest of general readability, to simplify the words a bit. Maybe some language editing may be helpful.
2. Figure 1 is also very dense. Would recommend to simplify.

(Remarks on code availability)
n/a

Reviewer #3

(Remarks to the Author)
My comments have been addressed reasonably well.

(Remarks on code availability)

Reviewer #4

(Remarks to the Author)
I co-reviewed this manuscript with one of the reviewers who provided the listed reports. This is part of the Nature Communications initiative to facilitate training in peer review and to provide appropriate recognition for Early Career Researchers who co-review manuscripts.

(Remarks on code availability)

Version 2:

Reviewer comments:

Reviewer #1

(Remarks to the Author)
The revised manuscript is clearer and better organized, and the additional explanations of the framework and training procedure are appreciated. While the manuscript has improved in clarity, the key methodological novelty concerns raised in the previous round remain still only partially addressed. Here are the major points:
1. The most important issue is the lack of a controlled validation of the joint structure–affinity architecture. While the authors clarify that they do not claim the affinity head improves structural generation, this does not address whether the joint formulation is necessary, or whether it introduces any interaction between tasks. The current arguments rely on indirect evidence and comparisons that do not isolate the role of the affinity head. Even a reduced-scale or proxy ablation would be important to justify this central design choice.
2. The manuscript positions the method as a unified or foundation-level framework, but the current evidence suggests that the work is primarily an integration of existing components rather than a clearly demonstrated conceptual advance. It remains unclear what new capability arises specifically from the joint formulation, and how it compares to a modular pipeline combining generation with external scoring.

Overall, while the work is technically solid, addressing these points would be important to fully support the scope of the claims.

(Remarks on code availability)

Reviewer #2

(Remarks to the Author)
I would like to thank the authors for addressing the points I raised -- no further comments from my side.

(Remarks on code availability)
n/a

Open Access This Peer Review File is licensed under a Creative Commons Attribution 4.0 International License, which permits use, sharing, adaptation, distribution and reproduction in any medium or format, as long as you give appropriate credit to the original author(s) and the source, provide a link to the Creative Commons license, and indicate if changes were made.
In cases where reviewers are anonymous, credit should be given to 'Anonymous Referee' and the source.
The images or other third party material in this Peer Review File are included in the article's Creative Commons license, unless indicated otherwise in a credit line to the material. If material is not included in the article's Creative Commons

license and your intended use is not permitted by statutory regulation or exceeds the permitted use, you will need to obtain permission directly from the copyright holder.
To view a copy of this license, visit https://creativecommons.org/licenses/by/4.0/

Rebuttal Letter

FLOWR.root: A flow matching based foundation model for joint multi-purpose structure-aware 3D ligand generation and affinity prediction

Manuscript ID: NCOMMS-25-95570-T

Nature Communications

Dear Reviewers,

Thank you for the constructive feedback pro-vided by the reviewers. We have carefully addressed each comment and have made substantialrevisions to improve the clarity, rigor, and presentation of our work. Below, we provide point-by-point responses to all reviewer comments. Text additions to the revised manuscript are indicatedin blue.

Sincerely,
The Authors

Reviewer #1

General Assessment

The authors present FLOWR.ROOT, a multi-fidelity SE(3)-equivariant generative and affinity-prediction framework trained on a heterogeneous collection of structural and biochemical datasets. Although the manuscript is ambitious, several fundamental scientific and methodological problems undermine the reliability of the results and novelty of the scientific point and prevent the work from meeting the standards of Nature Communications.

Major Comments

Comment 1.1 – Heterogeneous Affinity Labels

The use of heterogeneous affinity labels (IC50, Ki, Kd, EC50) as equivalent training targets is not scientifically justified. These measurements arise from fundamentally different assay modalities, kinetic regimes, protein constructs, and experimental conditions. Treating them as interchangeable and reporting aggregated correlations across them cannot provide meaningful interpretation of the results. Without normalization or model separation across assay types, the reported performance metrics cannot be considered reliable indicators.

Response:

We appreciate the reviewer's attention to this methodological consideration. The concern appears to be based on a misunderstanding of our approach. FLOWR.ROOT employs **separate prediction heads** for each potency/affinity type (pIC_{50}, pK_i, pK_d, pEC_{50}), with each head trained exclusively on its corresponding data type. This design explicitly avoids treating heterogeneous affinity labels as interchangeable and ensures that the model learns the specific characteristics associated with each measurement type. This is, to the best of our knowledge, in contrast to most, if not all, prior work in ML-based affinity prediction, where indeed an aggregated affinity output, often referred to as pK, is trained–most recent examples are AEV-PLIG [Valsson et al., 2025] and Boltz-2 [Passaro et al., 2025].

Precisely because these measurements arise from different assay modalities and experimental conditions, we opted to implement task-specific heads rather than a unified affinity prediction target. While IC_{50} and K_i are related through the Cheng-Prusoff equation, and K_d relates to K_i via thermodynamic relationships, these quantities are not directly interchangeable and our architecture reflects this understanding.

Regarding the FEP+/OpenFE benchmark evaluation where we report aggregated predictions: this follows the established convention used by Boltz-2, AEV-PLIG, and other state-of-the-art models, enabling direct comparison. Importantly, the benchmark dataset is annotated with ΔG values without specification of the underlying measurement type, making aggregation the standard approach. For the evaluation on the HIQBIND test set, however, we explicitly treat all potency and affinity labels separately as provided by the data and show all correlations (Section 2.4, Figure 2). When evaluating FLOWR.ROOT on project-specific data (Section 2.6 and 2.7), we finetune and evaluate using the provided experimental affinity type (pIC_{50} and pEC_{50}), representing the typical drug discovery scenario.

To improve clarity, we revised Section 2.1 and the Supplementary Information—Methods of the manuscript to explicitly describe how different affinity data types are used during training.

Comment 1.2 – Computationally Generated Structures

Datasets such as SAIR, BindingNet, KIBA-3D, Davis-3D, and Plinder contain computationally folded pockets, docking-based placements, and template-based complexes. These structures often deviate significantly from physically realistic binding modes, but the model treats them equivalently to curated experimental structures. No ablation analysis is provided to quantify how these inaccuracies influence the generative or affinity components of the model, raising concerns that performance is driven by dataset-specific shortcuts rather than genuine physical signal.

Response:

We appreciate this comment but must clarify a central aspect of our methodology. FLOWR.ROOT explicitly **does not** treat computationally generated and experimental structures equivalently. Our multi-stage training paradigm is specifically designed to leverage data of varying fidelity in a principled manner:

- **Stage 1 (Pre-training):** Computationally generated datasets (SAIR, BindingNet, KIBA-3D, Davis-3D, Kinodata-3D) are classified as lower- to mid-fidelity data, used

to establish broad chemical space coverage and foundational structural understanding, accompanied by higher-fidelity (co-crystal) datasets like Plinder and Binding-Moad.

- **Stage 2 (High-fidelity training):** The model is subsequently refined on curated experimental datasets (SPINDR, HiQBind), which provide higher-quality structural and affinity information.
- **Stage 3 (Domain adaptation):** Project-specific finetuning on dense, high-quality SAR data captures subtle structure-activity patterns.

We note that Plinder is a curated co-crystal dataset derived from the PDB, containing experimental structures rather than computationally generated complexes. Also, as described in detail in Supplementary Information (Datasets), we curated all datasets using a comprehensive LigPrep and PrepWizard pipeline.

Further, evidence supporting the effectiveness of this multi-fidelity approach is provided in Section 2.4: the pre-trained FLOWR.ROOT model achieves significantly better affinity prediction compared to the base model trained solely on HiQBind without pre-training. This improvement demonstrates that lower-fidelity data contributes to learning more generalizable representations, while high-fidelity data sharpens prediction accuracy. This finding aligns with prior work demonstrating the benefits of pre-training on augmented data (e.g., AEV-PLIG). Please note, that pre-training is done on coherent Plinder-based splits avoiding data leakage (Section 2).

Furthermore, the project-specific evaluation (Section 2.6), which we extended with three additional in-house studies in the revised manuscript, demonstrates that genuine physical signal is captured through finetuning on high-fidelity, project-specific data, where LoRA-finetuned FLOWR.ROOT significantly outperforms Boltz-2. We substantially increased Section 2.6, and also added Section 2.7 to the manuscript elaborating LoRA-finetuning on a public dataset (PDE10A).

Comment 1.3 – FEP+ and QM Validation

The manuscript refers to FEP+/OpenFEE benchmarks but does not present detailed, quantitative comparisons. The quantum-chemical validation relies only on GFN2-xTB, which is too low in accuracy for protein-ligand energetics.

Response:

We appreciate this comment but must clarify a misunderstanding. The FEP+/OpenFE benchmarks provide an evaluation of affinity prediction performance comparing FLOWR.ROOT with physics- as well as most recent state-of-the-art ML-based methods (Section 2.5). This evaluation is independent from the quantum mechanical evaluation we performed on selected targets showcasing the scaffold elaboration and affinity prediction capabilities of FLOWR.ROOT (Section 2.8).

FEP+/OpenFE Benchmarks: Detailed quantitative comparisons are provided in Section 2.5, Figure 4, including correlation plots, RMSE, RAE, R^2, Kendall τ, and Pearson correlation coefficients. FLOWR.ROOT outperforms physics-based methods (OpenFE, FEP+) and ML-based models (AEV-PLIG, Boltz-2) across most metrics while operating orders of magnitude faster.

Quantum-Mechanical Validation: We address the concern regarding GFN2-xTB accuracy for non-covalent protein-ligand binding energetics. Several considerations support

its suitability for this application:

1. **Validation in literature:** The SQM2.20 [Pecina et al., 2024] method demonstrated strong correlations between semi-empirical QM-derived binding energies and experimental data for multiple targets including BACE1, which we also investigate. Notably, such correlations were not observed with force-field approaches or contemporary ML scoring functions. This directly motivates the present work, as FLOWR.ROOT indeed shows strong correlation between ML-predicted affinities and a quantum-mechanical scoring function. In addition, recent work has shown that semi-empirical QM scoring can reach accuracies comparable to Free Energy Perturbation (FEP) calculations [Jalaie et al., 2025], supporting the use of semi-empirical QM methods for ligand ranking in protein binding sites.
2. **Parameterization for non-covalent interactions:** GFN2-xTB was explicitly parameterized using non-covalent interaction datasets to reproduce interaction energies across biologically relevant chemical environments. It employs the D4 dispersion, the most recent generation of Grimme's dispersion correction and an updated electrostatics model [Bannwarth et al., 2019]. Thus, the GFN2-xTB formalism is particularly suited to the description of non-bonded interactions, which dominate protein–ligand binding energetics. More generally, DFTB-based approaches have been shown in multiple studies to provide reliable reference data for biological systems [Zhang et al., 2025, Elstner, 2006, Kubillus et al., 2015, Gaus et al., 2014b,a, Cui and Elstner, 2014]. The electrostatics model employed in GFN2-xTB is more modern and complete than those used in several semi-empirical methods that have still been successfully applied to protein–ligand complexes, such as PM6-D3H4X [Stewart, 2007, Řezáč and Hobza, 2012], which underlies SQM2.20 [Pecina et al., 2024].
3. **Prior validation and practical necessity:** We have previously benchmarked GFN2-xTB to protein–ligand complexes and observed good agreement with experimentally measured relative binding enthalpies, which are the primary quantities extracted in the present work. This has been demonstrated across several studies focused on structure–activity relationships and binding selectivity (see, for example, [Grygier et al., 2023, Góral et al., 2024, Nowacki et al., 2025]). In particular, in Grygier et al. [2023], GFN2-xTB results were benchmarked against higher-level DLPNO-CCSD(T) calculations, providing explicit validation of the semi-empirical approach for these systems. We further highlight a recent study by Grygier et al. [2025], in which QM calculations based on crystallographic structures closely reproduced relative binding enthalpies obtained from ITC experiments. This work also demonstrated that treating large portions of the protein quantum mechanically is critical for accurately reproducing experimental trends. The QM regions in those calculations comprised more than 4000 atoms, and in some cases exceeded 5000 atoms, which makes higher-level DFT approaches computationally impractical, even when using linear-scaling methods. In particular, distal protein regions included in the largest QM systems were shown to influence protein–ligand electrostatic interactions, which in some cases played a significant role for binding overall. This supports the use of large QM regions as employed in the benchmarking of FLOWR.ROOT. Finally, we note that large QM regions are essential for reliably capturing binding energetics and selectivity effects in closely related targets. DYRK1A and DYRK1B are highly homologous kinases (over 85% sequence identity), and treating only small binding-site regions, as required for higher-level DFT calculations, would likely be insufficient to distinguish relative affinities or rationalize selectivity trends.

We emphasize that the QM calculations serve as an independent validation of predicted affinity trends rather than absolute binding energies. The observed correlations between FLOWR.ROOT-predicted affinities and QM binding energies (e.g., $R^2 = 0.81$ for ERα, Pearson $= 0.90$) provide evidence that the model captures physically meaningful structure-activity relationships. Importantly, QM calculations underline our findings that FLOWR.ROOT-predicted structures are low-strained and show high pose accuracy without requiring any expensive post-relaxation in contrast to prior models yielding favorable QM binding energies.
To clarify these points, we have expanded the manuscript to include a more detailed justification of the suitability of GFN2-xTB for the present application and added additional supporting references (Supplementary Information—Methods).

Comment 1.4 – Ablation of Affinity Head

There is no controlled demonstration showing how inclusion of the affinity head modifies ligand placement, improves pose quality, or affects structural sampling. In the absence of ablation studies, it is unclear whether the generative model benefits from affinity supervision or whether the claim is largely conceptual.

Response:

We wish to clarify that **we do not claim** that the affinity head improves pose quality or structural sampling. The structural generation and affinity prediction components serve distinct, complementary purposes:

- The **structure head** generates geometrically valid ligand conformations within the protein pocket enabling high structural and pose quality
- The **affinity head** provides potency and affinity estimates enabling: (1) in-distribution evaluation of generated structures, (2) inference-time steering toward higher-affinity compounds via importance sampling.
- Effective joint structure-affinity domain adaptation via LoRA-finetuning to unseen structure–activity landscapes.
- Multi-target selectivity via joint structure and affinity prediction
- Fragment-based elaboration combining conditional structure with affinity prediction.

One of the primary benefits of joint training is enabling coherent, in-distribution property-guided generation during inference, as demonstrated in Section 2.4 (Figure 3). Without the jointly trained affinity head, importance sampling-based steering would require external scoring functions, introducing not only substantial additional computational overhead, but also distribution mismatch potentially enforcing higher failure modes on the structure predictions (Section 2.4). Importantly, joint learning also enables to effectively finetune the model on unseen structure–activity landscapes, e.g. via LoRA (Section 2.6 and 2.7). In addition, a joint structure and affinity prediction model can be used to run multi-target selectivity campaigns, as well as fragment-conditional ligand generation with affinity prediction and guidance for effective local chemical space exploration (Section 2.8 and 2.9).

Comment 1.5 – Cutoff Limitations

Long-range electrostatics, water-mediated interactions, distal hydrogen bonds, and entropic effects are all excluded by such a cutoff. It is therefore difficult to reconcile the claimed affinity prediction accuracy with the limited physical information provided to the model.

Response:

We thank the reviewer for raising this consideration, but want to clarify that each mentioned interaction type carries it's own system-dependent complexity hindering a general statement about cutoff-based limitations. In addition, it is not quite clear which exact cutoff the reviewer is referring to, but assume the 7Å we've chosen to cut the protein pocket around given reference ligands.

Long-range electrostatics: The impact of long-range electrostatics is highly system-dependent and primarily determined by ligand charge. For neutral ligands, sensitivity to long-range electrostatics is minimal. Our analysis of axitinib (neutral) binding to BCR-ABL1 showed binding energy differences of approximately 2 kcal/mol between systems of 1819 and 3451 atoms, decreasing to <0.5 kcal/mol for larger systems (**We are working on the manuscript. Nothing to cite yet. I could upload to biorxiv though**). For charged ligands (e.g., AZ191 binding to DYRK1A/DYRK1B [Grygier et al., 2025]), larger effects are observed but with partial cancellation. The 7 Å cutoff represents a practical compromise between computational tractability and interaction coverage.

Distal hydrogen bonds: Hydrogen bond energetics decay rapidly with distance, effectively vanishing beyond 5.5–6 Å [Řezáč and Hobza, 2012]. Our 7 Å cutoff fully encompasses any hydrogen bonds directly relevant to ligand binding.

Water-mediated interactions: FLOWR.ROOT treats water implicitly, consistent with many previous ML approaches. Our case study on Hsp90 (Appendix B.3) demonstrates that explicit water inclusion improves correlation with QM binding energies, suggesting potential for future enhancement given that future model and compute infrastructure improvements make this possible.

Entropic effects: Vibrational entropy terms, the major contributors to binding entropy, arise from direct protein-ligand interactions and are captured within the 7 Å radius. Rotational and translational terms are dominated by protein contributions and are relatively constant within ligand families, allowing implicit learning. Conformational entropy changes in the binding pocket are directly included, while distal conformational changes cancel between bound and unbound states.

We note that the cutoff is a configurable parameter that can be adjusted at both training and inference time. The current choice balances accuracy with computational efficiency. We have added a discussion of cutoff considerations to the revised manuscript (Supplementary Information—Methods).

Comment 1.6 – Data Leakage Concerns

CrossDocked2020 and BindingNet involve shared protein backbones and docking templates. Without explicit guarantees that protein families, templates, or backbone conformations do not overlap between training and test sets, reported improvements in pose generation and affinity prediction may be artificially inflated.

Response:

We take data leakage seriously and have implemented rigorous splitting strategies throughout this work:

1. **Plinder-based splitting:** All protein-ligand datasets are consistently split according to the Plinder dataset splits [Durairaj et al., 2024], a robust split utilizing comprehensive similarity metrics (protein sequence, pocket-level Jaccard, interaction-level PLIP, and ligand-level Tanimoto similarity) to ensure minimal leakage and high-quality, non-redundant testing. To our knowledge, this represents the most rigorous publicly available splitting strategy for structure-based modeling.
2. **Base model evaluation:** The evaluations on GEOM-Drugs (Supplementary Information, Table 3), CrossDocked2020 (Supplementary Information, Table 4), and SPINDR (Section 2.3, Table 1) are performed on FLOWR.ROOT^{base}—a model trained **from scratch** on these specific datasets without any pre-training. This ensures direct comparability with prior work and eliminates the possibility of information leakage from pre-training data. The substantial performance improvements stem from architectural enhancements and improved loss functions.
3. **Project-specific evaluation:** Section 2.6 demonstrates evaluation on diverse proprietary project datasets with targets and compounds that were never seen during any training phase and thus unseen structure–activity landscapes.

The comparison with Boltz-2 on project-specific data is particularly informative: both models were trained on public domain data and fail to predict potency for unseen structure–activity landscapes. However, FLOWR.ROOT with LoRA finetuning substantially increases downstream performance with minimal compute resources underlining the effectiveness of our proposed framework. To better highlight this, we substantially increased Section 2.6, and added Section 2.7.

Comment 1.7 – Equivariance Under Masking

The authors use residue embeddings and local environmental vectors extracted from a truncated radius sphere. However, when pocket atoms at different distances are masked or omitted, the equivariance assumptions of the model no longer strictly hold. The paper does not analyze the conditions under which this approximation is valid, nor whether equivariance is maintained for irregular pocket geometries.

Response:

We believe there may be a misunderstanding of our architecture. FLOWR.ROOT does not use ”local environmental vectors”. The pocket encoder processes features through $SE(3)$-equivariant self-attention layers using:

- Invariant residue embeddings
- Equivariant, full-atom coordinate features
- Pairwise full-atom, **fully-connected** distance information

$SE(3)$-equivariance is strictly maintained. While data curation, the pocket extraction is a preprocessing step that defines the "system boundary". There is no approximation introduced by the cutoff itself; rather, the cutoff determines which atoms are included in the equivariant computations. Via fully-connected, full-atom equivariant cross-attention, the ligand decoder interacts with the invariant and equivariant pocket encodings for ligand generation. We added clarifying statements to Supplementary Information—Methods.

Comment 1.8 – Novelty Concerns

The core architectural ideas: equivariant flows, pocket conditioning, and molecular generation, are very close to those in FLOWR, FLOWR.MULTI, and PILOT. The primary differences seem to involve larger datasets and joint affinity training. They do not constitute a sufficiently novel conceptual contribution for Nature Communications.

Response:

We thank the reviewer for this question. FLOWR.ROOT is distinct from existing approaches in several ways:

1. **Practical framework:** FLOWR.ROOT introduces a comprehensive framework for ligand- and structure-based drug discovery, covering applications from hit identification to lead optimization. The combination of multi-mode generation, joint affinity prediction, importance sampling–based steering, and efficient domain adaptation via LoRA fine-tuning yields a coherent and practical tool for real-world drug discovery.
2. **Joint training objective:** To our knowledge, FLOWR.ROOT is the first model to jointly train structure-aware 3D ligand generation with multi-endpoint affinity prediction (pIC_{50}, pK_i, pK_d, pEC_{50}) and confidence estimation within a unified framework. This integration enables accurate, in-distribution property-guided generation without external scoring functions, supports effective domain adaptation via parameter-efficient fine-tuning, multi-target selectivity and affinity-guided fragment-elaboration.
3. **Multi-fidelity training paradigm:** We propose a comprehensive three-stage training framework that systematically leverages public-domain datasets of varying quality, directly addressing the scarcity of high-quality, affinity-annotated structural data through principled data integration.
4. **Substantially improved performance:** FLOWR.ROOT delivers consistent and substantial gains across all benchmarks, spanning ligand-only (GEOM-Drugs) and structure-based (CrossDocked2020, SPINDR) molecule generation, as well as potency and affinity prediction (HiQBind, FEP+/OpenFE, four in-house targets, PDE10A). In particular, we observe significantly reduced strain energies on SPINDR (50.36 vs. 90.05 kcal/mol) and improved PoseBusters validity (0.97 vs. 0.93). We further demonstrate state-of-the-art affinity prediction relative to computationally expensive physics-based methods (FEP+, OpenFE) and SOTA ML-based methods (Boltz-2, AEV-PLIG) across diverse target families. FLOWR.ROOT-generated ligands support direct quantum-mechanical evaluation without any post-processing across multiple targets (CK2α, TYK2, ERα, BACE1). With FLOWR.ROOT, we introduce enhanced latent message-passing layers with pairwise distance features and cross-product computations, as well as residual coordinate updates, include radial basis function expansions between ligand-ligand, pocket-pocket and ligand-pocket atoms for enhanced representation learning, and propose improved loss functions incorporating bond angles and bond lengths losses (Supplementary Information—Methods).
5. **Novel generation modes:** FLOWR.ROOT introduces a new set of fragment-conditional generation modes together with an inference framework that supports not only scaffold hopping, scaffold elaboration, or fragment linking, but also local fragment growing and fragment replacement. For the latter two tasks, we propose a flexible prior-placement strategy that shifts the random prior from a zero center-of-mass to a user-defined local replacement site, thereby enforcing local growth or

replacement. This enables spatially predefined fragment growing and locally confined fragment replacement via custom atom selection, which is important for fragment-based virtual screening as well as for hit-to-lead and lead optimization, respectively.

6. **Parameter-efficient domain adaptation:** Importantly, we demonstrate parameter-efficient fine-tuning for structure-based drug discovery via LoRA fine-tuning on multiple in-house datasets and on a public, high-quality dataset (PDE10A). This enables practical deployment in settings where even a large-scale, state-of-the-art model such as Boltz-2 fails to generalize.

We have clarified these contributions in the revised Introduction and made adjustments to the Supplementary Information—Methods.

Minor Comments

Minor Comment

Several figures lack statistical uncertainty or variance estimates.

Response:

We note that uncertainty quantification is provided throughout the manuscript:

- All table values include either 95% confidence intervals or standard deviations as subscripts.
- Scatter plots include error bars (Figures 2, 4, 5) representing standard deviations across seed runs. Note, for affinity prediction, standard deviations are small across seeds and thus hard to see, so we extended all reported mean values in the main text with 95% confidence intervals.
- Scatter plots include density coloring to indicate prediction frequency, with metrics computed across multiple seed runs.
- Bar plots show 95% confidence intervals as error bars.

We have reviewed all figures to ensure consistent uncertainty reporting in the revised manuscript, and added standard deviations or 95% confidence intervals throughout to the main text.

Reviewer #2

General Assessment

The authors present a foundation model for ligand generation with binding affinity prediction and confidence estimation. The model addresses a long-standing challenge of drug discovery and related fields. Therefore the paper represents an important advance, and is potentially interesting.

Major Comments

Comment 2.1 – Distinction from Prior Work

The authors need to demonstrate and articulate more clearly how the new foundation model is distinct and/or superior to earlier approaches.
Specifically: There are a number of other foundation models (some of which are included in the *Results* section of the manuscript) which aim to do similar things – ligand generation, binding affinity prediction etc. They typically have robust zero-shot protein–ligand docking and few-shot binding affinity capabilities, but still require finetuning to be fully applicable in a drug discovery setting.
The foundation model presented in this manuscript appears to be relatively similar, albeit with superior performance across different dimensions. The authors should articulate more clearly if their model is (i) similar-but-slightly-better; or (ii) distinct in some fundamentally different way.
If it's (i), then the authors need to provide a deeper discussion of what enabled the superior performance (e.g. training data used, data and evidence of how the model learns, evidence of scaling/speed etc.)
And if it's (ii), then we need a clearer articulation of what the fundamental differences are.

Response:

We thank the reviewer for this question. FLOWR.ROOT is distinct from existing approaches in several ways:

1. **Practical framework:** FLOWR.ROOT introduces a comprehensive framework for ligand- and structure-based drug discovery, covering applications from hit identification to lead optimization. The combination of multi-mode generation, joint affinity prediction, importance sampling–based steering, and efficient domain adaptation via LoRA fine-tuning yields a coherent and practical tool for real-world drug discovery.
2. **Joint training objective:** To our knowledge, FLOWR.ROOT is the first model to jointly train structure-aware 3D ligand generation with multi-endpoint affinity prediction (pIC_{50}, pK_i, pK_d, pEC_{50}) and confidence estimation within a unified framework. This integration enables accurate, in-distribution property-guided generation without external scoring functions, supports effective domain adaptation via parameter-efficient fine-tuning, multi-target selectivity and affinity-guided fragment-elaboration.
3. **Multi-fidelity training paradigm:** We propose a comprehensive three-stage training framework that systematically leverages public-domain datasets of varying quality, directly addressing the scarcity of high-quality, affinity-annotated structural

data through principled data integration.

4. **Substantially improved performance:** FLOWR.ROOT delivers consistent and substantial gains across all benchmarks, spanning ligand-only (GEOM-Drugs) and structure-based (CrossDocked2020, SPINDR) molecule generation, as well as potency and affinity prediction (HiQBind, FEP+/OpenFE, four in-house targets, PDE10A). In particular, we observe significantly reduced strain energies on SPINDR (50.36 vs. 90.05 kcal/mol) and improved PoseBusters validity (0.97 vs. 0.93). We further demonstrate state-of-the-art affinity prediction relative to computationally expensive physics-based methods (FEP+, OpenFE) and SOTA ML-based methods (Boltz-2, AEV-PLIG) across diverse target families. FLOWR.ROOT-generated ligands support direct quantum-mechanical evaluation without any post-processing across multiple targets (CK2α, TYK2, ERα, BACE1). With FLOWR.ROOT, we introduce enhanced latent message-passing layers with pairwise distance features and cross-product computations, as well as residual coordinate updates, include radial basis function expansions between ligand-ligand, pocket-pocket and ligand-pocket atoms for enhanced representation learning, and propose improved loss functions incorporating bond angles and bond lengths losses (Supplementary Information—Methods).
5. **Novel generation modes:** FLOWR.ROOT introduces a new set of fragment-conditional generation modes together with an inference framework that supports not only scaffold hopping, scaffold elaboration, or fragment linking, but also local fragment growing and fragment replacement. For the latter two tasks, we propose a flexible prior-placement strategy that shifts the random prior from a zero center-of-mass to a user-defined local replacement site, thereby enforcing local growth or replacement. This enables spatially predefined fragment growing and locally confined fragment replacement via custom atom selection, which is important for fragment-based virtual screening as well as for hit-to-lead and lead optimization, respectively.
6. **Parameter-efficient domain adaptation:** Importantly, we demonstrate parameter-efficient fine-tuning for structure-based drug discovery via LoRA fine-tuning on multiple in-house datasets and on a public, high-quality dataset (PDE10A). This enables practical deployment in settings where even a large-scale, state-of-the-art model such as Boltz-2 fails to generalize.

We have clarified these contributions in the revised Introduction and made adjustments to the Supplementary Information—Methods.

Comment 2.2 – External Validation

Beyond the data shown in the *Results* section (which are self-reported) is there any way to demonstrate performance in an external and independent setting?
For example by participating in external competitions, such as the CACHE challenge. If this is not possible in a reasonable timeframe, the authors should commit to participating in future such competitions.

Response:

We appreciate this suggestion and acknowledge the importance of independent validation. Several points address this concern:

1. **Ongoing external validation:** We are collaborating with the OpenFE consortium

to conduct independent validation. As this in ongoing work, results are not available for inclusion in this manuscript.

2. **Expanded in-house validation:** To strengthen the evidence for generalization, we have expanded Section 2.6 to include evaluation on **four diverse in-house projects** spanning oncology, inflammatory and neurodegenerative disease. These projects involve different target classes, chemical series, and SAR landscapes. In all cases, we evaluated both FLOWR.ROOT and Boltz-2, demonstrating that LoRA-finetuned FLOWR.ROOT consistently achieves strong performance while also Boltz-2 fails to generalize, underlining the effectiveness of the proposed framework. We also added evaluation of LoRA-finetuning on the public domain PDE10A dataset in Section 2.7.
3. **Benchmark reproducibility:** All public benchmark evaluations (FEP+, OpenFE, SPINDR, CrossDocked2020, GEOM-Drugs) use established datasets and protocols, enabling independent reproduction.
4. **Ease-of-use** We not only provide code and model checkpoints for reproducing our work, but also a comprehensive README and an extensive tutorial notebook on the publicly available GitHub repository. With that, FLOWR.ROOT can be used and adopted by the community in its full capacity allowing for independent validation in different design scenarios.

As FLOWR.ROOT is built for and meant to be used in hit-to-lead and lead optimization stages of early drug discovery campaigns—the model requires the 3D structure of a protein and its binding site defined by a hit or lead compound. Normally, this setting is not covered in challenges like CACHE. However, as mentioned above, we are working on an external validation together with OpenFE combining physics-based with ML modeling. In addition, we are working on the release of a dense, out-of-distribution SAR dataset to better and more rigorously evaluate future open source models.

Comment 2.3 – Manuscript Structure

In many places, the manuscript reads a bit like a PhD thesis, rather than an academic or industrial publication. To make it suitable as a publication, I would recommend restructuring the manuscript extensively.
Specifically:

- Section 1 (*Introduction*) and Section 2 (*Related works*) read a bit like a review. Both should be substantially condensed.
- Section 3 (*Datasets*) has an extensive list and description of datasets. This should be shortened and/or moved to a supplementary file.
- Section 4 (*Methods*) should be moved to a supplementary file; the main manuscript could include a condensed version of the methods.
- Section 5 (*Results*) is of course the most important, but at the moment, it's 12 pages long which feels unwieldy. Could this be shortened and sharpened? Less-critical results could be moved to supplemental file.

Response:

We thank the reviewer for this constructive feedback and have restructured the manuscript accordingly:

- **Datasets:** Moved to Supplementary Information—Datasets. The main text now

contains a brief description of the data sources and curation strategy in the Overview section.

- **Related Works:** Moved to Supplementary Information—Related Works.
- **Results:** The GEOM-Drugs and CrossDocked2020 benchmark results have been moved to Supplementary Information—Additional Results. The main Results section now focuses on: (1) SPINDR evaluation, (2) affinity prediction benchmarks, (3) domain adaptation, and (4) case studies. Brief summaries of the moved results are retained in the main text.
- **Main text:** After the restructuring, the total word count, excluding table and figure captions, is approximately 5500, thus complying with the 6000-word limit set by Nature Communications.

Comment 2.4 – Conflicts of Interest

I note that some of the authors are affiliated with a pharmaceutical company; other authors are based in academic institutions. Given the subject matter of the article (drug discovery), the manuscript therefore needs to include a declaration of potential conflicts of interest. All funding sources, industrial as well as academic, need to be fully acknowledged.

Response:

We have added a Competing Interests to the revised manuscript as well as a Funding statement. However, we note that we don't have any competing interests to declare, nor any funding. We open sourced the entire FLOWR.ROOT code and framework as well as all the data used in this study.

Comment 2.5 – Data Leakage

In the *Datasets* section and in the *Results* section, the authors talk about many similar datasets. The authors need to describe more clearly how they avoided data leakage – otherwise the superior performance reported in the manuscript may not be believed.

Response:

We thank the reviewer for this important comment. We have addressed data leakage concerns through multiple safeguards:

1. **Plinder-based splitting:** All protein-ligand datasets are consistently split according to the Plinder dataset splits [Durairaj et al., 2024], a robust split utilizing comprehensive similarity metrics (protein sequence, pocket-level Jaccard, interaction-level PLIP, and ligand-level Tanimoto similarity) to ensure minimal leakage and high-quality, non-redundant testing. To our knowledge, this represents the most rigorous publicly available splitting strategy for structure-based modeling.
2. **Base model evaluation:** The evaluations on GEOM-Drugs (Supplementary Information, Table 3), CrossDocked2020 (Supplementary Information, Table 4), and SPINDR (Section 2.3, Table 1) are performed on FLOWR.ROOT^{base}—a model trained **from scratch** on these specific datasets without any pre-training. This ensures direct comparability with prior work and eliminates the possibility of information

leakage from pre-training data. The substantial performance improvements stem from architectural enhancements and improved loss functions.

3. **Project-specific evaluation:** Section 2.6 demonstrates evaluation on diverse proprietary project datasets with targets and compounds that were never seen during any training phase and thus unseen structure–activity landscapes.

Reviewer #3

General Assessment

The authors present an impressively large-scale training pipeline, integrating diverse datasets such as BindingNet, SAIR, and SPINDR. This data-driven strategy aligns well with current trends in deep learning for drug discovery. Furthermore, the application of importance sampling for property steering demonstrates the model's potential flexibility in practical drug design scenarios. However, there are several concerns regarding the validation of the joint training objective, the generalization capabilities of the model, and the rigor of the evaluation metrics.

Major Comments

Comment 3.1 – Ablation Studies on Affinity Head

While the joint training of structure generation and affinity prediction is a core contribution, the manuscript lacks a controlled ablation study to quantify the specific contribution of the affinity head to the quality of structure generation. It is unclear whether the performance gains are derived from the auxiliary affinity supervision or simply from the massive scale of the training data. A comparison between a model trained solely on structure generation versus the joint model (controlled for dataset size) is necessary to validate the architectural benefit.

Response:

We wish to clarify that **we do not claim** that the affinity head improves pose quality or structural sampling. The structural generation and affinity prediction components serve distinct, complementary purposes:

- The **structure head** generates geometrically valid ligand conformations within the protein pocket enabling high structural and pose quality
- The **affinity head** provides potency and affinity estimates enabling: (1) in-distribution evaluation of generated structures, (2) inference-time steering toward higher-affinity compounds via importance sampling.
- Effective joint structure-affinity domain adaptation via LoRA-finetuning to unseen structure–activity landscapes.
- Multi-target selectivity via joint structure and affinity prediction
- Fragment-based elaboration combining conditional structure with affinity prediction.

One of the primary benefits of joint training is enabling coherent, in-distribution property-guided generation during inference, as demonstrated in Section 2.4 (Figure 3). Without the jointly trained affinity head, importance sampling-based steering would require external scoring functions, introducing not only substantial additional computational overhead, but also distribution mismatch potentially enforcing higher failure modes on the structure predictions (Section 2.4). Importantly, joint learning also enables to effectively finetune the model on unseen structure–activity landscapes, e.g. via LoRA (Section 2.6 and 2.7). In addition, a joint structure and affinity prediction model can be used to run multi-target selectivity campaigns, as well as fragment-conditional ligand generation with affinity prediction and guidance for effective local chemical space exploration (Section 2.8 and 2.9).

Comment 3.2 – Generalization vs. Memorization

There is a concern regarding whether the model is genuinely "predicting" interactions or merely "memorizing" the training distribution. Section 5.6 acknowledges that the model exhibits negligible correlation on in-house data without fine-tuning, which hints at poor zero-shot generalization.
To demonstrate true utility, the authors should evaluate:

2.1 The novelty of generated molecules (e.g., similarity distributions relative to the training set).

2.2 The model's ability to generate valid, high-quality binders for out-of-distribution (OOD) targets without reliance on project-specific fine-tuning.

Response:

We thank the reviewer for raising this important aspect.
Novelty of generated molecules: We added a comprehensive overview of the performance of FLOWR.ROOT on the SPINDR test dataset to the Supplementary Information—Additional Results (Tables 5–8) covering a wide range of metrics including novelty, uniqueness and diversity in comparison to the FLOWR model, including results on a set of fragment-conditional design tasks. All results indicate substantial improvements of FLOWR.ROOT without exhibiting any form of mode collaps—novelty: 1.00 vs. 0.94, uniqueness: 0.89 vs. 0.94, diversity: 0.83 vs. 0.86—indicating that the model is able to generate novel chemical matter.
OOD generalization: We argue that expecting universal zero-shot generalization across arbitrary bioactivity landscapes is unrealistic given the complexity of structure—activity relationships, as shown in Section 2.6 evaluating both Boltz-2 and FLOWR.ROOT extensively on a diverse set of in-house targets. Based on our finding, our position is that models should function as "dynamic companions" continually requiring adaptation to project-specific SAR patterns.
However, several observations support genuine learning and generalization:

- The QM validation studies (Section 2.8) demonstrate that FLOWR.ROOT-can not only generate novel ligands with high pose accuracy allowing for direct QM validation, but also show that generated ligands exploit physically meaningful, non-trivial interaction patterns.
- The selectivity study (CK2α/CLK3) shows the model discovers selectivity mechanisms consistent with prior biochemical knowledge (Section 2.8).
- Importantly, in contrast to the zero-shot affinity prediction capabilities of FLOWR.ROOT, zero-shot structure prediction yields physically realistic structures also on out-of-distribution systems. We added an analysis to the Supplementary Information—Additional Results (Section C.3 and Fig. 15; Section C.4 and Fig. 16).
- With LoRA finetuning on limited project data, the model achieves effective domain adaption showing strong performance, suggesting it has learned transferable internal representations (Section 2.6, 2.7; Supplementary Information—Additional Results: Section C.3 and Fig. 15; Section C.4 and Fig. 16).

Comment 3.3 – Diversity Under Affinity Steering

In Section 5.4, the authors discuss affinity steering via importance sampling. However, an analysis of internal diversity is missing. As the guidance strength increases, there is a significant risk of mode collapse, where the model may converge on a limited number of high-scoring but repetitive structures. The authors should provide metrics (e.g., IntDiv or Uniqueness) plotted against steering intensity to demonstrate that the model maintains chemical diversity while optimizing for affinity.

Response:

We thank the reviewer for this constructive feedback. We have added a structural and diversity analysis for steered samples to Section 2.4:
As steering duration increases from unguided to guided:

- Mean predicted pIC_{50} shifts from 5.60 to 6.02
- Diversity (IntDiv): 0.79 → 0.79
- PoseBusters validity: 0.93 → 0.91
- Strain energy: 48.46 → 54.42
- Vina score: -7.41 → -7.50

Thus, we observe only a modest decrease in mean PoseBusters-validity and increase in mean strain energy, while diversity rates stay constant and Vina scores decrease. This shows that guided samples maintain physically realistic structures while optimizing for affinity.

Comment 3.4 – Insufficient Benchmarking in Section 5.4

In the affinity prediction evaluation on SPINDR (Section 5.4), the comparison is limited to the pre-trained FLOWR.ROOT versus the non-pre-trained base model. This only proves the value of pre-training. To demonstrate the superiority of the Flow Matching architecture itself, the authors should compare their approach against other state-of-the-art affinity prediction architectures (e.g., GNNs or Transformers) on this specific benchmark.

Response:

We thank the reviewer for this comment. Comprehensive model comparisons are provided on the FEP+/OpenFE benchmarks (Sections 2.5), where FLOWR.ROOT is compared against:

- **AEV-PLIG**: A state-of-the-art GNN-based affinity predictor with data augmentation
- **Boltz-2**: A large-scale co-folding model with most recent state-of-the-art affinity prediction performance

These demonstrate state-of-the-art performance of FLOWR.ROOT's architecture relative to recent ML-based models and competitive performance relative to expensive, physics-based approaches across a wide range of diverse targets.
For HIQBIND specifically, we note that this dataset was just recently established and benchmarks do not yet exist for other architectures. We hope the community to adopt our results for future benchmarking.
In addition, we also added a comprehensive evaluation on a diverse set of in-house targets and a public domain high-quality benchmark dataset, PDE10A, comparing FLOWR.ROOT

with Boltz-2 (Section 2.6 and 2.7).

Comment 3.5 – Incomplete Validation of "Valid" Generation

The conclusion that the model can be biased toward higher affinity "without sacrificing chemical or geometric validity" is not fully supported by the data.

5.1 Figure 4 shows that the SA-score (Synthetic Accessibility) actually degrades (shifts higher) with guidance.

5.2 Crucially, geometric validity metrics—such as Strain Energy and Vina Scores—are missing for the steered samples. It is vital to prove that forcing high-affinity scores does not result in physically unrealistic or highly strained conformations.

Response:

We thank the reviewer for this important observation. We have addressed both points:
SA-score: The reviewer correctly notes that the SA-score distribution shifts slightly with steering. However, we also find that the QED of guided samples shows a clear increase and we note that consequently the Lipinski score increases from 4.30 to 4.68 indicating an improvement regarding druglikeness. We have added this analysis to the revised Section 2.4.
Geometric validity under steering: We have added PoseBusters-validity, strain energy and Vina score analysis for steered samples:

- PoseBusters validity: 0.93 → 0.91
- Strain energy: 48.46 → 54.42
- Vina score: -7.41 → -7.50

The data show modest increases in strain energy and slight improvements in Vina scores, with high geometric validity maintained. We have added this analysis to the revised Section 2.4.

Minor Comments

Comment 3.6 – Typo in Section 4.2

The text states, "Both S and P are stacked to the existing pairwise message tensor..." However, based on the preceding equations, the cross-product tensor is defined as C, not P. Please correct this variable name.

Response:

We thank the reviewer for catching this error. We corrected the text (Supplementary Information—Methods).

Comment 3.7 – Interpretation of Figure Density

The statement, “The denser regions... represent higher prediction frequencies, indicating where the model performs best,” is logically flawed. High density in a scatter plot typically correlates with the bulk of the training data distribution (data bias) rather than the intrinsic accuracy of the model. “Best performance” should be defined by prediction error (RMSE/MAE) across the dynamic range, not by the frequency of predictions in the mean range. Please rephrase this interpretation.

Response:

We thank the reviewer for catching this error. We have corrected the figure caption.

Comment 3.8 – Misleading Metrics in Figure 9

The reviewer notes significant confusion regarding the metrics box presented in Figure 9 (Top Left), which reports an RMSE of 25.06 kcal/mol. This plot compares the QM binding energies of CLK3 (off-target) versus CK2α (on-target) to demonstrate selectivity. In this context, a large deviation from the identity line (high RMSE) is the intended outcome (selectivity), not an error. Labeling this as “RMSE” suggests poor performance and is highly misleading. The authors should remove these regression metrics or strictly contextualize them as a selectivity measurement.

Response:

We thank the reviewer for this observation. We removed RAE and RMSE from the figure and added a description to the figure caption and main text to avoid any confusion.

References

Christoph Bannwarth, Sebastian Ehlert, and Stefan Grimme. Gfn2-xtb—an accurate and broadly parametrized self-consistent tight-binding quantum chemical method with multipole electrostatics and density-dependent dispersion contributions. *Journal of Chemical Theory and Computation*, 15(3):1652–1671, Mar 2019. ISSN 1549-9618. doi: 10.1021/acs.jctc.8b01176. URL https://doi.org/10.1021/acs.jctc.8b01176.

Qiang Cui and Marcus Elstner. Density functional tight binding: values of semi-empirical methods in an ab initio era. *Phys. Chem. Chem. Phys.*, 16:14368–14377, 2014. doi: 10.1039/C4CP00908H. URL http://dx.doi.org/10.1039/C4CP00908H.

Janani Durairaj, Yusuf Adeshina, Zhonglin Cao, Xuejin Zhang, Vladas Oleinikovas, Thomas Duignan, Zachary McClure, Xavier Robin, Danny Kovtun, Emanuele Rossi, Guoqing Zhou, Srimukh Veccham, Clemens Isert, Yuxing Peng, Prabindh Sundareson, Mehmet Akdel, Gabriele Corso, Hannes Stärk, Zachary Carpenter, Michael Bronstein, Emine Kucukbenli, Torsten Schwede, and Luca Naef. Plinder: The protein-ligand interactions dataset and evaluation resource. *bioRxiv*, 2024. doi: 10.1101/2024.07.17.603955. URL https://www.biorxiv.org/content/early/2024/07/17/2024.07.17.603955.

M. Elstner. The scc-dftb method and its application to biological systems. *Theoretical Chemistry Accounts*, 116(1):316–325, 2006. doi: 10.1007/s00214-005-0066-0.

Michael Gaus, Qiang Cui, and Marcus Elstner. Density functional tight binding: application to organic and biological molecules. *WIREs Computational Molecular Science*, 4(1):49–61, 2014a. doi: https://doi.org/10.1002/wcms.1156. URL https://wires.onlinelibrary.wiley.com/doi/abs/10.1002/wcms.1156.

Michael Gaus, Xiya Lu, Marcus Elstner, and Qiang Cui. Parameterization of dftb3/3ob for sulfur and phosphorus for chemical and biological applications. *Journal of Chemical Theory and Computation*, 10(4):1518–1537, 2014b. doi: 10.1021/ct401002w. URL https://doi.org/10.1021/ct401002w. PMID: 24803865.

Przemyslaw Grygier, Katarzyna Pustelny, Jakub Nowak, Przemyslaw Golik, Grzegorz M. Popowicz, Oliver Plettenburg, Grzegorz Dubin, Filipe Menezes, and Anna Czarna. Silmitasertib (cx-4945), a clinically used ck2-kinase inhibitor with additional effects on gsk3b and dyrk1a kinases: A structural perspective. *Journal of Medicinal Chemistry*, 66(6):4009–4024, 2023. doi: 10.1021/acs.jmedchem.2c01887. URL https://doi.org/10.1021/acs.jmedchem.2c01887. PMID: 36883902.

Przemyslaw Grygier, Katarzyna Pustelny, Filipe Menezes, Malgorzata Jemiola-Rzeminska, Piotr Suder, Grzegorz Dubin, and Anna Czarna. Structural perspective on the design of selective dyrk1b inhibitors. *International Journal of Biological Macromolecules*, 330: 148124, 2025. ISSN 0141-8130. doi: https://doi.org/10.1016/j.ijbiomac.2025.148124. URL https://www.sciencedirect.com/science/article/pii/S0141813025086817.

Izabella Góral, Tomasz Wichur, Emilia Sługocka, Przemysław Grygier, Monika Głuch-Lutwin, Barbara Mordyl, Ewelina Honkisz-Orzechowska, Natalia Szałaj, Justyna Godyń, Dawid Panek, Paula Zaręba, Anna Sarka, Paweł Żmudzki, Gniewomir Latacz, Katarzyna Pustelny, Adam Bucki, Anna Czarna, Filipe Menezes, and Anna Więckowska. Exploring novel gsk-3 inhibitors for anti-neuroinflammatory and neuroprotective effects: Synthesis, crystallography, computational analysis, and biological evaluation. *ACS Chemical Neuroscience*, 15(17):3181–3201, 2024. doi: 10.1021/acschemneuro.4c00365. URL https://doi.org/10.1021/acschemneuro.4c00365. PMID: 39158934.

Mehran Jalaie, Jindřich Fanfrlík, Adam Pecina, Martin Lepšík, and Jan Řezáč. Comparative analysis of quantum-mechanical and standard single-structure protein–ligand scoring functions with md-based free energy calculations. *Journal of Chemical Information and Modeling*, 65(15):8127–8136, 2025. doi: 10.1021/acs.jcim.5c00604. URL https://doi.org/10.1021/acs.jcim.5c00604. PMID: 40682535.

Maximilian Kubillus, Tomáš Kubař, Michael Gaus, Jan Řezáč, and Marcus Elstner. Parameterization of the dftb3 method for br, ca, cl, f, i, k, and na in organic and biological systems. *Journal of Chemical Theory and Computation*, 11(1):332–342, 2015. doi: 10.1021/ct5009137. URL https://doi.org/10.1021/ct5009137.

Michał Nowacki, Filipe Menezes, Emilia Pykacz, Mateusz Popiołek, Valeria Napolitano, Chethan K. Krishna, Vishal C. Kalel, Ralf Erdmann, Tony Fröhlich, Oliver Plettenburg, Michael Sattler, Grzegorz M. Popowicz, and Maciej Dawidowski. Quantum mechanics-driven structure-activity relationship study of pex5-pex14 protein-protein interaction inhibitors based on a dibenzo[b,e]azepin-6(6h)-one scaffold. *European Journal of Medicinal Chemistry*, 298: 117979, 2025. ISSN 0223-5234. doi: https://doi.org/10.1016/j.ejmech.2025.117979. URL https://www.sciencedirect.com/science/article/pii/S0223523425007445.

Saro Passaro, Gabriele Corso, Jeremy Wohlwend, Mateo Reveiz, Stephan Thaler, Vignesh Ram Somnath, Noah Getz, Tally Portnoi, Julien Roy, Hannes Stark, David Kwabi-Addo, Dominique Beaini, Tommi Jaakkola, and Regina Barzilay. Boltz-2: Towards accurate and efficient binding affinity prediction. *bioRxiv*, 2025. doi: 10.1101/2025.06.14.659707. URL https://www.biorxiv.org/content/early/2025/06/18/2025.06.14.659707.

Adam Pecina, Jindřich Fanfrlík, Martin Lepšík, and Jan Řezáč. Sqm2.20: Semiempirical quantum-mechanical scoring function yields dft-quality protein–ligand binding affinity predictions in minutes. *Nature Communications*, 15:1127, 2024.

James J. P. Stewart. Optimization of parameters for semiempirical methods v: Modification of nddo approximations and application to 70 elements. *Journal of Molecular Modeling*, 13(12): 1173–1213, 2007. doi: 10.1007/s00894-007-0233-4.

Ísak Valsson, Matthew T. Warren, Charlotte M. Deane, Aniket Magarkar, Garrett M. Morris, and Philip C. Biggin. Narrowing the gap between machine learning scoring functions and free energy perturbation using augmented data. *Communications Chemistry*, 8(1):41, Feb 2025. ISSN 2399-3669. doi: 10.1038/s42004-025-01428-y. URL https://doi.org/10.1038/s42004-025-01428-y.

Yingfeng Zhang, Jin Xiao, Shunyu Wang, Tong Zhu, and John Z. H. Zhang. The atomic density-based tight-binding (atb) model: A robust and accurate semiempirical method parametrized for h–ra; applied to structures, vibrational frequencies, noncovalent interactions, and excited states. *Journal of Chemical Theory and Computation*, 21(7):3410–3425, 2025. doi: 10.1021/acs.jctc.4c01694. URL https://doi.org/10.1021/acs.jctc.4c01694. PMID: 40152646.

Jan Řezáč and Pavel Hobza. Advanced corrections of hydrogen bonding and dispersion for semiempirical quantum mechanical methods. *Journal of Chemical Theory and Computation*, 8(1):141–151, 2012. doi: 10.1021/ct200751e. URL https://doi.org/10.1021/ct200751e. PMID: 26592877.

Rebuttal Letter — Second Revision

FLOWR.root: A flow matching based foundation model for joint multi-purpose structure-aware 3D ligand generation and affinity prediction

Manuscript ID: NCOMMS-25-95570-T

Nature Communications

Dear Reviewers,

Thank you for the continued constructive engagement with our manuscript. We have carefully addressed the remaining comments raised by Reviewer #1, as well as the suggestions from Reviewer #2. Below, we provide point-by-point responses to all comments. Text additions to the revised manuscript are indicated in blue.

In addition, we now introduce FLOWR.UI, a browser-based graphical user interface for interactive, structure-aware ligand design with FLOWR.ROOT. Beyond facilitating the reproduction of results, FLOWR.UI provides a code-free environment designed to lower the barrier to adoption across disciplines. The tool is described in the Supplementary Information, and its source code, together with usage instructions, is provided alongside the manuscript.

Sincerely,
The Authors

Reviewer #1

General Assessment

The manuscript has improved in clarity and organization following revision. However, several central methodological concerns remain unresolved. These issues directly affect the core claims of the work, and the revisions do not provide sufficient evidence to justify the main conclusions.

We thank Reviewer #1 for acknowledging the improvements in clarity and organization and for the continued engagement with our work. We address each remaining concern in detail below.

Major Comments

Comment 1.1 – Ablation Study on Affinity Head

The manuscript presents a joint structure-affinity architecture, yet no controlled ablation study has been provided to determine whether the affinity head meaningfully impacts structural generation. A structure-only model should be trained under identical conditions (same data, backbone, and hyperparameters) and directly compared to the joint model. Structural quality, diversity, stability across seeds, and convergence behavior should be

evaluated. Without this comparison, the architectural benefit of joint training remains unproved and not convincing.

Response:

We appreciate the reviewer's interest in rigorously understanding the architectural contributions of FLOWR.ROOT.

1. Clarification of claims: We wish to re-emphasize that the manuscript **does not claim** the affinity head improves structural generation quality, diversity, or convergence. The structure head and affinity head serve distinct, complementary functions: the structure head generates geometrically valid ligand conformations; the affinity head enables in-distribution property steering, domain adaptation via LoRA finetuning, multi-target selectivity, and affinity-guided fragment elaboration (Sections 2.4, 2.6–2.9). The requested ablation would therefore test a hypothesis we do not advance.

2. Existing evidence effectively constitutes an ablation. Multiple lines of evidence already isolate the sources of structural quality improvements:

- **FLOWR → FLOWR.ROOT^{base} comparison:** FLOWR.ROOT^{base} is trained **from scratch** on the same datasets, with the same training protocol, as the published FLOWR model—which has **no affinity head**. The substantial structural improvements (SPINDR: PB-validity 0.93 → 0.97, strain energy 90.05 → 50.36 kcal/mol; CrossDocked2020: PB-validity 0.92 → 0.97, strain energy 87.83 → 67.13 kcal/mol) are therefore attributable to explicit architectural enhancements: improved equivariant message-passing layers with pairwise distance and cross-product features, residual coordinate updates with Gaussian RBF expansions, and proposed bond-length and bond-angle losses (Supplementary Information—Methods). These changes are independent of the affinity head.
- **Unconditional generation (GEOM-Drugs):** The unconditional base model FLOWR.ROOT$^{\text{base}}_{\text{uncond.}}$ contains **no pocket encoder, no affinity head, and no pocket conditioning**—it is a purely structure-generating model. It achieves state-of-the-art PB-validity (0.94) and substantially lower relaxation RMSD (0.07 Å) compared to other methods, directly demonstrating that the architectural improvements to the backbone alone produce superior structural quality.

3. The affinity head is architecturally lightweight. The affinity head comprises four MLPs operating on pooled latent features. During training, the affinity loss is one of eight loss terms, with the remaining seven—coordinate MSE, atom-type CE, bond-type CE, charge CE, hybridization CE, bond-length MSE, and bond-angle Huber—collectively governing structural accuracy. The structure-related losses thus dominate the gradient signal to the shared backbone by a substantial margin. While gradients from the affinity loss do propagate through the shared representations, the relative contribution is by design substantially smaller—consistent with the well-established finding that multi-task learning from related domains provides implicit regularization rather than degradation [Ruder, 2017, Crawshaw, 2020].

4. Inference-time guidance provides a direct empirical test. In Section 2.4 (Fig. 3), we evaluate structural quality under affinity-guided importance sampling, where the affinity head is **actively used to reweight generative trajectories**. If multi-task training with the affinity head introduced detrimental biases into the shared representations, this would be maximally exposed during guided sampling. The results show minimal impact: PB-validity drops marginally from 0.93 (unguided) to 0.91 (guided at 0.5 strength), strain energy increases from 48.46 to 54.42 kcal/mol, and Vina scores slightly improve (−7.41

to −7.50 kcal/mol). Crucially, sample diversity remains unchanged (0.79 vs. 0.79). These results demonstrate that even under active affinity-driven steering, structural quality is preserved.

5. Computational considerations. A full controlled ablation—involving pre-training on ∼2.5M protein–ligand complexes, followed by finetuning, seed-replicated evaluation across all benchmarks, and statistical testing—would require several weeks of training on eight NVIDIA H100 GPUs per configuration, amounting to substantial computational and environmental cost. Given that (a) we do not claim a structural benefit from joint training nor can this be expected by design, (b) the FLOWR→FLOWR.ROOT^{base} comparison already isolates architectural contributions, (c) the unconditional model independently confirms structural improvements, and (d) the affinity head represents a small part of all model parameters with relatively small gradient contributions, we believe the existing evidence conclusively resolves this question without requiring such expenditure.

6. The purpose of joint training is functional integration. The affinity head's intended contribution is not to improve structural sampling—which would be an unexpected side effect—but to enable a unified workflow that supports: (1) in-distribution property-guided generation via importance sampling without external scoring functions (Section 2.4); (2) joint structure–affinity domain adaptation via LoRA finetuning on unseen SAR landscapes with as few as ∼200 datapoints (Sections 2.6, 2.7); (3) multi-target selectivity campaigns from a single model (Section 2.8); and (4) affinity-annotated fragment elaboration for local chemical space exploration (Section 2.9). Removing the affinity head would eliminate all of these capabilities—covering Sections 2.4 through 2.9 of the manuscript—without any demonstrated benefit to structural quality.

Comment 1.2 – Sensitivity to Computationally Generated Data

The model is trained on heterogeneous data, including computationally generated poses. The robustness of this strategy is not empirically demonstrated. A sensitivity analysis is needed. This could include retraining without lower-fidelity datasets or reporting performance stratified by data source. Without such analysis, it is unclear whether performance gains reflect architectural improvements or simply data scale and heterogeneity. For a foundation-model claim, robustness to data quality must be demonstrated.

Response:

We thank the reviewer for raising this important point. We address the reviewer's concerns along several axes: (1) the broader machine learning evidence for heterogeneous pretraining, (2) domain-specific evidence from computational chemistry, (3) our own empirical evidence, (4) the quality control measures integrated into our pipeline, and (5) computational considerations.

1. Broad machine learning evidence for heterogeneous pretraining. The principle that pretraining on large-scale, heterogeneous, and even lower-fidelity data improves downstream task performance is one of the most robustly established findings in modern machine learning. Yosinski et al. [2014] demonstrated that neural network features transfer across tasks even when source and target domains are quite different, with transferred features plus fine-tuning consistently outperforming training from scratch. Bommasani et al. [2021] formalized this as the foundation model paradigm, wherein models trained on broad, diverse data at scale are adaptable to a wide range of downstream tasks—with the

diversity of pretraining data identified as a critical enabler of transferability, not a liability. Mahajan et al. [2018] showed that large-scale pretraining on weakly labeled, noisy images achieved state-of-the-art transfer to ImageNet, directly demonstrating that massive heterogeneous data with imperfect annotations transfers remarkably well to clean downstream tasks. In physics, McCabe et al. [2023] showed that a single model pretrained on multiple heterogeneous physics simulations (compressible and incompressible Navier–Stokes, shallow water equations, diffusion–reaction systems) matches or outperforms task-specific baselines, and when finetuned on previously unseen physics with limited data, significantly outperforms training from scratch. This parallels our three-stage pipeline, where Stage 1 provides broad coverage across diverse protein–ligand systems and Stage 2 refines the model on high-fidelity binding data.

2. Domain-specific evidence from computational chemistry. Within drug discovery, the value of computationally generated data for improving binding affinity models has been independently demonstrated. Valsson et al. [2025] showed that augmenting training data with computationally generated BindingNet complexes improved benchmark performance substantially. Most directly relevant, Hsu et al. [2025] systematically investigated the impact of AI-predicted complex quality on binding affinity prediction using the same BindingNet dataset we employ. Their key findings are: (i) data augmentation benefits *critically* depend on structural quality—high-confidence BindingNet structures substantially improve model performance, while moderate- and low-confidence structures provide negligible returns; (ii) co-folded structures from Boltz-1x can effectively substitute for experimental structures when appropriately filtered.

3. Empirical evidence from FLOWR.ROOT. Our manuscript already provides a direct empirical test of the pretraining pipeline's value. As reported in Section 2.3, the FLOWR.ROOT^{base} model trained solely on HiQBind *without* large-scale pre-training shows substantially worse performance across all affinity metrics: RAE 0.70 ± 0.13 vs. 0.58 ± 0.06, RMSE 1.95 ± 0.24 vs. 1.18 ± 0.18, R^2 0.39 ± 0.13 vs. 0.57 ± 0.10, Kendall τ 0.49 ± 0.09 vs. 0.60 ± 0.06, and Pearson 0.63 ± 0.10 vs. 0.76 ± 0.07. This comparison—using identical architecture, identical test data, and the same Plinder-based splits minimizing information leakage—isolates the contribution of the multi-stage pretraining pipeline from architectural effects. The consistent and substantial improvements provide strong evidence that the heterogeneous pretraining pipeline contributes meaningfully to downstream performance.

4. Quality control measures are not ad hoc but principled. We want to emphasize that our use of heterogeneous data is not uncurated aggregation but follows a deliberate, quality-aware strategy informed by the literature cited above. Specifically:

- **SAIR:** From $\sim$ 5M raw complexes, we apply stringent multi-criteria filtering—retaining only PoseBusters-valid complexes with negative AutoDock-Vina scores, Boltz-1x confidence $\geq$ 0.8, iPTM $\geq$ 0.8, and interaction PTM $\geq$ 0.6—yielding $\sim$ 1.5M curated complexes.
- **BindingNet:** The 689,796 complexes are categorized by template matching quality into high-confidence (232,030), moderate-confidence (164,912), and low-confidence (292,813) subsets. Critically, the high-confidence subset is treated as higher-fidelity data on par with experimental co-crystal structures, while moderate- and low-confidence subsets are classified as lower-fidelity. This confidence-stratified treatment is directly supported by the findings of Hsu et al. [2025].
- **Lower- vs. higher-fidelity classification:** Complexes of computational origin (BindingNet moderate/low, SAIR, KIBA-3D, Kinodata-3D) are explicitly classified as lower-fidelity, while co-crystal datasets (Plinder, BindingMOAD) and the high-confidence BindingNet subset are classified as higher-fidelity. The progressive training paradigm

then ensures that Stage 2 finetuning on curated high-quality data (SPINDR, HiQBind) overrides any noise patterns from Stage 1.

5. Computational considerations. A systematic ablation study in which individual datasets with lower accuracy are removed from the Phase 1 pre-training would require repeated retraining of the entire pipeline, which not only incurs significant computational costs but is also not feasible in terms of time.

6. Summary. In summary, the robustness of our heterogeneous data strategy is supported by: (a) foundational transfer learning theory [Yosinski et al., 2014, Bommasani et al., 2021], (b) empirical evidence from computer vision [Mahajan et al., 2018] and physics [McCabe et al., 2023], (c) domain-specific validation in drug discovery [Valsson et al., 2025, Hsu et al., 2025], (d) our own pretrained-vs.-unpretrained comparison showing consistent and substantial gains, and (e) principled quality control measures (confidence-based filtering, fidelity stratification, progressive training) that are independently validated by the recent literature. We have added a discussion of our data heterogeneity strategy and its supporting evidence to the revised Supplementary Information—Data Heterogeneity and Quality Control.

Comment 1.3 – FEP+/OpenFE Benchmark Overlap

The authors acknowledge likely overlap between training data and the FEP+/OpenFE benchmark. In this case, superiority claims must be supported with quantitative overlap analysis (ligand and protein similarity), or the interpretation of these results must be tempered. If overlap exists, the results should not be framed as evidence of broad generalization.

Response:

We agree with the reviewer that the overlap between training data and the FEP+/OpenFE benchmark must be taken seriously. We want to first emphasize that the manuscript **already explicitly disclaims generalization** from these results. Section 2.5 states: "*we also want to emphasize that the Schrodinger FEP+/OpenFE dataset is likely significantly overlapping with the training data in terms of ligand and target space. While this applies to AEV-PLIG and Boltz-2 as well allowing for a somewhat fair comparison, we do not expect these results to indicate significant generalization capabilities.*" We therefore do not frame, and have not framed, these results as evidence of broad generalization. The overlap likely inflates absolute performance metrics for all evaluated models, including FLOWR.ROOT. Protein-level exposure means the models have learned binding site geometries and interaction patterns for these targets. This advantage cannot be disentangled from the reported numbers alone. We explicitly acknowledge this limitation and caution against interpreting the FEP+/OpenFE results as indicative of what performance one should expect on genuinely novel targets or chemical series.

However, the purpose of the FEP+/OpenFE evaluation is not to demonstrate generalization, but to enable a direct, controlled comparison against the only methods with rigorous industrial validation—FEP+ and OpenFE—on a shared, well-characterized benchmark. Several considerations support retaining this evaluation:

1. **All compared methods face the same overlap.** Boltz-2 is trained on all public domain data; AEV-PLIG was trained and augmented with BindingNet structures specifically to improve FEP benchmark performance [Valsson et al., 2025]. The

comparison is therefore conducted on a level playing field, and the relative ranking of methods remains informative even if absolute metrics are inflated.

2. **Congeneric series test relative ranking, not absolute prediction.** The Schrodinger FEP+ dataset [Ross et al., 2023] was specifically designed to evaluate the ability to rank-order closely related analogs—the task most relevant to lead optimization in drug discovery. This is a meaningful capability to benchmark regardless of training set overlap, as it tests sensitivity to fine-grained molecular differences rather than coarse target recognition.

However, Sections 2.6 and 2.7 substantiate the central thesis of the paper: that zero-shot generalization across arbitrary SAR landscapes is unrealistic, and that the practical value of FLOWR.ROOT lies in its capacity for efficient domain adaptation via LoRA finetuning.

Comment 1.4 – Foundation Model Positioning

The manuscript positions FLOWR.ROOT as a foundation model. However, the results on proprietary datasets show that zero-shot generalization fails and that meaningful performance requires project-specific fine-tuning. While fine-tuning is common practice, this behavior does not by itself demonstrate foundation-level transfer capability. The manuscript should clarify this positioning and avoid overstating generalization claims unless additional evidence of cross-domain robustness is provided.

Response:

We thank the reviewer for raising this point. **Definition of "foundation model."** The term *foundation model* was introduced by Bommasani et al. [2021] and is defined as "[...] *any model that is trained on broad data (generally using self-supervision at scale) that can be adapted (e.g., fine-tuned) to a wide range of downstream tasks.*" Critically, the same report explicitly states that foundation models are "[...] *intermediary assets; they are unfinished and generally should not be used directly, instead requiring adaptation for specific downstream tasks. The de facto approach for adaptation has been fine-tuning.*" The authors further explain the naming choice: "[...] the word "foundation" specifies the role these models play: a foundation model is itself incomplete but serves as the common basis from which many task-specific models are built via adaptation".

This definition centers on two properties: (1) broad pre-training on diverse data at scale, and (2) adaptability to downstream tasks via fine-tuning. Notably, zero-shot generalization is **not** part of this definition—it is neither a necessary nor a defining characteristic of the foundation model paradigm. Indeed, the canonical foundation models—BERT [Devlin et al., 2019] and GPT-1 [Radford et al., 2018]—possess no zero-shot capability and require task-specific fine-tuning for every application. Both are universally recognized as foundation models [Bommasani et al., 2021].

FLOWR.ROOT is pre-trained on ∼1.5 billion small molecule conformations and ∼2.5 million mixed-fidelity protein–ligand complexes spanning diverse protein families, assay types, and chemical series. This broad pre-training enables parameter-efficient adaptation to a wide range of downstream tasks: *de novo* generation, interaction-conditional generation, scaffold hopping, fragment growing, multi-endpoint affinity prediction, and multi-target selectivity optimization, which constitutes the "broad pre-training → adaptation" paradigm that defines foundation models.

Both Boltz-2 and FLOWR.ROOT fail to generalize zero-shot to proprietary structure–

activity landscapes (Section 2.6, Fig. 5a–b), despite strong performance on public benchmarks. This is not unique to our model—it reflects a fundamental challenge in computational drug discovery: potency and binding affinity prediction depends on assay- and series-specific structure–activity relationships that cannot be captured from public training data alone. Therefore, we argue as one of the main contributions of this work that the failure of zero-shot predictions in SAR predictions is a domain property and not a model deficiency. In contrast, zero-shot structure generation produces physically realistic ligands with high PoseBusters-validity even on out-of-distribution systems (Supplementary Tables 1–3), demonstrating that the model has learned transferable geometric, structural priors.

However, the key evidence that FLOWR.ROOT has learned transferable, reusable representations is the effectiveness of parameter-efficient LoRA fine-tuning [Hu et al., 2022]. LoRA was developed specifically because adapting foundation models is the standard workflow—it was designed to make the adaptation of foundation models more practical. Using only $\sim$3M trainable parameters ($\sim$9% of the full model), LoRA transforms poor zero-shot performances into strong positive correlations across all four proprietary projects and one public dataset. If the pre-trained representations were not transferable, low-rank, linear updates to a small fraction of parameters could not produce these significant improvements. The magnitude of this improvement with minimal parameter updates is what distinguishes a foundation model from a model trained from scratch.

Changes to the manuscript. Following the reviewer's suggestion, we have added a discussion of this to Supplementary Information—Methods. We use "foundation model" to describe the broader framework, and clarify that the foundation model property of FLOWR.ROOT refers to its broad pre-training and efficient adaptability—not to an expectation of universal zero-shot generalization across, e.g., arbitrary bioactivity landscapes.

Reviewer #2

General Assessment

I thank the authors for addressing the points raised in a comprehensive manner. Especially the revised structure of the manuscript works well.

We thank Reviewer #2 once again for the constructive feedback that helped to improve this manuscript.

Minor Comments

Comment 2.1 – Introduction Readability

The revised introduction section is a little dense, with very complex terminology. I would recommend, in the interest of general readability, to simplify the words a bit. Maybe some language editing may be helpful.

Response:

We thank the reviewer for this suggestion. We have revised the Introduction to improve readability and accessibility for a broader audience.

Comment 2.2 – Figure 1 Density

Figure 1 is also very dense. Would recommend to simplify.

Response:

We thank the reviewer for this suggestion. We have cleaned up and slightly redesigned Figure 1.

Reviewer #3

General Assessment

My comments have been addressed reasonably well.

We thank Reviewer #3 once again for the constructive feedback that helped to improve this manuscript.

References

Rishi Bommasani, Drew A Hudson, Ehsan Adeli, Russ Altman, Simran Arora, Sydney von Arx, Michael S Bernstein, Jeannette Bohg, Antoine Bosselut, Emma Brunskill, et al. On the opportunities and risks of foundation models. *arXiv preprint arXiv:2108.07258*, 2021.

Michael Crawshaw. Multi-task learning with deep neural networks: A survey. *arXiv preprint arXiv:2009.09796*, 2020.

Jacob Devlin, Ming-Wei Chang, Kenton Lee, and Kristina Toutanova. BERT: Pre-training of deep bidirectional transformers for language understanding. *arXiv preprint arXiv:1810.04805*, 2019.

Wei-Ting Hsu, Stanislav Grevtsev, Arne M. Herz, Thomas Douglas, Aniket Magarkar, and Philip C. Biggin. Can AI-predicted complexes teach machine learning to compute drug binding affinity? *Journal of Chemical Information and Modeling*, 65(24):13051–13056, 2025. doi: 10.1021/acs.jcim.5c01848.

Edward J Hu, yelong shen, Phillip Wallis, Zeyuan Allen-Zhu, Yuanzhi Li, Shean Wang, Lu Wang, and Weizhu Chen. LoRA: Low-rank adaptation of large language models. In *International Conference on Learning Representations*, 2022. URL `https://openreview.net/forum?id=nZeVKeeFYf9`.

Dhruv Mahajan, Ross Girshick, Vignesh Ramanathan, Kaiming He, Manohar Paluri, Yixuan Li, Ashwin Bharambe, and Laurens van der Maaten. Exploring the limits of weakly supervised pretraining. In *Proceedings of the European Conference on Computer Vision (ECCV)*, pages 181–196, 2018.

Michael McCabe, Bruno Régaldo-Saint Blancard, Liam Holden Parker, Ruben Ohana, Miles Cranmer, Alberto Bietti, Michael Eickenberg, Siavash Golkar, Geraud Krawezik, Francois Lanusse, Mariel Pettee, Tiberiu Tesileanu, Kyunghyun Cho, and Shirley Ho. Multiple physics pretraining for physical surrogate models. In *Advances in Neural Information Processing Systems*, volume 36, 2023.

Alec Radford, Karthik Narasimhan, Tim Salimans, and Ilya Sutskever. Improving language understanding by generative pre-training. 2018. OpenAI.

Gregory A. Ross, Chao Lu, Guido Scarabelli, and Lingle Wang. The maximal and current accuracy of rigorous protein–ligand binding free energy calculations. *Communications Chemistry*, 6:222, 2023. doi: 10.1038/s42004-023-01019-9.

Sebastian Ruder. An overview of multi-task learning in deep neural networks. *arXiv preprint arXiv:1706.05098*, 2017.

Ísak Valsson, Matthew T. Warren, Charlotte M. Deane, Aniket Magarkar, Garrett M. Morris, and Philip C. Biggin. Narrowing the gap between machine learning scoring functions and free energy perturbation using augmented data. *Communications Chemistry*, 8(1):41, Feb 2025. ISSN 2399-3669. doi: 10.1038/s42004-025-01428-y. URL `https://doi.org/10.1038/s42004-025-01428-y`.

Jason Yosinski, Jeff Clune, Yoshua Bengio, and Hod Lipson. How transferable are features in deep neural networks? In *Advances in Neural Information Processing Systems*, volume 27, pages 3320–3328, 2014.

Rebuttal Letter — Third Revision

FLOWR.root: A flow matching based foundation model for joint multi-purpose structure-aware 3D ligand generation and affinity prediction

Manuscript ID: NCOMMS-25-95570B

Nature Communications

Dear Reviewer,

Thank you for the continued constructive engagement with our manuscript. We address each remaining point in detail below.

Sincerely,
The Authors

Reviewer #1

General Assessment

The revised manuscript is clearer and better organized, and the additional explanations of the framework and training procedure are appreciated. While the manuscript has improved in clarity, the key methodological novelty concerns raised in the previous round remain still only partially addressed. Here are the major points:

We thank Reviewer #1 for acknowledging the improvements in clarity and organization, and for the continued engagement with our work. We address each remaining point in detail below.

Major Comments

Comment 1.1 – Controlled Validation of the Joint Architecture

The most important issue is the lack of a controlled validation of the joint structure–affinity architecture. While the authors clarify that they do not claim the affinity head improves structural generation, this does not address whether the joint formulation is necessary, or whether it introduces any interaction between tasks. The current arguments rely on indirect evidence and comparisons that do not isolate the role of the affinity head. Even a reduced-scale or proxy ablation would be important to justify this central design choice.

Response:

We ran a proxy ablation on HiQBind: a structure-only variant (affinity head and affinity loss removed) and the joint Flowr.root variant, trained under identical backbone, data, hyperparameters, and seeds. On the HiQBind test set the two models are within seed-level

variation on PB-validity, strain energy, Vina score, and diversity. The joint model takes modestly longer to converge, as expected for a multi-task objective. The joint formulation therefore does not degrade structural generation. We added this to the main text.

We also note that the affinity head is, by design, not isolatable as a swappable component: the downstream applications of FLOWR.ROOT require the affinity signal to be defined on the same generative distribution as the structure head (learned joint probability distribution). Specifically, (i) importance-sampling-based trajectory reweighting (Section 2.4) acts on intermediate states of that distribution and cannot be performed by a post-hoc external scorer; (ii) LoRA fine-tuning (Sections 2.6–2.7) updates structure and affinity coherently in the shared backbone, whereas two independent models would adapt on disjoint objectives with no guarantee of representational alignment increasing the risk of overfitting on the respective task, and thus the joint formulation acts as a natural regularization; (iii) multi-target selectivity (Section 2.8) and affinity-annotated fragment elaboration (Section 2.9) operate on the joint distribution by construction; and (iv) a single forward pass replaces a generate–rescore–filter loop, which substantially increases computational feasibility and reduces both training and inference times.

Comment 1.2 – Foundation-Model Positioning vs. Modular Pipeline

The manuscript positions the method as a unified or foundation-level framework, but the current evidence suggests that the work is primarily an integration of existing components rather than a clearly demonstrated conceptual advance. It remains unclear what new capability arises specifically from the joint formulation, and how it compares to a modular pipeline combining generation with external scoring.

Response:

The conceptual content of the joint formulation is that the affinity signal is defined on, and backpropagates into, the same shared backbone that defines the generative distribution—the model learns a joint probability distribution over structure and affinity. A modular generator-plus-external-scorer pipeline cannot replicate this without re-introducing the distribution mismatch and the multi-model overhead and complexity that the joint formulation is designed to remove. This gives rise to capabilities needed it drug-discovery campaigns and that the we show directly, namely (i) multi-objective optimisation via importance-sampling-based trajectory reweighting (Section 2.4), which in turn enables (ii) multi-target selectivity from a single model (CK2α/CLK3, Section 2.8); (iii) coherent joint LoRA adaptation of structure and affinity to a project-specific SAR landscape (Sections 2.6–2.7); and (iv) affinity-guided fragment elaboration (Section 2.9). We added a clarification to the main text.

We thank the reviewer for the constructive overall assessment.
